# Mapping heterogeneity in family planning indicators in Burkina Faso, Kenya, and Nigeria, 2000–2020

**DOI:** 10.1186/s12916-023-03214-w

**Published:** 2024-02-01

**Authors:** GBD Local and Small Area Estimation Family Planning Collaborators, Doori Oh, Rebecca M. Cogen, Erin C. Mullany, Susan McLaughlin, Olumide Abiodun, Lawan Hassan Adamu, Abiola Victor Adepoju, Miracle Ayomikun Adesina, Daniel Adedayo Adeyinka, Aanuoluwapo Adeyimika Afolabi, Olufemi Ajumobi, Dickson A. Amugsi, Olivia Angelino, Tesleem Kayode Babalola, Manaseh A. Bocha, Isaac Sunday Chukwu, Michael Ekholuenetale, Adeniyi Francis Fagbamigbe, Prof Morenike Oluwatoyin Folayan, Prof Muktar A. Gadanya, Augustine Mwangi Gatotoh, Annie Haakenstad, Prof Simon I. Hay, Segun Emmanuel Ibitoye, Olayinka Stephen Ilesanmi, Kenneth Chukwuemeka Iregbu, Charity Ehimwenma Joshua, Gbenga A. Kayode, Peter M. Macharia, Shafiu Mohammed, Aggrey Gisiora Mokaya, Prof Christopher J. L. Murray, Josephine W. Ngunjiri, Julius Nyerere Odhiambo, Oluwakemi Ololade Odukoya, Onome Bright Oghenetega, Abiola Ogunkoya, Akinkunmi Paul Okekunle, Patrick Godwin Okwute, Andrew T. Olagunju, Babayemi Oluwaseun Olakunde, Isaac Iyinoluwa Olufadewa, Bolajoko Olubukunola Olusanya, Jacob Olusegun Olusanya, Prof Obinna E. Onwujekwe, Prof Mayowa O. Owolabi, Mu’awiyyah Babale Sufiyan, Shehu Salihu Umar, Chukwuma David Umeokonkwo, Yohannes Dibaba Wado, Hadiza Yusuf, Laura Dwyer-Lindgren

**Affiliations:** 1 Institute for Health Metrics and Evaluation, University of Washington, Seattle, WA USA; 2 Department of Health Metrics Sciences, School of Medicine, University of Washington, Seattle, WA USA

**Keywords:** Contraceptive use, Family planning, Unmet need, Intention to use contraception, Geostatistics, Bayesian statistics, Subnational, Burkina Faso, Kenya, Nigeria

## Abstract

**Background:**

Family planning is fundamental to women’s reproductive health and is a basic human right. Global targets such as Sustainable Development Goal 3 (specifically, Target 3.7) have been established to promote universal access to sexual and reproductive healthcare services. Country-level estimates of contraceptive use and other family planning indicators are already available and are used for tracking progress towards these goals. However, there is likely heterogeneity in these indicators within countries, and more local estimates can provide crucial additional information about progress towards these goals in specific populations. In this analysis, we develop estimates of six family indicators at a local scale, and use these estimates to describe heterogeneity and spatial–temporal patterns in these indicators in Burkina Faso, Kenya, and Nigeria.

**Methods:**

We used a Bayesian geostatistical modelling framework to analyse geo-located data on contraceptive use and family planning from 61 household surveys in Burkina Faso, Kenya, and Nigeria in order to generate subnational estimates of prevalence and associated uncertainty for six indicators from 2000 to 2020: contraceptive prevalence rate (CPR), modern contraceptive prevalence rate (mCPR), traditional contraceptive prevalence rate (tCPR), unmet need for modern methods of contraception, met need for family planning with modern methods, and intention to use contraception. For each country and indicator, we generated estimates at an approximately 5 × 5-km resolution and at the first and second administrative levels (regions and provinces in Burkina Faso; counties and sub-counties in Kenya; and states and local government areas in Nigeria).

**Results:**

We found substantial variation among locations in Burkina Faso, Kenya, and Nigeria for each of the family planning indicators estimated. For example, estimated CPR in 2020 ranged from 13.2% (95% Uncertainty Interval, 8.0–20.0%) in Oudalan to 38.9% (30.1–48.6%) in Kadiogo among provinces in Burkina Faso; from 0.4% (0.0–1.9%) in Banissa to 76.3% (58.1–89.6%) in Makueni among sub-counties in Kenya; and from 0.9% (0.3–2.0%) in Yunusari to 31.8% (19.9–46.9%) in Somolu among local government areas in Nigeria. There were also considerable differences among locations in each country in the magnitude of change over time for any given indicator; however, in most cases, there was more consistency in the direction of that change: for example, CPR, mCPR, and met need for family planning with modern methods increased nationally in all three countries between 2000 and 2020, and similarly increased in all provinces of Burkina Faso, and in large majorities of sub-counties in Kenya and local government areas in Nigeria.

**Conclusions:**

Despite substantial increases in contraceptive use, too many women still have an unmet need for modern methods of contraception. Moreover, country-level estimates of family planning indicators obscure important differences among locations within the same country. The modelling approach described here enables estimating family planning indicators at a subnational level and could be readily adapted to estimate subnational trends in family planning indicators in other countries. These estimates provide a tool for better understanding local needs and informing continued efforts to ensure universal access to sexual and reproductive healthcare services.

**Supplementary Information:**

The online version contains supplementary material available at 10.1186/s12916-023-03214-w.

## Background

Contraception is an important means for achieving one’s desired goals with respect to family planning. In 2019, 51.9% of women around the world between the age of 15 and 49 years were using some method of contraception, an increase of 18.7 percentage points from the year 1970. However, 8.3% of women nonetheless still had an unmet need for any method of contraception [1]. Increased access to and use of contraception is associated with a variety of positive outcomes beyond the immediate objective of enabling women to achieve their family planning goals, including reduced maternal and neonatal mortality [2–4], as well as social and economic benefits related to women’s empowerment and increased paid employment [5].

Consequently, increasing access and decreasing unmet need for contraception has been an explicit target of several broad international development initiatives, including the Millennium Development Goals (Goal 5: “Improve maternal health”; Target 5.B: “Achieve, by 2015, universal access to reproductive health”) [6], and the Sustainable Development Goals (Goal 3: “Ensure healthy lives and promote well-being for all at all ages”; Target 3.7: “By 2030, ensure universal access to sexual and reproductive healthcare services, including for family planning, information and education, and the integration of reproductive health into national strategies and programmes.”) [7]. Additionally, there are other international initiatives and partnerships focused more exclusively on family planning, including Family Planning 2020 (FP2020)—whose primary objective was to increase the number of women using modern contraceptive methods by 120 million between 2012 and 2020 in 69 focus countries—and its successor Family Planning 2030 (FP2030) [8]. While these initiatives have coincided with notable increases in contraceptive use—particularly use of modern methods—and corresponding decreases in unmet need, the number of women around the world with an unmet need for contraception is still unacceptably high [1]. Timely, comprehensive, and geographically detailed data on trends and patterns in family planning are required to identify populations in greatest need and to help ensure equitable, continued progress towards reducing unmet need for contraception.

Country-level time-series estimates of family planning indicators are available on a global scale from several sources, including the Global Burden of Disease Study (GBD) [1, 9] and the United Nations Population Division [10–13]. In contrast, there are no comprehensive time-series estimates of family planning indicators on a subnational scale. Since 2016, the Demographic and Health Survey (DHS) Program has routinely created geospatial estimates of modern contraceptive use, met need for family planning with modern methods, and unmet need for any method of contraception, providing a cross-sectional view of spatial variation in these indicators in the countries and years where DHS surveys have been conducted [14, 15]. However, these estimates are not designed for investigating changes over time in family planning indicators and are only as timely as the most recently released DHS survey. Several other studies have generated subnational time-series estimates of selected family planning indicators for particular countries, including an analysis in Kenya of county-level trends in the modern contraceptive prevalence rate based on data from three survey series [16]; an analysis in Nigeria of state-level trends in the modern contraceptive prevalence rate, the traditional contraceptive prevalence rate, unmet need for any method of contraception, and met need for family planning with modern methods based on data from four survey series [17] and an analysis in 26 countries (selected based on availability of two or more DHS surveys) of trends at the second administrative level in the modern contraceptive prevalence rate, the traditional contraceptive prevalence rate, unmet need for any method of contraception, and met need for family planning with modern methods based on data from DHS surveys only [18]. Across countries and indicators, these studies have consistently found substantial variation within countries in both prevalence and change in prevalence over time, underscoring the need for fine-scale time-series data. However, availability of these types of estimates remains limited, and earlier subnational studies have typically not leveraged all available and relevant survey data.

In this study, we present estimates of six family planning indicators (contraceptive prevalence rate, modern contraceptive prevalence rate, traditional contraceptive prevalence rate, unmet need for modern methods of contraception, met need for family planning with modern methods, and intention to use contraception) in Burkina Faso, Kenya, and Nigeria from 2000 to 2020. Our estimates were generated at an approximately 5 × 5-km resolution and at the first and second administrative levels (13 regions and 45 provinces in Burkina Faso; 47 counties and 292 sub-counties in Kenya; and 37 states and 774 local government areas [LGAs] in Nigeria), and are based on a wide array of survey data sources. We intend this analysis as a proof-of-concept for a methodology for estimating family planning indicators on a subnational scale using all available survey data. Burkina Faso, Kenya, and Nigeria were selected as the focus countries for this analysis because they vary substantially in terms of levels of contraceptive use, unmet need, and met need at the national level; the amount and types of survey data available; and the number and size of the subnational areas. Thus, these three countries represent a range of different contexts where this methodology may be useful for future research. Moreover, to our knowledge, the findings presented here are the first time-series estimates of these six family planning indicators available at a 5 × 5-km resolution, and also the first time-series estimates of intention to use at any subnational spatial resolution.

## Methods

This study follows the Guidelines for Accurate and Transparent Health Estimates Reporting (GATHER) (Additional file 1: Sect. 1). An overview of the analytic approach is available in Additional file 1: Figure S1. Each of the steps is summarized below and further details are available in Additional file 1: Sects. 2 (data) and 3 (statistical model).

### Definition of indicators

The six indicators that we estimated—contraceptive prevalence rate (“CPR”), modern contraceptive prevalence rate (“mCPR”), traditional contraceptive prevalence rate (“tCPR”), unmet need for modern methods of contraception (“unmet need”), met need for family planning with modern methods (“met need”), and intention to use contraception (“intention to use”)—are defined in Table 1.

**Table 1:** Family planning indicators

**Indicator**	**Population**	**Indicator**	**Relationships**
Contraceptive prevalence rate (“CPR”)	All women ages 15–49	Proportion currently using any method(s) of contraception	CPR = mCPR + tCPR
Modern contraceptive prevalence rate (“mCPR”)	All women ages 15–49	Proportion currently using at least one modern^a^ method of contraception	
Traditional contraceptive prevalence rate (“tCPR”)	All women ages 15–49	Proportion currently using only traditional^a^ methods of contraception	
Unmet need for modern methods of contraception (“unmet need”)	All women ages 15–49	Proportion in need^b^ of family planning and not currently using any modern method(s) ^a^ of contraception	Met need = mCPR / (mCPR + unmet need)
Met need for family planning with modern methods (“met need”)	Women ages 15–49 in need^b^ of family planning	Proportion currently using any modern^a^ method(s) of contraception	
Intention to use contraception (“intention to use”)	Women ages 15–49 in need^b^ of family planning and not currently using any method(s) of contraception	Proportion who intend to use any method of contraception at any point in the future	

^a^Modern methods included contraceptive pills, condoms, diaphragms, spermicides and sponges, implants, injections, intrauterine devices, male and female sterilization, contraceptive patches, contraceptive rings, and emergency contraceptives. All other methods were considered traditional methods

^b^Women were considered “in need” of family planning if they were using any method of contraception; if they were pregnant and reported that their pregnancy was unwanted or wanted later; if they were postpartum amenorrheic following a birth in the previous 24 months and reported that their last pregnancy was unwanted or wanted later; or if they were not pregnant and not postpartum amenorrheic and reported that they wanted no or no additional children, were undecided about having a child or additional children, or desired a child or additional children but not for at least 2 years

Following the classification used by the GBD [1], modern methods of contraception included contraceptive pills, condoms, diaphragms, spermicides and sponges, implants, injections, intrauterine devices, male and female sterilization, contraceptive patches, contraceptive rings, and emergency contraceptives. All other methods were considered traditional methods, including periodic abstinence (rhythm method), standard days method (SDM/beads), lactational amenorrhea method (LAM), withdrawal, herbal concoctions, and charms and amulets. The determination of who is “in need” of family planning is complicated and varies for different groups of women. Women who reported current use of any method of contraception were considered to be in need. Among those who were not currently using any method of contraception, we considered three additional groups of women when determining who was in need of family planning: those who were currently pregnant, those who were postpartum amenorrheic following a birth in the previous 24 months, and all other women. Among women who were currently pregnant, those who reported that their current pregnancy was unwanted or wanted later were considered to be in need, while those who reported that their current pregnancy was wanted at that time were considered to be not in need. Similarly, among women who were postpartum amenorrheic following a birth in the previous 24 months, those who reported that their last pregnancy was unwanted or wanted later were considered to be in need, while those who reported that their last pregnancy was wanted at the time were considered to be not in need. Finally, among all other women, those who reported they wanted no or no additional children, were undecided about having a child or additional children, or desired a child but not for at least 2 years were considered to be in need, while those women who wanted a child within the next 2 years were considered to be not in need. Additionally, women who were not sexually active or were infecund were considered to be not in need. Women were assumed to be infecund if they reported that they could not have children, that they were menopausal, that they had had a hysterectomy, and/or that they had never menstruated. Additionally, women were assumed to be infecund if they had been married for at least 5 years, had never used contraception, and had never given birth; or if their last birth was at least 5 years prior and they had not had a period since before that birth. This definition of need for family planning is identical to the definition used by the GBD [1], and similar to that used by DHS [19]. In some cases, the definitions we use differ from what has previously been reported for a given survey; these differences are described in Additional file 1: Table S1.

### Data

We identified surveys for this analysis in three ways: via a keyword search in the Global Health Data Exchange, a publicly available data archive and repository; by review of the surveys used to estimate family planning indicators at the national level by the GBD [1, 9] and by Kantarova et al. [13]; and through manual review of websites for major survey series, namely, DHS [20], Multiple Indicator Cluster Surveys (MICS) [21], and Performance Monitoring for Action (PMA) [22]. We considered population-based surveys conducted between 1998 and 2020. We required that these surveys minimally include information on contraceptive use and contain geographic variables that provided some subnational detail. We identified 72 surveys that met these criteria; 61 were included in this analysis (Additional file 1: Table S2) while 11 surveys were excluded entirely (Additional file 1: Table S3). Additionally, five surveys were excluded from the calculations for specific indicators, and one survey had data from specific regions excluded from all analyses (Additional file 1: Table S3). Additional file 1: Figure S2 summarizes the data available by country and indicator.

For each survey included, we extracted demographic data; data related to contraceptive use, need for family planning, and intention to use contraceptives in the future; survey design variables; and geographic information. Additional file 1: Table S4 describes each of the survey items extracted. We then subset the extracted data to women ages 15–49, and, following the process described in Additional file 1: Figure S3, created variables that captured any contraceptive use, modern contraceptive use, need for family planning, and intention to use contraceptives in the future. Finally, we aggregated the data by calculating the survey-weighted prevalence and associated effective sample size (via the Kish approximation [23]) for each indicator at the most precise spatial resolution available. We encountered two types of spatial data. In some surveys, each survey cluster was associated with Global Positioning System (GPS) coordinates indicating the latitude and longitude (often with some random displacement to protect respondent confidentiality [24]); we refer to these data as “point data”. For surveys where cluster-level GPS coordinates were not available, we geo-located the survey microdata to the smallest geographical area indicated in the survey dataset, which was typically first- or second-level administrative areas; we refer to these data as “polygon data”. The statistical model that we used requires point data, so polygon data were “resampled” proportional to the underlying population in the corresponding area using methods developed and applied in previous studies using model-based geostatistics [25–29]. Further details on data processing and polygon resampling are given in Additional File 1: Sects. 2.1–2.2.

In addition to the survey data, we also utilized data on administrative boundaries from the Database of Global Administrative Areas (GADM) version 3.6 shapefiles [30] and gridded population estimates from WorldPop [31, 32], in both cases with minor modifications as described in Additional File 1: Sects. 2.3–2.4.

### Crosswalking

Survey questionnaires vary in ways that can impact our ability to measure contraceptive use, identify women in need of family planning, and/or ascertain a woman’s intentions regarding future contraceptive use—all of which can potentially limit the comparability of family planning indicators derived from different surveys. Specifically, there are four variations on this theme that we observed. First, there are surveys that limit questions related to sexual activity and contraceptive use to women who have ever been married. This impacts the calculation of all indicators. Second, there are surveys that do not include certain component questions required to determine need for family planning. This impacts the calculation of all indicators which are dependent on need, including intention to use contraceptives in the future, but does not impact calculation of contraceptive use prevalence. Third, there are surveys that ask about intention to use contraceptives only in the next 12 months, rather than at any point in the future. This impacts the calculation of intention to use contraceptives in the future. Finally, there are surveys that do not include any questions regarding intention to use contraceptives in the future. This final group of surveys were simply not included when modelling intention to use contraceptives. For the other three groups of surveys, we created a series of crosswalking models to adjust the survey data and account for differences in survey design, based on crosswalking methods developed for the GBD [33]. More details are available in Additional file 1: Sect. 2.5, and Table S5.

### Modelling strategy

The six family planning indicators that we estimated are mathematically related to each other in various ways (Table 1). Modelling each indicator separately is the most straightforward approach but does not guarantee consistency between the indicator estimates in terms of these relationships. We instead took an indirect approach that started by fitting separate statistical models for a series of four nested indicators: among all women, the proportion who are using contraceptives (${p}^{anycontra}$); among women who are using contraceptives, the proportion who are using modern methods (${p}^{modcontra}$); among women who are not using contraceptives, the proportion who are in need of family planning (${p}^{need}$); and among women who are not using contraceptives and are in need of family planning, the proportion who express an intent to use contraceptives in the future (${p}^{intent}$). This approach is conceptually similar to one used in prior research to estimate coverage of different numbers of doses of diphtheria-pertussis-tetanus vaccine [28].

We then recombined the estimates for these modelled indicators to delineate five mutually exclusive and collectively exhaustive groups: women who are not in need of family planning (${p}^{A}=\left(1-{p}^{anycontra}\right)\left(1-{p}^{need}\right)$); women who are in need of family planning but not currently using any method of contraception, and who do not express an intent to use contraceptives in the future (${p}^{B}=\left(1-{p}^{anycontra}\right)\cdot {p}^{need}\cdot {(1-p}^{intent})$); women who are in need of family planning but not currently using any method of contraception, and who express an intent to use contraceptives in the future (${p}^{C}=\left(1-{p}^{anycontra}\right)\cdot {p}^{need}\cdot {p}^{intent}$); women who are exclusively using traditional contraceptive methods (${p}^{D}={p}^{anycontra}\cdot (1-{p}^{modcontra})$); and women who are using modern contraceptive methods (${p}^{E}={p}^{anycontra}\cdot {p}^{modcontra}$). Additional file 1: Figure S4 summarizes the relationship between the four nested models and modelled indicators and these five groups of women.

Four of the final indicators correspond directly to one of the modelled indicators or the following groups: CPR = ${p}^{anycontra}$; mCPR = ${p}^{E}$; tCPR = ${p}^{D}$; and intention to use = ${p}^{intent}$. We calculated the remaining two indicators as: unmet need = ${p}^{B}+{p}^{C}+{p}^{D}$ and met need = ${p}^{E}/(1-{p}^{A}).$ Model fitting and the subsequent calculations were carried out independently for each of the three countries.

### Statistical model

We used a model-based geostatistical approach to estimate ${p}^{anycontra}, {p}^{modcontra}, {p}^{need}$ and ${p}^{intent}$. A similar approach has been previously used for estimating a variety of health-related indicators [25–29]. Separate models were fitted for each country and for each indicator, in two stages. In the first stage, we used an ensemble modelling approach known as stacked generalization to leverage available covariate data in the estimation of each indicator, allowing for possible non-linear effects and/or interactions among covariates [34, 35]. Specifically, we fit three sub-models to the survey data with five covariates as explanatory predictors (night time lights [36, 37], travel time to nearest settlement [38, 39], travel time to healthcare facilities [40, 41], population [31, 32], and mean years of education among women ages 15–49 [42, 43]). The three sub-models were as follows: generalized additive models using thin-plate regression splines as the smoothing function [44], fitted using the mgcv package [45, 46]; ridge regression [44], fitted using the glmnet package [47]; and boosted regression trees [48], fitted using the dismo package [49]. Additional details about the specification and implementation of the sub-models in the first stage are provided in Additional File 1: Sect. 3.1. In the second stage, we fit a Bayesian geostatistical model that included the estimates from the first stage models as predictors, in addition to spatially and temporally correlated random effects that allowed for additional variation in the outcome variable beyond what was explained by the included covariates. The full model specification and details on model fitting and validation are provided in Additional file 1: Sects. 3.2 [50–56], Figure S5, Tables S6–S7.

After fitting these models, we generated estimates of ${p}^{anycontra}$, ${p}^{modcontra}, {p}^{need}$ and ${p}^{intent}$ for each point on an approximately 5 × 5-km grid, and then using the calculations described above, we similarly generated estimates for the six final indicators at each point on this grid. We aggregated these grid-cell-level estimates to produce estimates at the second administrative level, first administrative level, and the national level by population-weighting the estimates for each grid cell within a given area. The population used for this aggregation varied by indicator, such that it matched the denominator of each indicator. For example, the denominator for met need is women who are in need of family planning; therefore, met need was aggregated using the number of women in need of family planning, which is calculated as $(population\;women\;ages\;15-49)\cdot\left(1-p^A\right)$. To propagate uncertainty from the various models through to the final estimates, we carried out this estimation procedure independently for each of 1000 “draws” (simulations) from the approximated posterior distribution of each model. We then generated point estimates from the mean and 95% uncertainty intervals from the 2.5th and 97.5th percentiles. For convenience, when considering change over time, we referred to change as “statistically significant” when the posterior probability that the difference was greater than 0 was either less than 2.5% or greater than 97.5% (analogous to a two-tailed test with *α* = 0·05). Pearson correlations ($\rho$) were used to quantify the strength of the linear relationship between different family planning indicators. Additional information on the prediction and aggregation strategy are provided in Additional file 1: Sect. 3.3.

## Results

### Burkina Faso

**Fig. 1:**
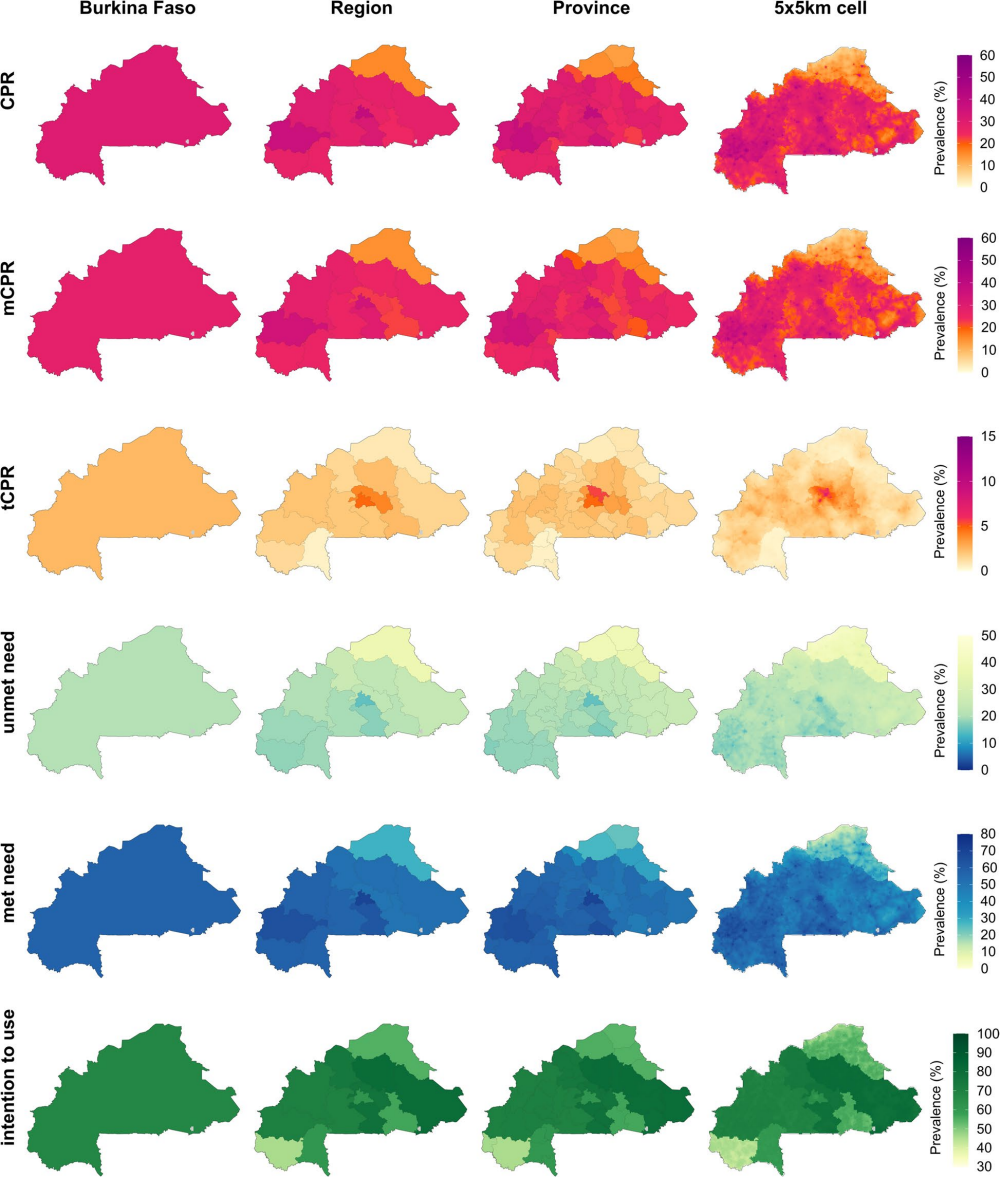
Estimated family planning indicators at the country, region, province, and 5 × 5-km grid-cell level in Burkina Faso, 2020. Bodies of water are coloured in light grey

Figure 1 shows estimates of all six indicators for Burkina Faso in 2020, the most recent year of this analysis (the corresponding uncertainty intervals are shown in Additional file 1: Figures S6–7; estimates in 2000 are shown in Figures S8–10). Nationally, we estimated 30.4% (95% uncertainty interval, 27.6–33.1%) of women ages 15–49 used some method of contraception, including 27.9% (25.2–30.6%) who used a modern method and 2.5% (1.9–3.3%) who exclusively used a traditional method. The contraceptive prevalence rate varied substantially by province, for example, ranging from 12.2% (7.2–19.2%) in Oudalan province, Sahel region in the north to 35.0% (27.0–43.2%) in Houet province, Haut-Bassins region in the west for mCPR. tCPR was relatively low in all locations, and consequently less variable, ranging from 0.5% (0.0–1.9%) in Ioba province, Sud-Ouest region in the southwest to 5.6% (2.4–10.5%) in Oubritenga province, Plateau-Central region in central Burkina Faso.

Nationally, in 2020, 20.8% (19.3–22.3%) of women of reproductive age had an unmet need for modern methods of contraception (unmet need), while 57.3% (53.9–60.8%) of women with a need for family planning had that need met with modern methods of contraception (met need). Again, these values varied widely by province, ranging from 14.8% (11.6–18.6%) in Kadiogo province, Centre region in central Burkina Faso to 39.3% (30.8–48.6%) in Soum province, Sahel region in the north for unmet need, and from 23.9% (14.6–35.1%) in Oudalan province, Sahel region to 69.5% (60.8–77.2%) in Kadiogo province, Centre region for met need. Unsurprisingly, areas with higher levels of mCPR also had higher levels of met need, while the reverse was true for unmet need. Among women in need of family planning but not using any method of contraception, 67.8% (62.5–72.9%) intended to use contraception at some point in the future (intention to use), the highest percentage among the three countries in this study. However, intention to use varied somewhat among provinces, ranging from 45.6% (24.8–67.7%) in Comoé province, Cascades region in the southwest to 81.3% (66.8–91.0%) in Sanmatenga province, Centre-Nord region in central Burkina Faso.

**Fig. 2:**
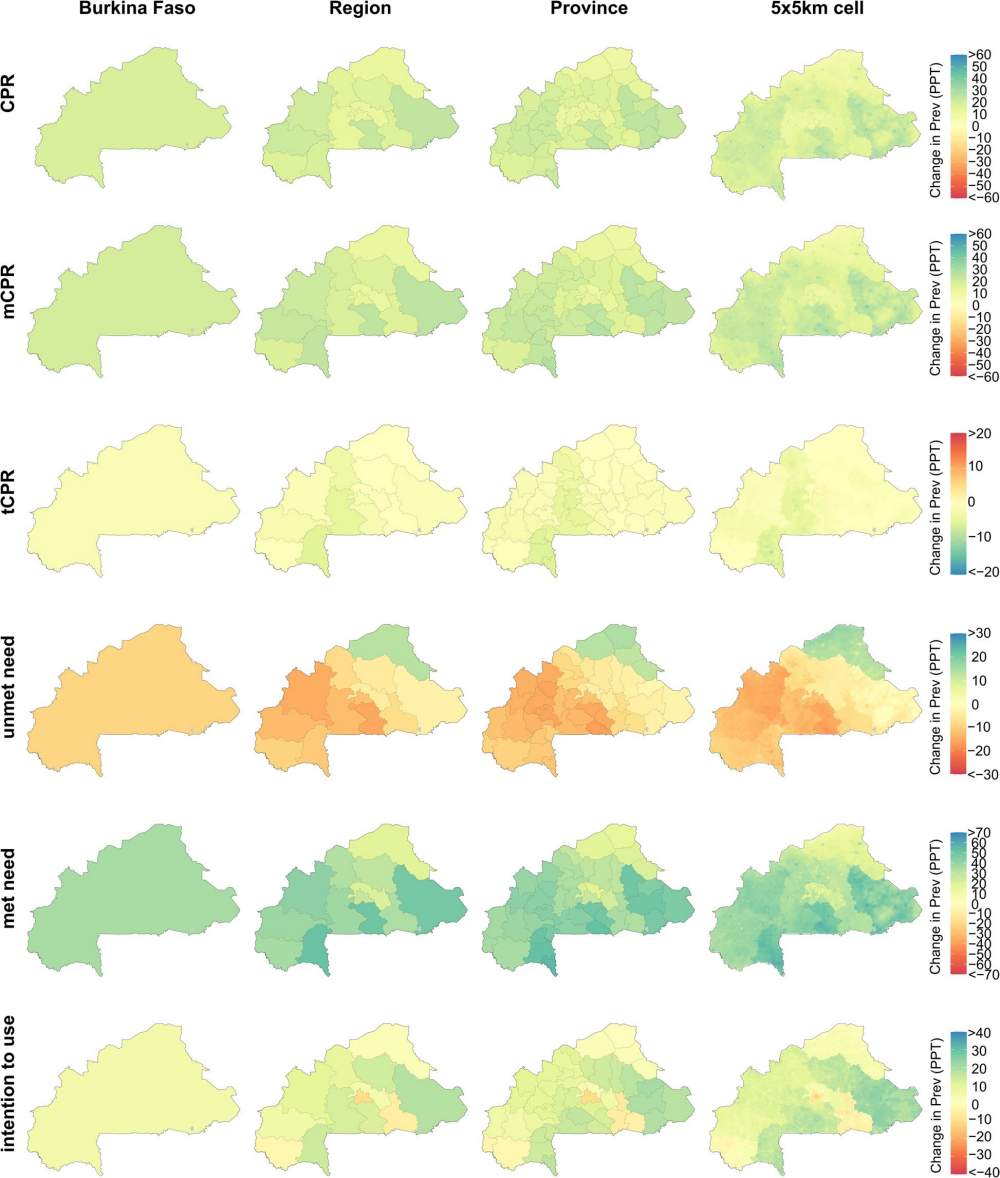
Estimated change in family planning indicators at the country, region, province, and 5 × 5-km grid-cell level in Burkina Faso, 2000–2020. Bodies of water are coloured in light grey

Figure 2 shows the estimated change in each of the six family planning indicators in Burkina Faso between 2000 and 2020. Nationally, CPR increased by 18.3 percentage points (PPT) (15.2–21.6 PPT), due a slightly larger gain in mCPR (19.7 PPT [16.9–22.9 PPT]) and a small decline in tCPR (− 1.4 PPT [− 2.5 to − 0.3 PPT]). The size of the change in CPR, mCPR, and tCPR varied geographically, but there was a great deal of consistency in the direction of the change—mCPR and CPR increased in all provinces (and these gains were statistically significant in all cases), whereas tCPR decreased in 38 out of 45 provinces (although these declines were statistically significant in only 12 out of 38 provinces) and increased in 7 out of 45 provinces (none of these increases were statistically significant). The widespread increases in mCPR drove similarly pervasive gains in met need (statistically significant increases in all provinces) and extensive declines in unmet need (declines estimated in 41 of 45 provinces; statistically significant in 17). Intention to use contraception in the future increased modestly at the national level, by 5.1 PPT (− 3.8 to 14.8 PPT), though we note that this estimate is highly uncertain. For most provinces (39 of 45), we similarly estimate that intention to use increased over this period, though in all cases this change was not statistically significant, reflecting the high degree of uncertainty in these estimates.

### Kenya

**Fig. 3:**
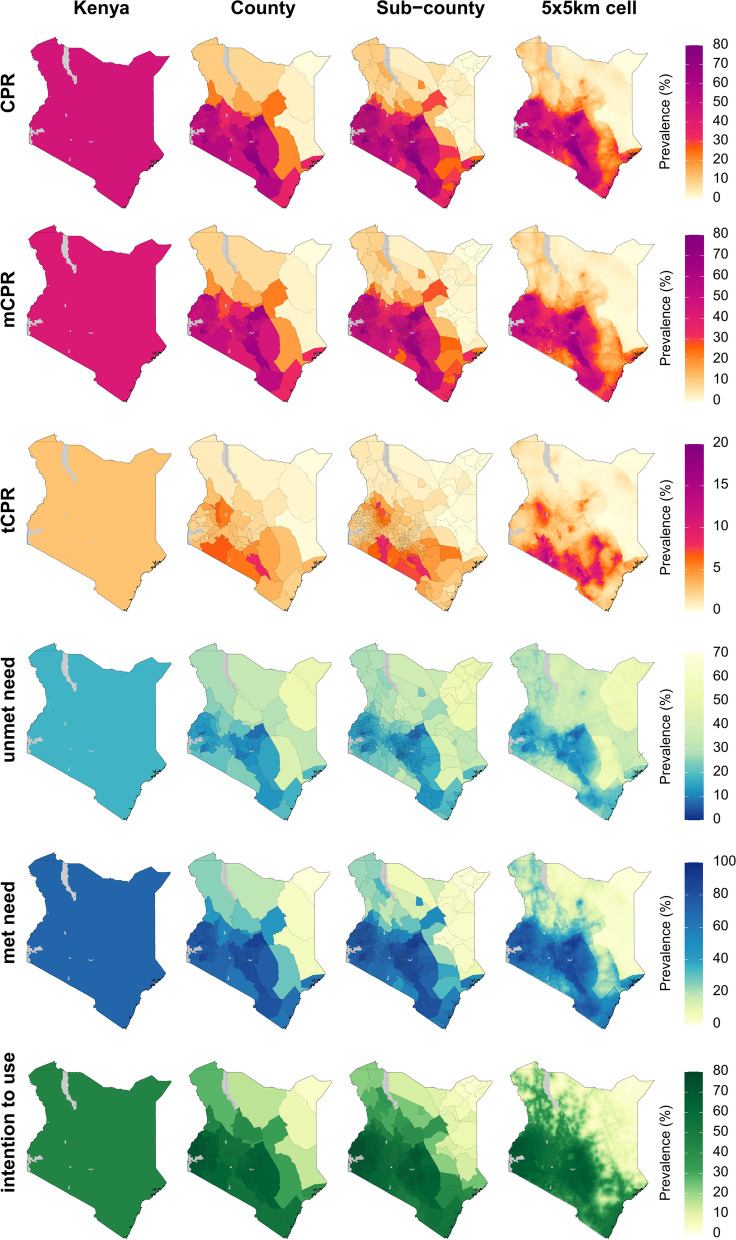
Estimated family planning indicators at the country, county, sub-county, and 5 × 5-km grid-cell level in Kenya, 2020. Bodies of water are coloured in light grey

Figure 3 shows estimates for family planning indicators in Kenya in 2020 (the corresponding uncertainty intervals are shown in Additional file 1: Figures S11–12; estimates in 2000 are shown in Figures S13–15). At the national level, we estimated that CPR was 45.7% (43.0–48.1%) among women of reproductive age, a large majority of whom were using modern methods, with mCPR estimated at 42.8% (38.3–46.2%) and tCPR at 2.9% (1.0–6.6%). There was considerable variation in CPR among sub-counties in Kenya, even greater than that observed among provinces in Burkina Faso and LGAs in Nigeria. Across sub-counties, CPR varied from 0.4% (0.0–1.9%) in Banissa sub-county, Mandera county in the northeast to 76.3% (58.1–89.6%) in Makueni sub-county, Makueni county in the south. mCPR also had substantial variation ranging from 0.3% (0.0–1.2%) in Lafey sub-county, Mandera county in the northwest to 70.8% (51.4–85.2%) in South Imenti sub-county, Meru county in central Kenya. As was the case in Burkina Faso, tCPR was relatively low in most locations in Kenya, and thus less variable, ranging from 0.1% (0.0–0.2%) in Lagdera sub-county, Garissa county in the east to 9.9% (3.3–22.0%) in Kilome sub-county, Makueni county in the south.

In 2020, 17.7% (15.3–21.7%) of all women of reproductive age in Kenya had an unmet need for modern methods of contraception, while 70.7% (64.0–74.9%) of women with a need for family planning had that need met with modern methods. Unsurprisingly, given the variation in mCPR, these values varied widely among sub-counties, ranging from 6.2% (2.7–11.4%) in North Imenti sub-county, Meru county in central Kenya, to 54.5% (33.7–75.7%) in Tarbaj sub-county, Wajir county in the northeast for unmet need, and from 0.7% (0.1–3.1%) in Lafey sub-county, Mandera county to 91.7% (83.0–96.6%) in North Imenti sub-county, Meru county for met need. Among women in need of family planning but not using any method of contraception, 44.6% (39.8–49.6%) intended to use contraception at some point in the future. Intention to use also varied subnationally, ranging from 2.9% (1.0–6.5%) in Banissa sub-county, Mandera county in the north to 73.1% (65.5–80.4%) in Funyula sub-county, Funyula county in the west. In contrast to Burkina Faso, where rates of intention to use were only moderately correlated with rates of contraceptive use ($\rho$ = 0.44 for CPR, and $\rho$ = 0.41 for mCPR), in Kenya, intention to use was strongly positively correlated with contraceptive use (0.88 for both CPR and mCPR).

**Fig. 4:**
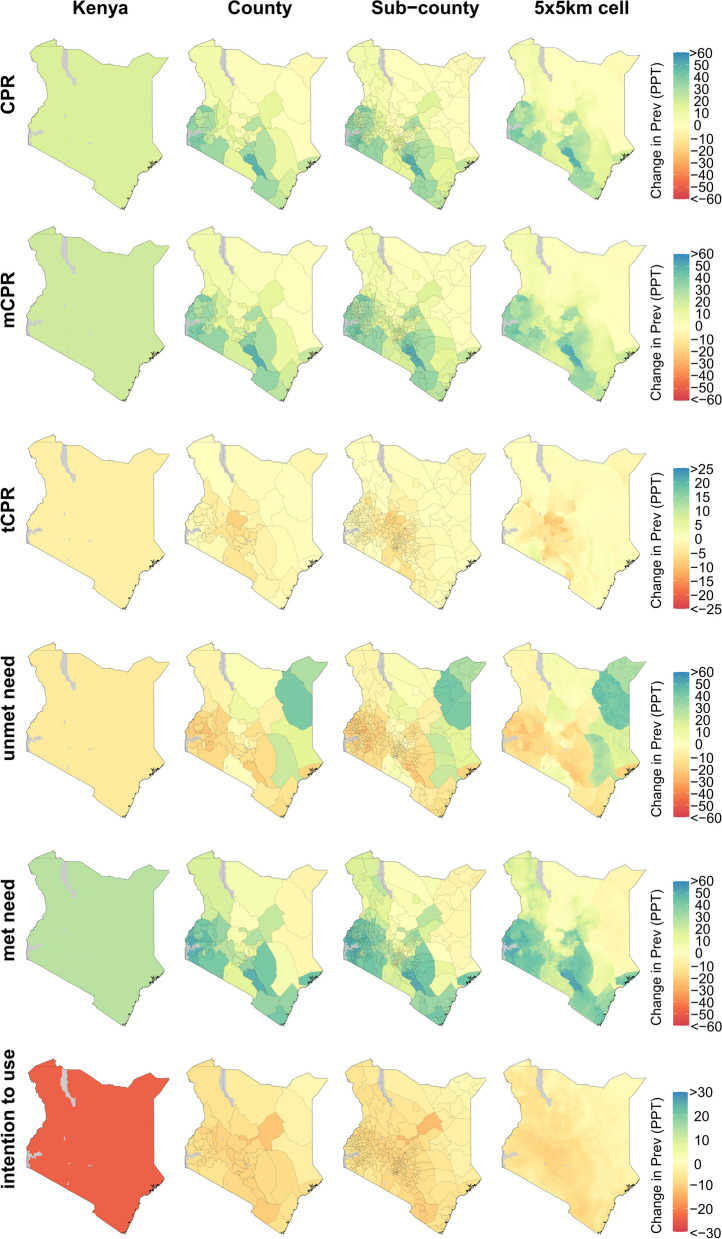
Estimated change in family planning indicators at the country, county, sub-county, and 5 × 5-km grid-cell level in Kenya, 2000–2020. Bodies of water are coloured in light grey

Figure 4 depicts the estimated change in each family planning indicator in Kenya between 2000 and 2020. At the national level, CPR increased by 18.1 PPT (14.9–21.2 PPT), corresponding to a larger increase in mCPR (21.2 PPT [15.4–27.9 PPT]) and a moderate decrease in tCPR (− 3.1 PPT [− 9.3 to 2.1 PPT]). As was the case in Burkina Faso, this gain in mCPR and decline in tCPR, which led to an increase in CPR overall, occurred throughout the country; nearly all sub-counties experienced increases in mCPR and CPR (282 [174 statistically significant] and 278 [157 statistically significant], respectively, out of 292), but observed decreases in tCPR, though these were generally not statistically significant (275 [4 statistically significant] out of 292). Consequently, gains in met need and declines in unmet need were similarly widespread. Although the direction of change for these indicators was very consistent across the country, the magnitude of change was much more variable. For example, met need decreased in Mandera East sub-county, Mandera county (− 8.4 PPT [− 30.5 to 2.1 PPT]) in the northeast, but increased dramatically in Alego Usonga sub-county, Siaya county (51.9 PPT [38.5–63.7 PPT]) in the west. Over this same period, intention to use declined nationally by − 24.5 PPT (− 31.6 to − 17.5 PPT); declines in intention to use were similarly observed in all sub-counties (292 [111 statistically significant] out of 292).

### Nigeria

**Fig. 5:**
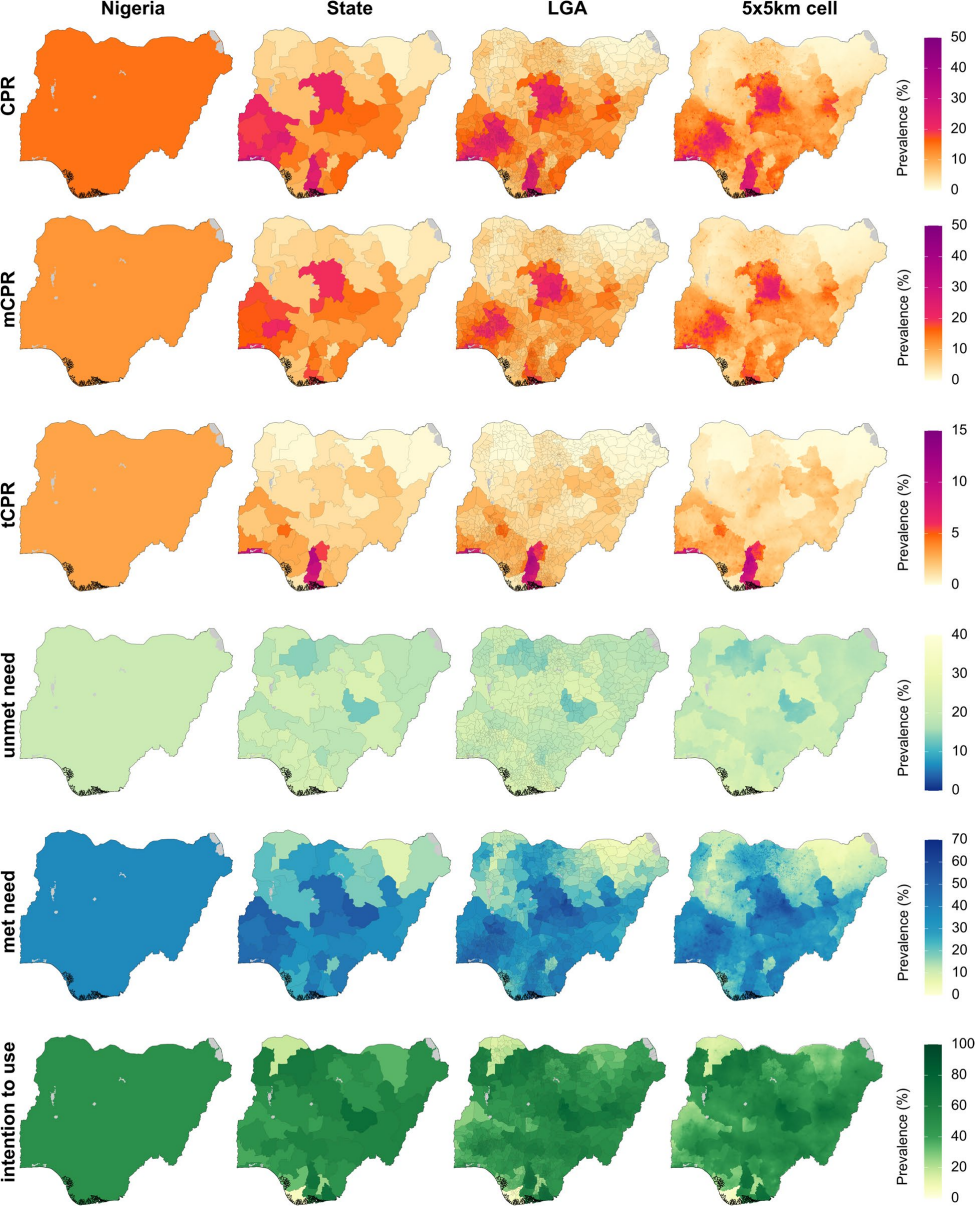
Estimated family planning indicators at the country, state, LGA, and 5 × 5-km grid-cell level in Nigeria, 2020. Bodies of water are coloured in light grey

Estimates for the six family planning indicators in 2020 for Nigeria are shown in Fig. 5 (the corresponding uncertainty intervals are shown in Additional file 1: Figures S16–17; estimates in 2000 are shown in Figures S18–20). Nationally, we estimate that CPR was 15.0% (13.3–16.7%), the lowest rate among the three countries in this analysis. Modern methods were most common, with mCPR at 11.9% (10.5–13.4%). However, exclusive use of traditional methods was nonetheless a sizeable portion of total contraceptive use, with tCPR at 3.1% (2.5–3.8%). All three contraceptive prevalence rate measures varied substantially among LGAs: from 0.9% (0.3–2.0%) in Yunusari LGA, Yobe state in the northeast to 31.8% (19.9–46.9%) in Somolu LGA, Lagos state in the southwest for CPR; from 0.7% (0.2–1.8%) in Yunusari LGA, Yobe state to 25.8% (14.9–39.0%) in Kaduna South LGA, Kaduna state in the northwest for mCPR; and from 0.1% (0.0–0.4%) in Abadam LGA, Borno state in the northeast to 11.2% (5.5–19.2%) in Ihiala LGA, Anambra state in the southeast, for tCPR.

In 2020, 20.4% (18.4–22.4%) of women of reproductive age in Nigeria had an unmet need for modern methods of contraception; however, this varied among LGAs from 12.2% (5.6–22.5%) in Jos North LGA, Plateau state in northcentral Nigeria to 28.1% (15.1–45.0%) in Mbo LGA, Akwa Ibom state in the south-south. Among women with a need for family planning, 36.9% (33.3–40.6%) had that need met with modern methods; however, this value varied substantially among LGAs, from 4.5% (1.1–13.0%) in Yunusari LGA, Yobe state to 61.5% (37.7–82.0%) in Jos North LGA, Plateau state. Among women in need of family planning who were not using any method of contraception, 49.0% (44.4–53.4%) intended to use contraception in the future, and this value ranged widely among LGAs, from 4.4% (1.2–11.7%) in Ekeremor LGA, Bayelsa state in the south-south to 78.5% (52.4–92.8%) in Jos North LGA, Plateau state. Similar to Burkina Faso, the rates of CPR and mCPR were low-to-moderately correlated to the rates of intention to use in Nigeria ($\rho$ = 0.30 and $\rho$ = 0.28, respectively).

**Fig. 6:**
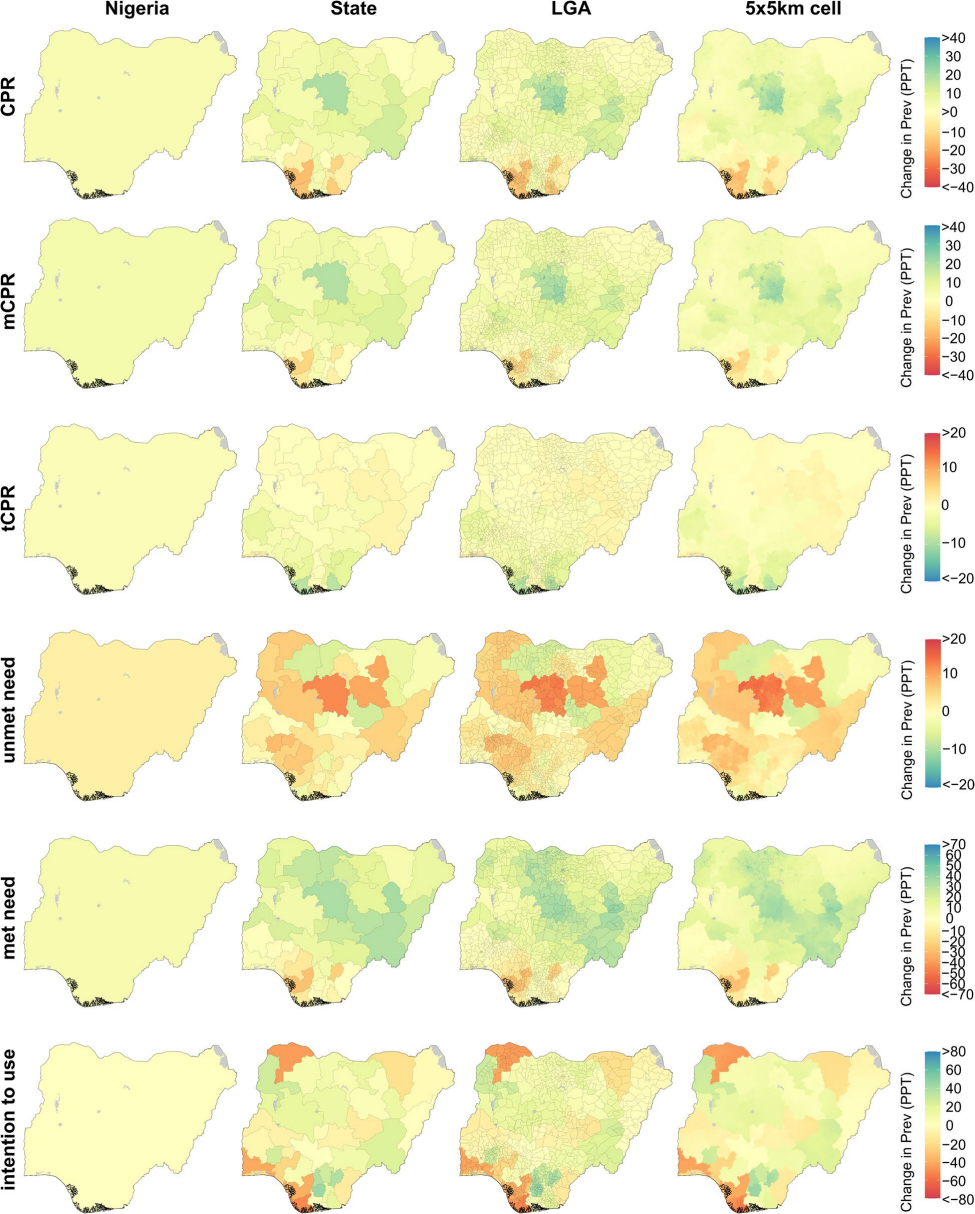
Estimated change in family planning indicators at the country, state, LGA, and 5 × 5-km grid-cell level in Nigeria, 2000–2020. Bodies of water are coloured in light grey

Estimated change in each indicator between 2000 and 2020 is shown for Nigeria in Fig. 6. Nationally, CPR increased by 3.3 PPT (0.9–5.8 PPT), driven almost entirely by an increase in mCPR 4.3 PPT (2.5–6.2 PPT), although we estimate a small decline in tCPR (− 1.0 PPT [− 2.0 to 0.1 PPT]). Similar to the situation in Burkina Faso and Kenya, the increases in CPR and mCPR observed nationwide reflected widespread increases in the same indicators at the local level; we estimated gains in CPR in 604 of 774 LGAs (statistically significant in 263) and in mCPR in 665 of 774 LGAs (statistically significant in 324). The direction of change in tCPR was more mixed among LGAs (447 where we estimate a decline; 327 where we estimate an increase); however, these changes were typically small in magnitude and in most cases not statistically significant. Gains in met need were similarly widespread, driven by the increase in mCPR. We also estimated gains in unmet need in 523 of 774 LGAs, though in all cases this change was not statistically significant. Between 2000 and 2020, we estimate a small increase in intention to use by 0.1 PPT (− 8.5 to 8.8 PPT); however, we note that this estimate was subject to a high degree of uncertainty. We also estimated an increase in intention to use in a majority of LGAs (482 of 774); however, these increases were in most cases not statistically significant (statistically significant in 4), reflecting the large degree of uncertainty in these estimates.

## Discussion

In this study, we present national- and subnational-level estimates for six family planning indicators—CPR, mCPR, tCPR, unmet need, met need, and intention to use—in Burkina Faso, Kenya, and Nigeria. We introduce a novel modelling framework that incorporates all available data while handling heterogeneity to generate fine-scale time-series estimates. The comprehensive, detailed estimates provide crucial evidence about within-country heterogeneity in trends in contraceptive use, met or unmet need, and intention to use contraceptives. These data can be used to target populations in greatest need of family planning and to further efforts to effectively and equitably reduce unmet need.

Our results show substantial heterogeneity in use of contraceptives (and consequently also in rates of met and unmet need) within each country, and underscore how national estimates may obscure important subnational trends. In each country, we identified regions with substantially lower-than-average CPR, mCPR, and met need, and higher-than-average unmet need. The degree of within-country variation was especially remarkable in Kenya: despite having the highest national CPR among the three countries we considered, CPR was extremely low in large swaths of the north and northeast, comparable or even lower than the lowest levels observed in Nigeria (typically in the north), the country with the lowest national CPR among the three considered.

A thorough investigation of the drivers of these patterns and disparities is beyond the scope of this analysis; however, previous research highlights several important individual and community-level factors that likely play a role. Cultural and religious factors are thought to impact rates of contraceptive use by influencing desired family size, acceptability of contraceptives, and women’s autonomy in decision-making related to family planning. For example, in Burkina Faso, large families are often desired, and men are typically the primary decision-makers regarding family size and family planning, such that men’s attitudes towards family size and contraceptive use can be a significant barrier to women’s use of contraceptives [57]. In Nigeria, large families are traditionally also preferred, which can lead to lower acceptance of contraceptive methods. Additionally, there is opposition to the use of contraceptives among some religious groups in Nigeria, particularly among Muslims in the north of the country, which likely explains much of the north–south gradient observed in this study and others in use of contraceptives [58–62]. Use of contraception is also closely linked to women’s education, empowerment, and autonomy. Women with higher levels of education and greater social and economic autonomy are more likely to use contraception and to have greater control over their reproductive health [63, 64]. Additionally, access to education and economic opportunities can empower women to negotiate safe and effective use of contraception with their partners [62]. Thus, part of the variation observed in use of contraception and unmet need is likely related to differences in women’s education and empowerment in different communities within each country. Not surprisingly, physical access to reproductive healthcare has also been shown to influence use of contraceptives—for example, increasing distance to health facilities is typically found to be associated with decreases in contraceptive use, although the magnitude of this relationship varies depending on context and can also differ depending on type of contraception (e.g. short- vs long-acting methods) [65, 66]. Thus differences in physical access to reproductive healthcare dictated by geography, access to different forms of transportation, and the density of health facilities and care providers likely also contribute to the spatial differences in contraceptive use and other family planning indicators observed within each country.

All three of the countries considered in this analysis have implemented multiple programmes and initiatives aimed at increasing access to family planning and other reproductive health services and increasing use of modern contraceptives [67–70]. This includes commitments on the part of each country to FP2020 and again to FP2030 [71]. These programmes and initiatives have likely contributed to some of the patterns observed in this study, e.g. increasing use of contraceptives in most locations across the three countries. While outside of the scope of this analysis, the data presented here may be useful for more thoroughly investigating the impact of particular programmes and policies, and, in especially, tracking progress to more equitable outcomes within and across countries. Moreover, by providing baseline information about contraceptive use, need, unmet need, and intention to use contraceptives, these data may be an important input to future efforts to tailor interventions to better meet local needs. Future research efforts should consider ways to further support these efforts—for example, marrying spatial approaches such as in this analysis, with other types of actionable detail, such as method mix, marital status, or age [1].

### Methodological advantages and limitations

Previous efforts to generate population estimates of family planning indicators have focused primarily at the national level [1, 9, 11–13], although studies have increasingly begun to disaggregate estimates, reporting separate values for married or in-union women and unmarried women [1, 12], and also for specific age groups [1, 9, 11–13]. A small but growing number of studies have concentrated on subnational variation in sub-Saharan Africa [16–18]. Our work is broadly consistent with this previous subnational research, both in the use of Bayesian hierarchical models and in the findings of substantial subnational variation in every country and for every family planning indicator considered. However, our study also introduced three methodological innovations that can be productively applied to future work evaluating access to family planning in specific populations. First, following the example of research efforts that have produced national-level estimates, the present study made use of all available survey data, in contrast to previous subnational studies that tend to rely exclusively on major survey series, particularly the DHS. Especially at subnational levels, where the amount of data contained in a given survey is necessarily limited for any particular location, expanding the number of surveys included can substantially increase the amount of data used to inform the estimates. The impact of including additional data varied widely depending on the country; in Burkina Faso, all of the surveys we identified belonged to one of four major series (DHS, MICS, PMA2020, and PMA), but in Kenya and Nigeria, we were able to incorporate three and ten additional surveys, respectively, that were not part of any of these four series. Second, to address heterogeneity in data structure and definitions across surveys, we leveraged crosswalking methods originally developed for national-level analyses. The consequence of using crosswalking techniques was relatively minor in the estimation of the contraceptive use indicators, as all but one of the surveys included in this analysis collected method-specific information on contraceptive use from all women of reproductive age. However, the impact was more substantial for the other indicators, as nearly one third (19 out of 61) of surveys included in the estimation of need for family planning were missing components essential for determining need. Crosswalking techniques enabled us to incorporate a much wider array of data sources than would otherwise have been possible. Finally, the nested modelling approach we developed, in lieu of estimating each family planning indicator independently, served to improve consistency across the six indicators. Additionally, the modelling approach we used allowed us to produce estimates on a 5 × 5-km grid in addition to estimates for administrative units. While we expect that the estimates at the second administrative level are likely to be sufficient for many use cases, the estimates on a 5 × 5-km grid highlight important additional subnational variation on a finer scale and may be useful in cases where there is interest in family planning indicators for a more local area or an area not delineated by administrative boundaries.

Despite these methodological innovations, this study was subject to several limitations. First, our analysis was inherently limited by the availability of the underlying data. While we made use of the broadest array possible of available survey data, there were nonetheless temporal and spatial gaps in data coverage (Additional file 1: Figure S2). In some cases, these gaps are more substantial than the number of surveys would suggest—for example, all of the MICS surveys in Kenya and all of the PMA and PMA2020 surveys in Kenya and Nigeria were subnational in scope and covered only limited areas of these countries. Second, our analysis was also limited by the quality of the underlying data. Survey data are subject to any number of biases, including non-response bias, and biases due to misreporting. Moreover, while we implemented crosswalks to adjust for appreciable structural and definitional differences across data, there were other more subtle differences in question wording and context that potentially led to differences in the reported rates of contraceptive use or need for family planning which we were unable to adjust for in this analysis. Other differences in the way surveys were designed, conducted, or processed (e.g. production of survey weights) may also have produced discrepancies in estimates across surveys that did not accurately reflect true differences in contraceptive use or need for family planning. Further, the geographical data included were subject to imprecision and error, most acutely in cases where we lacked access to cluster-level GPS data and instead used polygon data that were “resampled” to mimic point data. However, point data were also not immune from imprecision, as most surveys that collect GPS coordinates perform random displacement on these coordinates in order to safeguard respondent confidentiality [24]. However, although such displacement can impact the prediction accuracy of a geostatistical model, past research has determined that modelled estimates at a 5 × 5-km resolution are still relatively accurate even with this displacement [14]. Third, our modelling approach relied on “borrowing strength” across space and time and via the inclusion of covariates to inform estimates in locations and time periods with limited or no family planning data. We believe this approach was effective in most cases but there may have been circumstances when it was suboptimal. For example, if a policy or programme resulted in a sudden change in the contraceptive prevalence rate, this would have been unlikely to be reflected in our estimates unless there were substantial amounts of directly observed data in the relevant time period or location. Fourth, it is difficult to precisely assess the performance of these models given the lack of a “gold standard” to compare to, especially at more granular geographic levels. We used cross-validation to assess model performance; however, the results of this exercise should be interpreted cautiously, as the underlying data—which form the “gold standard” in cross-validation—are also subject to potential biases (as described above) and stochastic variation. Fifth, the estimates we generated were characterized by considerable levels of uncertainty (Additional file 1: Figures S6–7, S9–12, S14–17, S19–20), which limits the potential of our findings to conclusively identify small and even moderate differences in family planning indicators over time or between locations. Our results should therefore be interpreted cautiously, particularly when investigating changes over short time periods or attempting to rank order family planning estimates across subnational areas. Finally, no small set of indicators can capture all meaningful information about a topic as complex as family planning and contraceptive use; moreover, by themselves, these indicators do not answer the question of why and how the variation we observed came to be. Thus, when interpreting differences in these indicators across locations, and particularly across countries, it is important to also consider the historical and cultural context, in addition to any other relevant sources of information on family planning available for a given locale.

## Conclusions

Despite substantial increases in contraceptive use, too many women still have an unmet need for modern methods of contraception. Moreover, country-level estimates of family planning indicators obscure important differences among locations within the same country. The modelling approach described here enables estimating family planning indicators at a subnational level and could be readily adapted to estimate subnational trends in family planning indicators in other countries. These estimates provide a tool for better understanding local needs and informing continued efforts to ensure universal access to sexual and reproductive healthcare services.

### Supplementary Information

**Additional file 1: **Section 1 – GATHER table; Section 2 – further details on data and data processing; Section 3 – further details on the statistical model; **Figure S1.**  analytic process overview; **Figure S2.** data availability; **Figure S3.** indicator creation flowchart; **Figure S4.** modelled indicators; **Figure S5.** finite element mesh for SPDE approximation; **Figure S7–S8.** uncertainty bounds for Burkina Faso, 2020; **Figure S8–S10.** estimates and uncertainty bounds for Burkina Faso, 2000; **Figure S11–S12.** uncertainty bounds for Kenya, 2020; **Figure S13–S15.** estimates and uncertainty bounds for Kenya, 2000; **Figure S16–S17.** uncertainty bounds for Nigeria, 2020; **Figure S18–S20.** estimates and uncertainty bounds for Nigeria, 2000; **Table S1.** indicator values and definitions; **Table S2.** surveys included in this analysis; **Table S3.** – surveys excluded from this analysis; **Table S4.** extracted survey variables; **Table S5.** crosswalk parameters; **Table S6.** MBG model parameters; **Table S7.** validation results; Author’s contributions.

## Data Availability

Our study follows the Guidelines for Accurate and Transparent Health Estimates Reporting (GATHER). All estimates are available through an online visualization tool (https://vizhub.healthdata.org/family-planning) and via the Global Health Data Exchange (https://ghdx.healthdata.org/record/ihme-data/global-family-planning-estimates-2000-2020). The source code used to generate these estimates is available in GitHub (https://github.com/ihmeuw/fp_mapping). All maps presented in this study are generated by the authors and no permissions are required to publish them.
