# Supplementary material for: Mapping heterogeneity in family planning indicators in Burkina Faso, Kenya, and Nigeria, 2000–2020
Source: BMC Med. 2024 Feb 1;22:38. doi: 10.1186/s12916-023-03214-w (PMC10832137; doi:10.1186/s12916-023-03214-w)
Supplement: Supplementary file 1 — Additional file 1: Section 1 – GATHER table; Section 2 – further details on data and data processing; Section 3 – further details on the statistical model; Figure S1. analytic process overview; Figure S2. data availability; Figure S3. indicator creation flowchart; Figure S4. modelled indicators; Figure S5. finite element mesh for SPDE approximation; Figure S7–S8. uncertainty bounds for Burkina Faso, 2020; Figure S8–S10. estimates and uncertainty bounds for Burkina Faso, 2000; Figure S11–S12. uncertainty bounds for Kenya, 2020; Figure S13–S15. estimates and uncertainty bounds for Kenya, 2000; Figure S16–S17. uncertainty bounds for Nigeria, 2020; Figure S18–S20. estimates and uncertainty bounds for Nigeria, 2000; Table S1. indicator values and definitions; Table S2. surveys included in this analysis; Table S3. – surveys excluded from this analysis; Table S4. extracted survey variables; Table S5. crosswalk parameters; Table S6. MBG model parameters; Table S7. validation results; Author’s contributions. [file 12916_2023_3214_MOESM1_ESM.pdf]

Additional File 1: Mapping Heterogeneity in Family Planning Indicators in Burkina Faso, Kenya, and Nigeria, 2000–2020

Table of Contents

1 Compliance with the Guidelines for Accurate and Transparent Health Estimates Reporting (GATHER).... 3
2 Data 5
- 2.1 Survey data extraction 5
  - 2.1.1 Data processing for microdata 5
  - 2.1.2 Data processing for reports 5
- 2.2 Polygon resampling 5
- 2.3 Administrative boundaries 6
- 2.4 Gridded populations 6
- 2.5 Crosswalking 6

3 Statistical model 7
- 3.1 Covariate stacking 7
  - 3.1.1 Stacking overview 7
  - 3.1.2 Stacking sub-model 1: generalized additive model 8
  - 3.1.3 Stacking sub-model 2: ridge regression 9
  - 3.1.4 Stacking sub-model 3: boosted regression trees 9
- 3.2 Geostatistical model 10
  - 3.2.1 Model description 10
  - 3.2.2 Model fitting and prediction 12
  - 3.2.3 Model validation 13
- 3.3 Post-estimation 14
  - 3.3.1 Aggregation to administrative areas 14
  - 3.3.2 Point estimates and uncertainty intervals 14

Supplementary Figures 15
- Figure S1: Analytic process overview 15
- Figure S2: Data availability 16
- Figure S3: Indicator creation flowchart 17
- Figure S4: Modelled indicators 18
- Figure S5: Finite element mesh for SPDE approximation 19
- Figure S6: Lower bound of 95% uncertainty interval for estimated family planning indicators in Burkina Faso, 2020 20
- Figure S7: Upper bound of 95% uncertainty interval for estimated family planning indicators in Burkina Faso, 2020 21
- Figure S8: Estimated family planning indicators in Burkina Faso, 2000 22

Figure S9: Lower bound of 95% uncertainty interval for estimated family planning indicators in Burkina Faso, 2000 ... 23
Figure S10: Upper bound of 95% uncertainty interval for estimated family planning indicators in Burkina Faso, 2000 ... 24
Figure S11: Lower bound of 95% uncertainty interval for estimated family planning indicators in Kenya, 2020 ... 25
Figure S12: Upper bound of 95% uncertainty interval for estimated family planning indicators in Kenya, 2020 ... 26
Figure S13: Estimated family planning indicators in Kenya, 2000 ... 27
Figure S14: Lower bound of 95% uncertainty interval for estimated family planning indicators in Kenya, 2000 ... 28
Figure S15: Upper bound of 95% uncertainty interval for estimated family planning indicators in Kenya, 2000 ... 29
Figure S16: Lower bound of 95% uncertainty interval for estimated family planning indicators in Nigeria, 2020 ... 30
Figure S17: Upper bound of 95% uncertainty interval for estimated family planning indicators in Nigeria, 2020 ... 31
Figure S18: Estimated family planning indicators in Nigeria, 2000 ... 32
Figure S19: Lower bound of 95% uncertainty interval for estimated family planning indicators in Nigeria, 2000 ... 33
Figure S20: Upper bound of 95% uncertainty interval for estimated family planning indicators in Nigeria, 2000 ... 34

Supplementary Tables ... 35

Table S1: Family planning indicator values and differences in definitions ... 35
Table S2: Surveys included in this analysis ... 61
Table S3: Surveys excluded from this analysis ... 81
Table S4: Extracted survey variables ... 88
Table S5: Crosswalk parameters ... 92
Table S6: MBG model parameters ... 95
Table S7: Model validation results ... 97

Authors' Contributions ... 98

1 Compliance with the Guidelines for Accurate and Transparent Health Estimates Reporting (GATHER)

Item #	Checklist item	Description of Compliance
**Objectives and funding**		
1	Define the indicator(s), populations (including age, sex, and geographical entities), and time period(s) for which estimates were made.	Background and Methods section
2	List the funding sources for the work.	Funding section
**Data Inputs**		
**For all data inputs from multiple sources that are synthesised as part of the study:**		
3	Describe how the data were identified and how the data were accessed.	Methods section<br>Additional File 1: Section 2<br>Additional File 1: Table S2
4	Specify the inclusion and exclusion criteria. Identify all ad-hoc exclusions.	Methods section<br>Additional File 1: Section 2<br>Additional File 1: Tables S2–S3
5	Provide information on all included data sources and their main characteristics. For each data source used, report reference information or contact name/institution, population represented, data collection method, year(s) of data collection, sex and age range, diagnostic criteria or measurement method, and sample size, as relevant.	Additional File 1: Section 2<br>Additional File 1: Table S2
6	Identify and describe any categories of input data that have potentially important biases (eg, based on characteristics listed in item 5).	Discussion section
**For data inputs that contribute to the analysis but were not synthesised as part of the study:**		
7	Describe and give sources for any other data inputs.	N/A
**For all data inputs:**		
8	Provide all data inputs in a file format from which data can be efficiently extracted (eg, a spreadsheet rather than a PDF), including all relevant meta-data listed in item 5. For any data inputs that cannot be shared because of ethical or legal reasons, such as third-party ownership, provide a contact name or the name of the institution that retains the right to the data.	Available through GHDx
**Data analysis**		
9	Provide a conceptual overview of the data analysis method. A diagram may be helpful.	Additional File 1: Figure S1
10	Provide a detailed description of all steps of the analysis, including mathematical formulae. This description should cover, as relevant, data cleaning, data pre-processing, data adjustments and weighting of data sources, and mathematical or statistical model(s).	Methods section<br>Additional File 1: Sections 2 and 3

11	Describe how candidate models were evaluated and how the final model(s) were selected.	Additional File 1: Section 3
12	Provide the results of an evaluation of model performance, if done, as well as the results of any relevant sensitivity analysis.	Additional File 1: Section 3.2.3
13	Describe methods for calculating uncertainty of the estimates. State which sources of uncertainty were, and were not, accounted for in the uncertainty analysis.	Methods section<br>Additional File 1: Section 3
14	State how analytic or statistical source code used to generate estimates can be accessed.	Available through GitHub
Results and Discussion		
15	Provide published estimates in a file format from which data can be efficiently extracted.	Available through GHDx
16	Report a quantitative measure of the uncertainty of the estimates (eg, uncertainty intervals).	Results section<br>Additional File 1: Figures S6–7, S9–12, S14–17, S19–20
17	Interpret results in light of existing evidence. If updating a previous set of estimates, describe the reasons for changes in estimates.	Discussion section
18	Discuss limitations of the estimates. Include a discussion of any modelling assumptions or data limitations that affect interpretation of the estimates.	Discussion section

Checklist template obtained from: http://gather-statement.org/

2 Data

2.1 Survey data extraction

2.1.1 Data processing for microdata

To prepare survey microdata for analysis, we first subset the data to females ages 15 to 49 years and then dropped any observations missing a survey weight (0.5% of all observations). Coordinates outside of but within 15 km of the country border were snapped to be approximately 200m inside the nearest border of the specified country (0.3% of all observations).

We then aggregated the individual-level microdata to calculate four values at the finest possible spatial resolution: among all women, the proportion who are using contraceptives ($p^{anycontra}$); among women who are using contraceptives, the proportion who are using modern methods ($p^{modcontra}$); among women who are not using contraceptives, the proportion who are in need of family planning (p^{need}); and among women who are not using contraceptives and are in need of family planning, the proportion who express an intent to use contraceptives in the future (p^{intent}). Ideally, this calculation was carried out for each latitude and longitude pair representing the location of the survey cluster (point-level data). Where cluster-level GPS coordinates were not available, we geo-located survey microdata to the smallest geographical area (polygon) possible. Individual-level sample weights were used when calculating prevalence, and the effective sample size for each prevalence estimate was estimated via the Kish approximation [23], which accounts for differences in the underlying selection probability within a sample. The interview date for each specific location was calculated as the median of the individual-level interview dates.

2.1.2 Data processing for reports

In situations where individual-level microdata were not available, we utilized summary reports. We used the median month of the reported data collection periods (ranging from 1 to 12 months in length) as the interview dates to align with the extracted microdata. We adjusted reported sample sizes by multiplying by the median design effect (ratio of effective sample size to observed sample size) calculated in the microdata as described above.

2.2 Polygon resampling

Wherever possible, we matched prevalence data to a specific latitude and longitude. In instances where this was not possible, we matched data to the smallest area (termed a polygon). In most instances, these polygons represented administrative areas. The statistical model we employed requires point-referenced data, so data matched to polygons were resampled to generate pseudo-point data based on the underlying population distribution within the polygon. The methods for this resampling are consistent with those previously used in the geospatial modelling of other health indicators [25–29].

In this framework, we randomly sampled 10,000 locations among grid cells for each polygon-level observation with probability proportional to the grid cell population. We considered grid cells to be contained within a polygon if their centroid fell within the geographical boundary. We then performed k-means clustering (with k set to 1 per 40 grid cells) on the sampled points to generate a reduced set of locations based on the k-means cluster centroids to be used in modelling. We assigned weights to each pseudo-point proportional to the number of sampled points contained in each of the k-means clusters, which translated to the number of sampled points divided by 10,000. We assigned each pseudo-point generated by this process the prevalence observed for the polygon as a whole, and a sample size equal to the sample size for the polygon as a whole multiplied by the weight derived for each point.

2.3 Administrative boundaries

For this analysis we used the Database of Global Administrative Areas (GADM) version 3.6 shapefiles [30] to define boundaries for countries and their first- and second-level administrative areas. Minor adjustments to these shapefiles were made where names were missing in the original shapefiles or where more accurate boundary information was identified from other sources. Specifically, in Kenya, seven unnamed locations in the second administrative level shape files were dissolved into their neighbours, while in Nigeria, one location that was entirely in Lake Chad was removed. No changes were made for Burkina Faso.

2.4 Gridded populations

The gridded population data used for this analysis were obtained from WorldPop [31, 32]. Because WorldPop provides data at a 100x100-m spatial resolution and for five year age groups, we first aggregated the raster by taking the sum of the finer rasters to produce estimates of the population at a 5x5-km resolution for women ages 15 to 49. In a small number of locations and in three years (2000, 2001, and 2012) there were inconsistencies between in the No Data mask compared to other years for the same location. To address this for 2000 and 2001, we extrapolated based on the 2003–2005 data years, and for 2012, we interpolated based on 2011 and 2013.

2.5 Crosswalking

In some instances, prevalence estimates were calculated without using all expected variables or only including women in a union, which we denote below as alt, where $alt \neq ref$ (alt = “alternate”, ref = “reference”). In these instances, we used the CrossWalk package in R [33] to predict adjustment factors for these systematically biased estimates of prevalence.

To do this, we identified surveys that collected all expected variables and we produced prevalence estimates at the first administrative level for two scenarios: using all expected variables (ref) and excluding the combination of variables for each observed alternate option (alt). We incorporated three dummy variables as covariates, one for each country included in the dataset, and omitted the intercept. Then, for each alternate scenario, we used these data to fit the following logit-difference meta-regression model for each indicator:

$$\text{logit}\left(p_{i,j}^{alt}\right) - \text{logit}\left(p_{i,j}^{ref}\right) = \beta_{BFA} \cdot I_{BFA,i} + \beta_{KEN} \cdot I_{KEN,i} + \beta_{NGA} \cdot I_{NGA,i} + u_j + \epsilon_i$$

where:

- $p_{i,j}^{ref}$ is the observed indicator calculated for observation i for study j from all expected variables for all women (reference);
- $p_{i,j}^{alt}$ is the observed indicator calculated for observation i for study j, excluding variables missing from the survey and/or including only women in a union (alternate);
- β_{BFA}, β_{KEN}, β_{NGA} are regression coefficients for Burkina Faso, Kenya, and Nigeria, respectively, representing the average logit-difference between the alternate and reference prevalence values in these three countries;
- $I_{BFA,i}$, $I_{KEN,i}$, $I_{NGA,i}$ are indicator variables which are 1 if observation i is from Burkina Faso, Kenya, or Nigeria, respectively, and 0 otherwise;
- u_j is a normally-distributed source-specific random effect with variance γ, representing between-study heterogeneity in the adjustment factor;

- and ϵ_i is a normally-distributed error term.

In this model, a logit transform was applied to ensure the adjusted prevalence was restricted to between 0 and 1. For values where the unadjusted prevalence was equal to 0 and 1, a transformation was employed to enable applying the logit transformation: for 0 values, ε_0 was calculated as half the smallest observed non-zero value, and all instances of 0 were replaced with ε_0; for 1 values, ε_1 was calculated as half of the largest observed non-one values subtracted from one, and all instances of 1 were replaced with (1-ε_1). For each crosswalk, the fitted model coefficients and the variance of the source-specific random effect prediction adjustment factors (β) for each country and between-study heterogeneity variance (γ) are listed in Supplementary Table S5.

For each observation from a survey that did not include all expected variables and/or limited relevant questions to only women in union, corrected prevalence estimates were calculated as:

$$p^{adjusted} = \text{logit}^{-1}\left(\text{logit}\left(p^{observed}\right) - \beta_{country}\right)$$

using the β value from the appropriate model (corresponding to the specific alternate question set used) and country. Standard errors for $\text{logit}(p^{adjusted})$ were calculated as:

$$\text{SE}\left(\text{logit}(p^{adjusted})\right) = \sqrt{\text{SE}\left(\text{logit}(p^{observed})\right)^2 + \text{SE}\left(\beta_{country}\right)^2 + \gamma}$$

Standard errors for $p^{adjusted}$ were then calculated from $\text{SE}\left(\text{logit}(p^{adjusted})\right)$ using a delta transformation. Finally, we calculated new effective sample sizes for all adjusted data points leveraging the relationship between variance and sample size of a binomial distribution:

$$N^{eff} = \frac{p^{adjusted} \cdot (1 - p^{adjusted})}{\left(SE(p^{adjusted})\right)^2}$$

This new effective sample size is a function of the original sample size, adjusted prevalence, and the uncertainty from the meta-regression crosswalking model. Surveys where crosswalking was required are identified in Supplementary Table S2.

3 Statistical model

The statistical modelling approach, and the technical description below, are similar to previous analyses of under-5 mortality [25], educational attainment [26], child growth failure [27], vaccine coverage [28], and HIV prevalence [29], among others.

3.1 Covariate stacking

3.1.1 Stacking overview

Stacked generalization/regression ("stacking") is an ensemble modelling method that combines multiple prediction methods to increase predictive validity over that achieved using a single modelling approach. This method relies on several sub-models that are fit independently and then combined by a secondary model [34]. Our implementation of stacking largely follows the approach described by Bhatt and colleagues [35].

For this analysis, we fit three sub-models: generalized additive models, ridge regression, and boosted regression trees to the family planning data described above and five covariates: night time lights [36, 37], travel time to nearest settlement [38, 39], travel time to health care facilities [40, 41], population [31, 32], and mean years of education among women ages 15–49 [42, 43]. These five covariates were selected opportunistically from among variables available with the appropriate spatial resolution and coverage. Some variables that we expect would be predictive—for example, socioeconomic status, religion, women's autonomy—were not available at a local spatial resolution and were thus not included. We selected the three sub-models based on their ease of implementation through existing software packages, the fundamental differences in the approach and thus differences in strengths and weaknesses, and a proven track record of predictive accuracy [35]. Further details on the implementation of each sub-model are provided below.

Each sub-model was fit using five-fold cross-validation to avoid overfitting. For each sub-model, we produced both out-of-sample and in-sample predictions. Out-of-sample predictions were generated by compiling the predictions from the five holdouts across each cross-validation fold, and in-sample predictions were generated by re-fitting the sub-models using all available data. The out-of-sample sub-model predictions were used as explanatory covariates when fitting the geostatistical model described below, and the in-sample predictions were used when generating predictions from this same geostatistical model in order to maximize data use. In both instances, the logit-transformation of the predictions was used to put these predictions on the same scale as the linear predictor in the geostatistical model.

3.1.2 Stacking sub-model 1: generalized additive model

Generalized additive models (GAMs) are generalized linear models where the linear predictor is formed via the sum of smooth functions of the covariates [44]. In the context of stacking, the primary advantage of the GAM is the flexibility to estimate potentially complex non-linear relationships between the outcome and each of the covariates. For the purposes of this analysis, we fitted a GAM with a binomial likelihood, a logit link for the linear predictor, and thin-plate regression splines as the smoothing functions. As an example, the GAM for the proportion of women using any contraceptives ($p^{anycontra}$) is given by:

$$Y_{i,t,j} \sim \text{Binomial}\left(p_{i,t,j}^{anycontra}, N_{i,t,j}\right)$$
$$\text{logit}\left(p_{i,t,j}^{anycontra}\right) = \beta + f_1\left(X_{1,i,t}\right) + f_2\left(X_{2,i,t}\right) + f_3\left(X_{3,i,t}\right) + f_4\left(X_{4,i,t}\right) + f_5\left(X_{5,i,t}\right)$$

where:

- $N_{i,t,j}$ and $Y_{i,t,j}$ are the number of women aged 15–49 years sampled and the number of women who report using any contraceptives among those sampled, respectively, in location i and year t for survey j;
- $p_{i,t,j}^{anycontra}$ is the underlying prevalence of contraceptive use in location i and year t as measured in survey j;
- β is an intercept;
- $X_{1,i,t}, \ldots, X_{5,i,t}$ are the values of each of the five covariates in location i and year t;
- $f_1(\,), \ldots, f_5(\,)$ are the smoothing functions (thin-plate regression splines) corresponding to the five covariates.

We fitted the GAMs using the mgcv package in R [45, 46]. A modified version of the thin-plate regression spline (`s(…, bs="ts")`) was used which includes a modification to the smoothing penalty that allows the entire term to be shrunk to zero, effectively incorporating covariate selection into the model-fitting process.

3.1.3 Stacking sub-model 2: ridge regression

Ridge regression is an approach to fitting a linear regression model (or, in this case, a generalized linear regression model) that shrinks the regression coefficients towards zero via a penalty on the sum of the squared regression coefficients [44]. This sum of the squared regression coefficients is multiplied by a "complexity parameter," lambda, which controls the degree of shrinkage. For the purposes of this analysis, we fitted the ridge regression using a binomial likelihood and a logit link function. As an example, the ridge regression model for the proportion of women using any contraceptives ($p^{anycontra}$) is given by:

$$Y_{i,t,j} \sim \text{Binomial}\left(p_{i,t,j}^{anycontra}, N_{i,t,j}\right)$$
$$\text{logit}\left(p_{i,t,j}^{anycontra}\right) = \beta_0 + \beta_1 \cdot X_{1,i,t} + \beta_2 \cdot X_{2,i,t} + \beta_3 \cdot X_{3,i,t} + \beta_4 \cdot X_{4,i,t} + \beta_5 \cdot X_{5,i,t}$$

where:

- $N_{i,t,j}$ and $Y_{i,t,j}$ are the number of women aged 15–49 years sampled and the number of women who report using any contraceptives among those sampled, respectively, in location i and year t for survey j;
- $p_{i,t,j}^{anycontra}$ is the underlying prevalence of contraceptive use in location i and year t as measured in survey j;
- β_0 is an intercept;
- $X_{1,i,t}, \dots, X_{5,i,t}$ are the values of each of the five covariates in location i and year t;
- $\beta_1, \dots, \beta_5$ are regression parameters corresponding to the five covariates.

We fitted the ridge regression models using the glmnet package in R [47]. Ten-fold cross-validation was used to select the lambda parameter.

3.1.4 Stacking sub-model 3: boosted regression trees

Boosted regression trees (BRT) is a machine learning technique that combines regression tree models with boosting [48]. In the context of stacking, the primary advantage of BRT is the flexibility to estimate potentially complex interactions between the various covariates when predicting the outcome. Regression tree models work by partitioning a dataset via a sequence of recursive binary splits based on the values of different covariates in the model; within the regions defined by these splits, a simple regression with only a constant is fit. Boosting is an algorithm whereby models (in this case, regression tree models) are fitted sequentially, with a focus on improving prediction in subsequent steps in areas where prediction was initially poor. In BRT, the first set of regression tree models fit is to predict the outcome of interest; all subsequent models are fitted using the residual from the previous model as the outcome. Final predictions are then constructed as a weighted sum of the predictions from each individual model in the sequence. BRT is controlled by four parameters: the learning rate, which determines the contribution of each regression tree model to the overall prediction (ie, the weighting of the different models); the tree complexity, which determines the number of binary splits in each tree; the number of trees, which controls how many regression tree models are fitted; and the bag fraction, which controls the proportion of the data that are (randomly) included when fitting each regression tree model.

We fitted the BRT models using the dismo package in R [49], with the learning rate set to 0.005, the tree complexity set to 3, and the bagging fraction set to 0.75. Cross-validation was used to select the number of trees for the final model.

3.2 Geostatistical model

3.2.1 Model description

We modelled the proportion of women using any contraceptives ($p^{anycontra}$) using the following generalized linear mixed effects model:

$$Y_{i,t,j} \sim \text{Binomial}\left(p_{i,t,j}^{anycontra}, N_{i,t,j}\right)$$
$$\text{logit}\left(p_{i,t,j}^{anycontra}\right) = \beta_0 + \boldsymbol{\beta}_1 \boldsymbol{X}_{i,t} + Z_{1,i} + Z_{2,k[i],t} + \epsilon_{1,k[i],j} + \epsilon_{2,i,t}$$

$$Z_{1,i} \sim \text{GP}(0, \Sigma_{space})$$
$$Z_{2,k[i],t} \sim \text{RW2}\left(\sigma_{time}^2\right)$$
$$\epsilon_{1,k[i],j} \sim \text{Normal}(0, \sigma_{survey}^2)$$
$$\epsilon_{2,i,t} \sim \text{Normal}\left(0, \sigma_{nugget}^2\right)$$

where:

- $N_{i,t,j}$ and $Y_{i,t,j}$ are the number of women aged 15–49 years sampled and the number of women who report using any contraceptives among those sampled, respectively, in location i and year t for survey j;
- $p_{i,t,j}^{anycontra}$ is the underlying prevalence of contraceptive use in location i and year t as measured in survey j;
- β_0 is an intercept;
- $\boldsymbol{X}_{i,t}$ is a vector of logit-transformed stacked covariates for location i and year t, and $\boldsymbol{\beta}_1$ is the corresponding vector of regression coefficients;
- $Z_{1,i}$ is a spatially correlated random effect for location i;
- $Z_{2,k[i],t}$ is a temporally correlated random effect for year t in the first-level administrative area k containing location i;
- $\epsilon_{1,k[i],j}$ is an independent and identically distributed random effect for survey j in first-level administrative area k;
- and $\epsilon_{2,i,t}$ is an independent and identically distributed random effect for location i and year t.

Descriptively, logit-transformed contraceptive prevalence is given in this model as a linear combination of an intercept (β_0), covariate effects ($\boldsymbol{\beta}_1 \boldsymbol{X}_{i,t}$), a spatially correlated random effect ($Z_{1,i}$), a temporally correlated random effect for each first-level administrative area ($Z_{2,k[i],t}$), an uncorrelated survey-specific error term ($\epsilon_{1,k[i],j}$), and an uncorrelated location- and time-specific error term or nugget effect ($\epsilon_{i,t}$). The intercept is intended to capture the mean level of contraceptive prevalence, while the covariate effects capture spatial and temporal variation in contraceptive prevalence that can be described as a function of spatial and temporal variation in the included covariates. The spatially correlated random effect is intended to capture additional spatial variation in contraceptive prevalence, while the temporally correlated random effect is intended to capture additional temporal variation in contraceptive prevalence and is allowed to vary by first-level administrative area. We also include two error terms, one by survey and one by location and time period. The survey-specific error term is intended to capture differences in

prevalence as measured by different surveys, for example due to differences in question wording that are not captured by our crosswalking process, or due to other differences in the way the survey was conducted or prepared for analysis. Importantly, we assume these differences do not reflect actual differences in the underlying prevalence of contraceptive use, but rather are artifacts of how the data were collected. The location and time period error term, or nugget effect, is intended to capture any additional, non-structured variation in contraceptive prevalence that is not accounted for in other components of the model. This variation is viewed as reflecting true variation in the underlying prevalence of contraceptive use, but as this variation is not captured by the covariate effects or the correlated random effects, we are only able to estimate this effect in locations and time periods with observed data.

Priors for the intercept and covariate effects were specified as:

$$\beta_0 \sim \text{Normal}(0, 1)$$
$$\beta_{1,model} \sim \text{Normal}(0, 1000)$$

Additionally, the elements of $\boldsymbol{\beta_1}$ were constrained to sum to one.

The spatially correlated random effect ($Z_{1,i}$) was modelled as a Gaussian process with mean 0 and covariance matrix given by a Matérn covariance function [50]:

$$\Sigma_{space} = \sigma_{space}^2 \frac{2^{1-\nu}}{\Gamma(\nu)} \times (\kappa \boldsymbol{D})^{\nu} \times \mathrm{K}_{\nu}(\kappa \boldsymbol{D})$$

For this analysis, the smoothness parameter (ν) was fixed at 1, and a penalized complexity (PC) prior was specified for the spatial range (ρ_{space}, where $\rho_{space} = \sqrt{8\nu}/\kappa$ and is equal to the distance at which correlation is approximately 0.1) and the marginal standard deviation (σ_{space}). PC priors shrink towards a more simplistic base model, in this case, one where the marginal variance is 0 and the spatial range is infinite, and are specified via setting the tail probabilities on each hyper-parameter [51, 52]. We followed the guidance provided by Fuglstad et al. [53], who recommend selecting priors that satisfy $\Pr(\sigma_{space} > \sigma_{space_0}) = 0.05$ and $\Pr(\rho_{\text{space}} < \rho_{space_0}) = 0.05$, where σ_{space_0} is between 2.5 to 40 times the expected true marginal standard deviation and ρ_{space_0} is between $1/10$ to $1/2.5$ of the expected true range. In this case, we set $\sigma_{space_0} = 3$ and set ρ_{space_0} to 5% the maximum extent of the mesh used for the SPDE approximation (described below), or 0.509 in Burkina Faso, 0.600 in Kenya, and 0.717 in Nigeria. The temporally correlated random effect ($Z_{2,k[i],t}$) was modelled as a second-order random walk, with separate random walks fitted for each first-level administrative area (this corresponds to a “Type II” space-time interaction, as described by Knorr-Held [54], ie, correlated temporally but not spatially). Both $\epsilon_{1,j}$ and $\epsilon_{2,i,t}$ were assumed to follow independently and identically distributed (IID) Normal distributions. PC priors were used for the standard deviation of $Z_{2,k[i],t}$, $\epsilon_{1,j}$, and $\epsilon_{2,i,t}$, and were set to:

$$\sigma_{time_0} = 3;\ \Pr(\sigma_{time} > \sigma_{time_0}) = 0.05$$
$$\sigma_{survey_0} = 3;\ \Pr(\sigma_{survey} > \sigma_{survey_0}) = 0.05$$
$$\sigma_{nugget_0} = 3;\ \Pr(\sigma_{nugget} > \sigma_{nugget_0}) = 0.05$$

Similar models were specified for $p^{modcontra}$, p^{need}, and p^{intent}. When modelling $p^{modcontra}$, $N_{i,t,j}$ corresponds to the number of sampled women who are using any contraception, while $Y_{i,t,j}$ corresponds to the number of women sampled who are using modern methods of contraception. When modelling p^{need}, $N_{i,t,j}$ corresponds to the number of sampled women who are not using any contraception, while $Y_{i,t,j}$ corresponds to the number of women sampled who are not using any contraception and are in need of family planning. When modelling p^{intent}, $N_{i,t,j}$ corresponds to the number of sampled women who are not using any contraception and are in need of family planning, while $Y_{i,t,j}$ corresponds to the number of women sampled who are not using any contraception and are in need of family planning and also express an intention to use contraception in the future. All other model parameters were defined as described above.

For each indicator, separate models were fitted for each country. Models were fit separately by country rather than together to allow for country differences in the relationship between each indicator and the covariates, as well as the degree of spatial and temporal correlation. This is also a practical decision, as Burkina Faso, Kenya, and Nigeria do not share borders.

In two cases—the models for $p^{modcontra}$ and p^{intent} in Kenya—the model described above proved difficult to fit, we suspect due to data sparsity in some regions. Consequently, in these two cases only, we fit a simplified version of the model above where $Z_{2,k[i],t}$ was reduced to $Z_{2,t}$, ie, there was one single second-order random walk fitted for the whole country, rather than separate random walks for each first-level administrative area.

3.2.2 Model fitting and prediction

The models were fit in R-INLA, which relies on analytic approximations to the posterior distribution of the model parameters based on the Laplace approximation, and is an alternative to Markov chain Monte Carlo (MCMC) methods, which can be prohibitively computationally expensive for complex spatial models [55]. A stochastic partial differential equations (SPDE) approach [56] was used to approximate the continuous spatial Gaussian random field ($Z_{1,i}$). We constructed a finite elements mesh for the SPDE approximation to the Gaussian process regression using a simplified polygon boundary (Figure S5). We set the mesh triangle maximum edge length in the inside modelling domain to be 0.3 decimal degrees, and the maximum triangle edge length in the mesh outside modelling domain to be 1 decimal degrees. The fitted model parameters are listed in Table S6.

After fitting each model, we generated 1,000 draws of all model parameters from the approximated joint posterior distribution using the inla.posterior.sample() function in R-INLA. For each draw s of the model parameters we constructed a draw of the prevalence as:

$$p_{i,t}^{(s)} = \text{logit}^{-1}\left(\beta_0^{(s)} + \boldsymbol{\beta}_1^{(s)}\boldsymbol{X}_{i,t} + Z_{1,i}^{(s)} + Z_{2,k[i],t}^{(s)} + \epsilon_{2,i,t}^{(s)}\right)$$

Additional processing of the output from inla.posterior.sample() was required for the spatial random effect ($Z_{1,i}^{(s)}$) and the nugget effect ($\epsilon_{2,i,t}^{(s)}$) prior to constructing $p_{i,t}^{(s)}$ according to the equation above. Specifically, for $Z_{1,i}^{(s)}$, draws are generated initially only at vertices of the finite element mesh, so we project from this mesh to each location i desired for prediction, ie, the centroid of each grid cell on an approximately 5x5-km grid. For the nugget effect, we generate $\epsilon_{2,i,t}^{(s)}$ for each combination of (i,t) by sampling from $\text{Normal}\left(0, {\sigma_{nugget}^2}^{(s)}\right)$. The survey random effect ($\epsilon_{1,k[i],j}$) was not included when

generating draws of prevalence, as this random effect is assumed to represent survey-specific biases rather than true variation in prevalence. At the end of this process, we had 1,000 draws of $p_{i,t}$ for each grid cell and year combination.

3.2.3 Model validation

We used five-fold cross validation to assess the performance of the model described above for each of the four modelled indicators. This involved randomly splitting the survey data into five groups ("folds") with roughly equal total sample size; fitting the model five times, excluding each fold in turn; and then matching the data withheld from each model to the predictions from that model for the corresponding locations and time-periods. This procedure resulted in a complete dataset of out-of-sample predictions corresponding to all survey data observations included in the analysis. We used this dataset to compute three measures of model performance: the mean error (a measure of bias), the coverage of the 95% prediction intervals (a measure of the calibration of the uncertainty estimates), and the root mean squared error (RMSE, a measure of total variance). In order to calculate the mean error, we first calculated the error for each row in the dataset as the difference between the out-of-sample prediction and the data, and then calculated the mean error across the entire dataset, weighted by the effective sample size. To calculate coverage, we first constructed 95% prediction intervals for each out-of-sample prediction by simulating from a binomial distribution 1,000 times with prevalence equal to the 1,000 draws of $p_{i,t}$ from the approximated posterior (as described above) and sample size equal to the effective sample size for the corresponding data observation, and then calculating the 2.5th and 97.5th percentiles of these 1,000 draws. Coverage was calculated as the percentage of the data-estimate pairs where the number of "cases" observed was within the prediction interval, weighted by the effective sample size. Because direct estimates based on a single survey cluster are typically noisy due to very small sample sizes, we elected to aggregate both the data and the corresponding out-of-sample predictions (weighted by effective sample size) within administrative areas prior to calculating error for each area, and then the RMSE across all areas. We did this three times: at the country level, at the first administrative level, and at the second administrative level. For comparison purposes, we also calculated these same summary metrics using in-sample predictions (ie, from the models fitted using all available data).

Supplemental Table S7 shows the results of this validation exercise. Across indicators and countries, and for both in-sample and out-of-sample predictions, the mean error is typically close to zero, indicating that the models produce relatively unbiased estimates. Coverage of the 95% uncertainty intervals ranged from 91.6% to 98.9% across indicators and countries for in-sample predictions, and from 90.9% to 98.1% for out-of-sample predictions. Ideally, coverage would be approximately 95% in all cases; while these results are fairly close to this target generally, these results suggest that our models tend to slightly over-estimate uncertainty in some cases (eg, when estimating modern contraceptive using in Burkina Faso) and slightly under-estimate uncertainty in other cases (eg, when estimating any contraceptive use in Nigeria). The RMSE was more variable across indicators, countries, and between in- and out-of-sample predictions. The in-sample RMSE was typically lower than the out-of-sample RMSE, indicating that the modelled estimates are more closely aligned with the data when those data were included in the model fitting process, as expected. Moreover, the gap between in-sample and out-of-sample RMSE was generally small, which suggests that the models did not over-fit the observed data. Both in-sample and out-of-sample, the RMSE was lowest when assessed at the country level, higher when assessed at the first administrative level, and highest when assessed at the second administrative level. This indicates that the modelled estimates have the smallest error at the national level, and the largest error at the most disaggregated level, which is also expected, given the larger amount of data informing the estimates (both in- and out-of-sample) at the national compared to the first administrative level, and at the first

administrative level compared to the second administrative level. This increase also likely, in part, reflects the increasing sampling variability in the data—and thus the gold standard for the purposes of calculating the RMSE—when aggregated at more granular levels. Finally, the RMSE varied considerably across countries and indicators. Examining just the out-of-sample results at the second administrative level, the RMSE was largest for intention to use contraceptive prevalence for all countries (ranging from 13.0 to 15.4 PPT across countries) and for modern contraceptive use for Nigeria (14.3) and was substantially lower (<9 PPT) for all other country-indicator pairs. It is difficult to disentangle to what extent these RMSE values reflect the actual performance of the model versus variability in the underlying data (which are an imperfect "gold standard").

3.3 Post-estimation

3.3.1 Aggregation to administrative areas

In order to generate estimates for each indicator for administrative areas, we first needed to allocate each grid cell or fraction of a grid cell to a given administrative area. In order to accomplish this preliminary step, we intersected each grid cell with the second administrative level shape file to determine what fraction of each grid cell fell within each administrative area. Since all second-level administrative areas nest within first-level administrative areas, which in turn nest within countries, this strategy assigned the cell fractions to an administrative area at each level of the administrative hierarchy. We assumed that population density within each cell was uniform, and for cells that were split across multiple areas, we allocated the WorldPop population estimate in proportion to the area of overlap. This process was carried out separately for each country, so cells that cross international borders were allocated in their entirety to the country that contained the areal majority of the grid cell.

In addition to estimates of family planning indicators on a 5x5-km grid, we constructed estimates for first- and second-level administrative areas. These estimates were derived by calculating population-weighted averages of the indicator values across each entire grid cell or fraction of a grid cell assigned to a given administrative area. The population used to calculate these weighted averages was matched to the denominator of each indicator. For variables where the denominator is all women ages 15–49 years (CPR, mCPR, tCPR, and unmet need), this was simply the WorldPop-derived population estimates described above. For met need, the population of women in need of family planning was calculated by multiplying the WorldPop population estimate by the estimated proportion of women in need (ie, $1 - p^A$). For intention to use, the population of women not currently using any method of contraception and in need of family planning was calculated by multiplying the WordPop population estimate by the estimated proportion of women not currently using any method of contraception and in need of family planning (ie, $p^B + p^C$). In all cases, this process was carried out for each of the 1,000 posterior draws at the grid-cell level, resulting in 1,000 posterior draws at each administrative level.

3.3.2 Point estimates and uncertainty intervals

Point estimates for each grid cell and each administrative area were calculated as the mean of the 1,000 posterior draws, and lower and upper uncertainty bounds were calculated as the 2.5th and 97.5th percentile of these draws.

Supplementary Figures

Figure S1: Analytic process overview

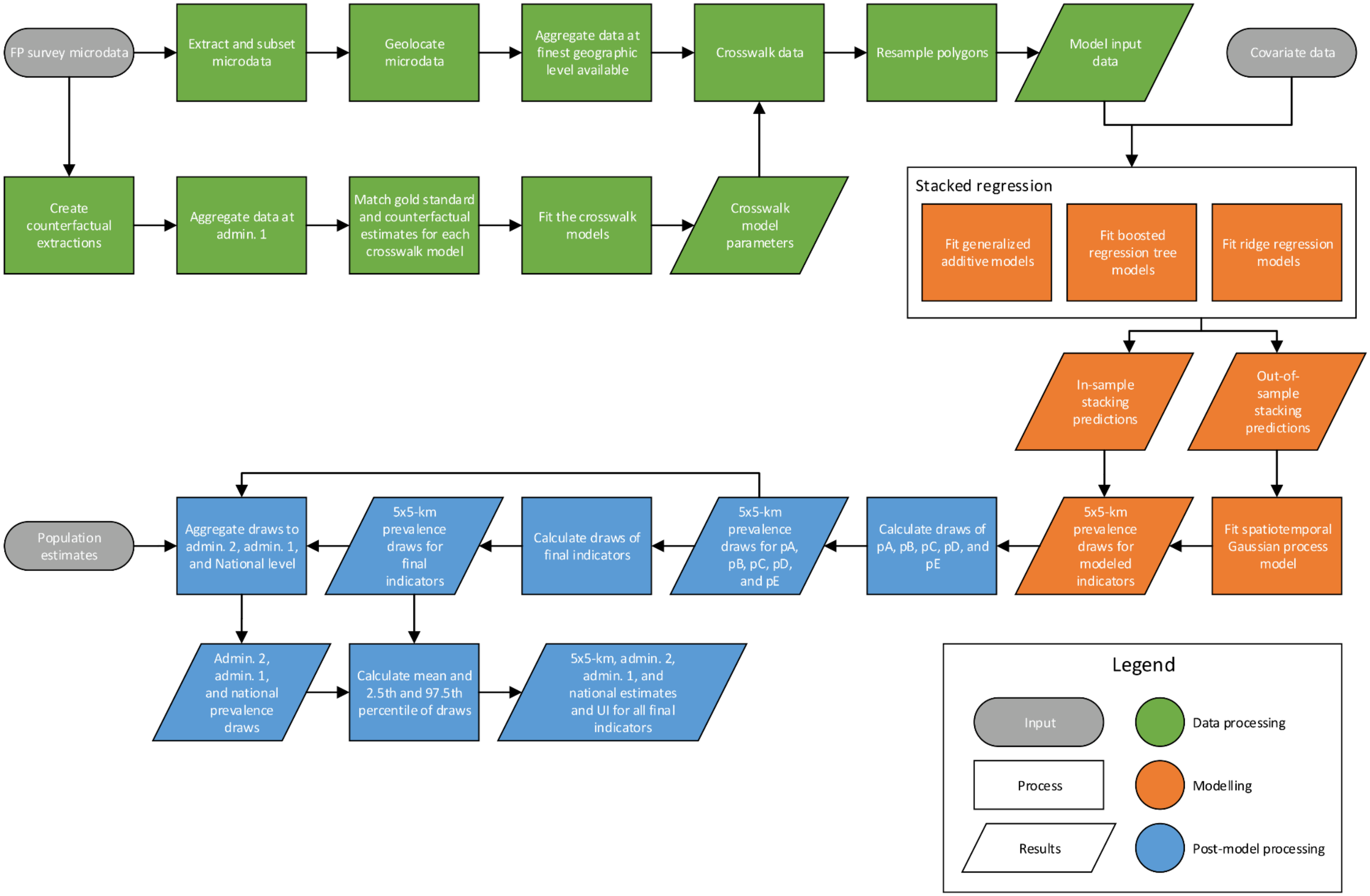

Figure S2: Data availability

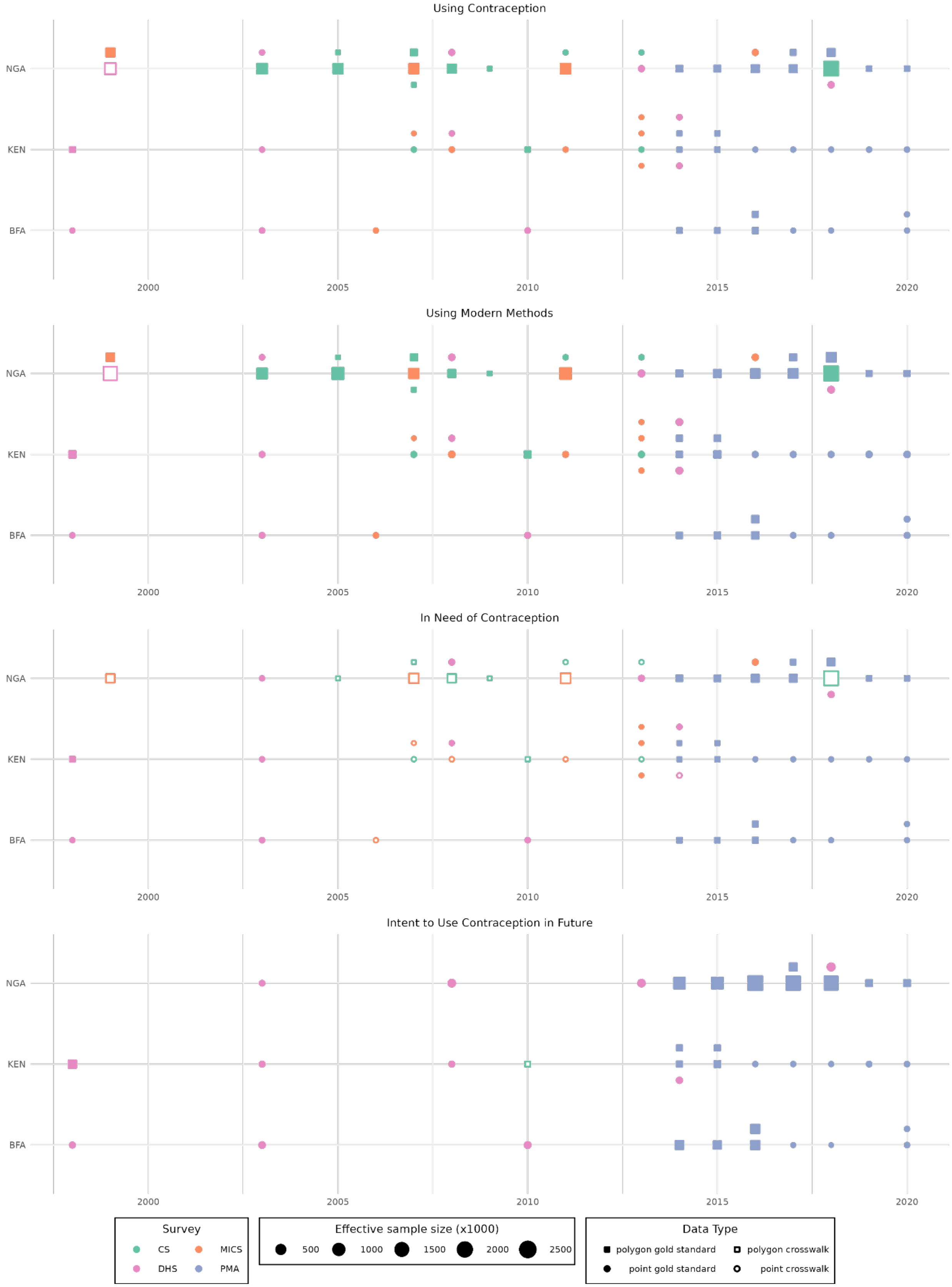

Figure S3: Indicator creation flowchart

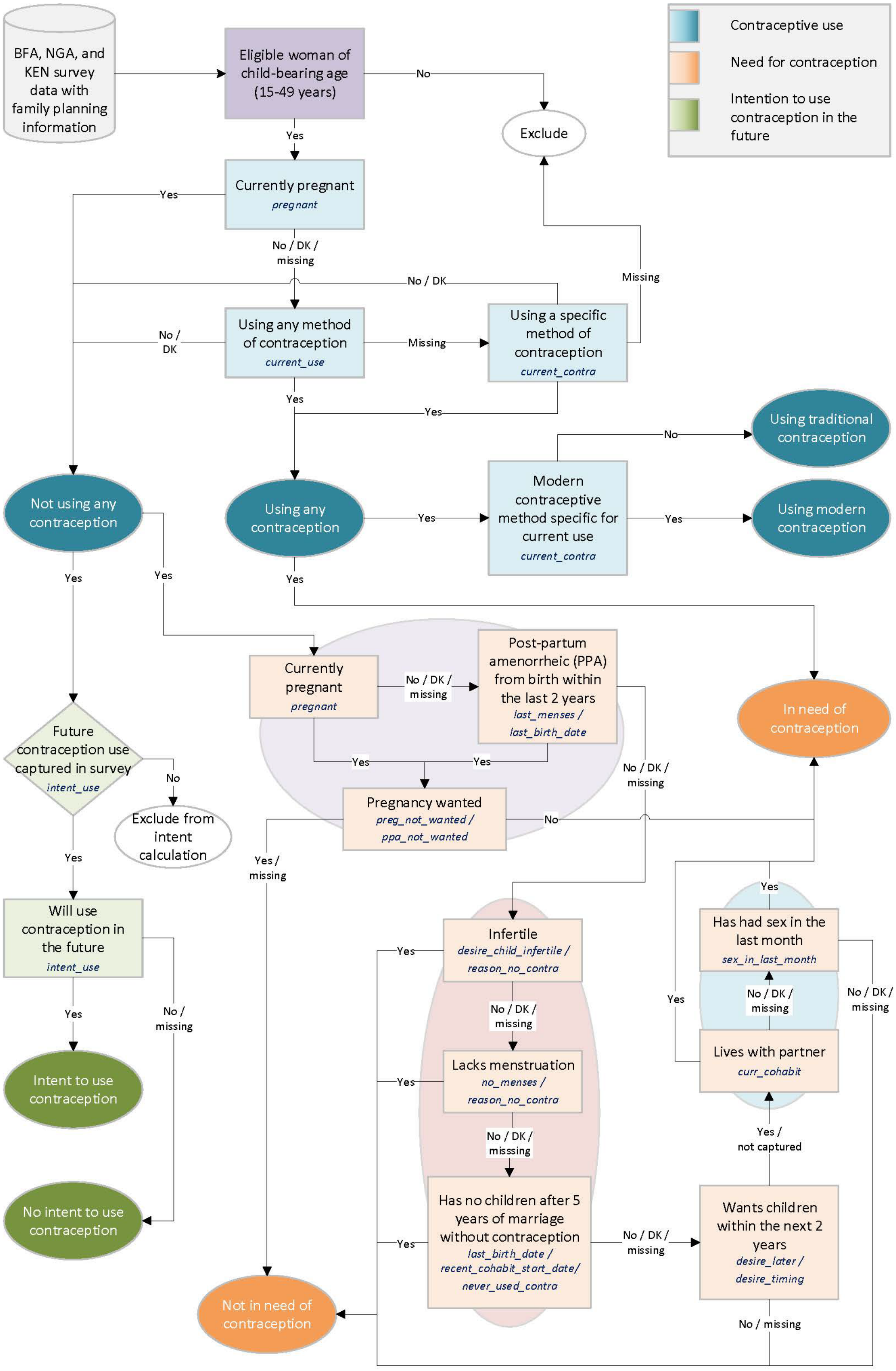

Figure S4: Modelled indicators

(1) Is the woman using contraception?	Not using contraception			Using contraception	
(2) If she is not using contraception, is she in need?	Not in need	In need		↓	
(3) If she is using contraception, is she using a traditional or a modern method?	↓	↓		Using traditional methods	Using modern methods
(4) If she is not using contraception and is in need, does she intend to use contraception in the future?	↓	No intent to use	Intent to use	↓	↓
	**Group A**: Women not in need	**Group B**: Women in need, not using, no intent to use	**Group C**: Women in need, not using, intend to use	**Group D**: Women in need, using traditional methods	**Group E**: Women in need, using modern methods

Each of the first four rows corresponds to a separate geostatistical model, and the final row corresponds to the five mutually exclusive and collectively exhaustive groups that are constructed based on combination of the four modelled indicators.

Figure S5: Finite element mesh for SPDE approximation

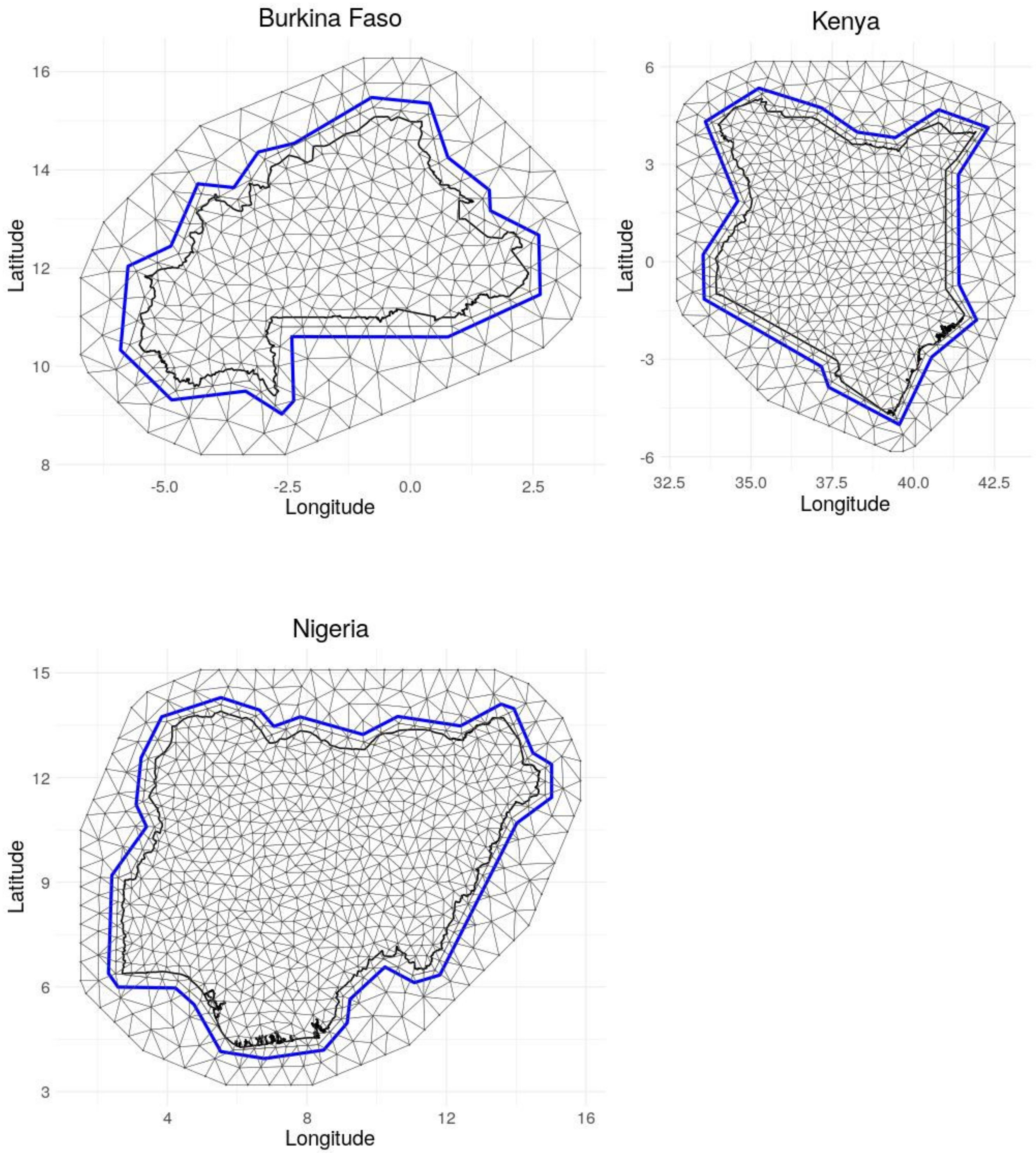

Figure S6: Lower bound of 95% uncertainty interval for estimated family planning indicators in Burkina Faso, 2020

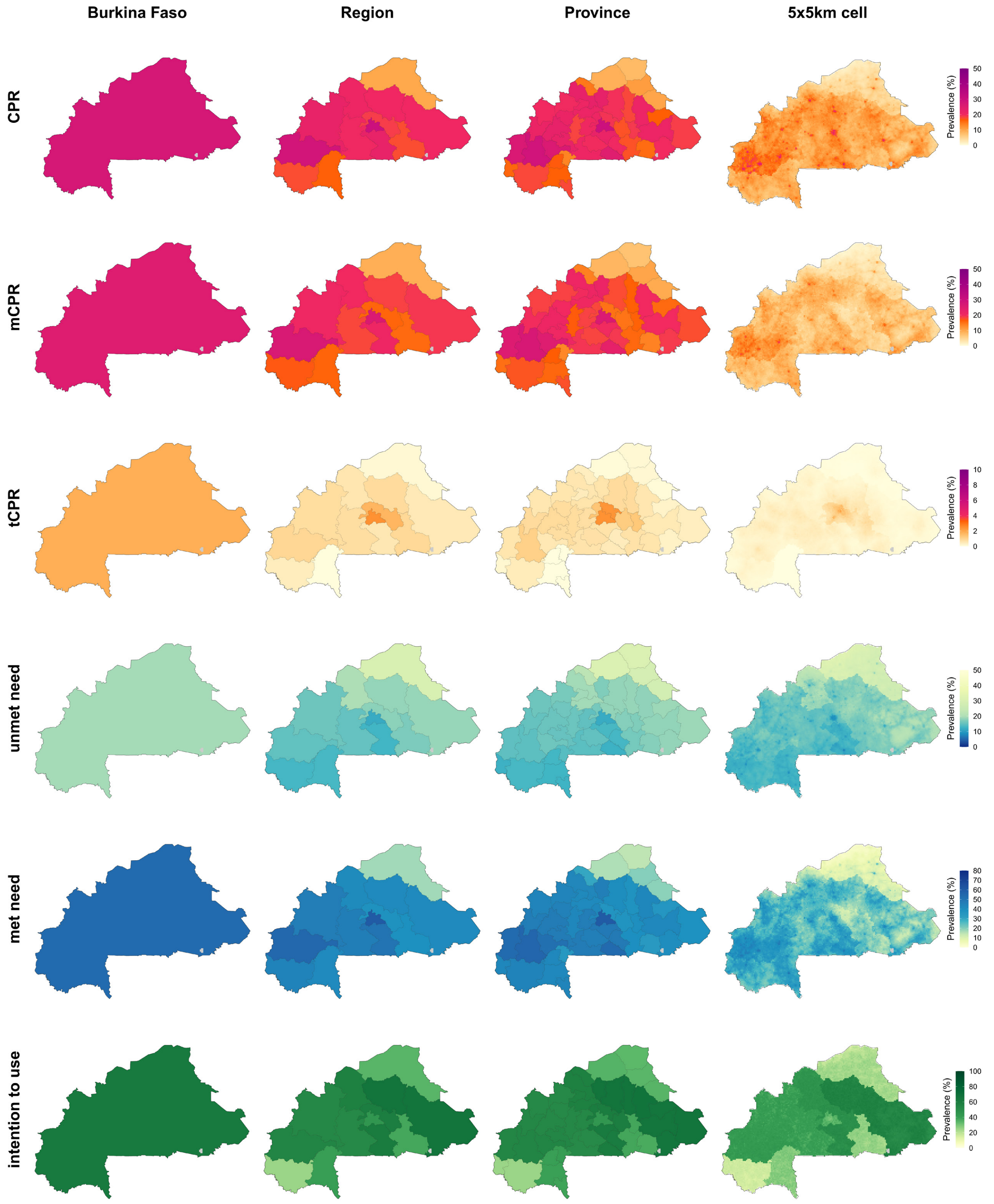

Note: bodies of water are coloured in light grey.

Figure S7: Upper bound of 95% uncertainty interval for estimated family planning indicators in Burkina Faso, 2020

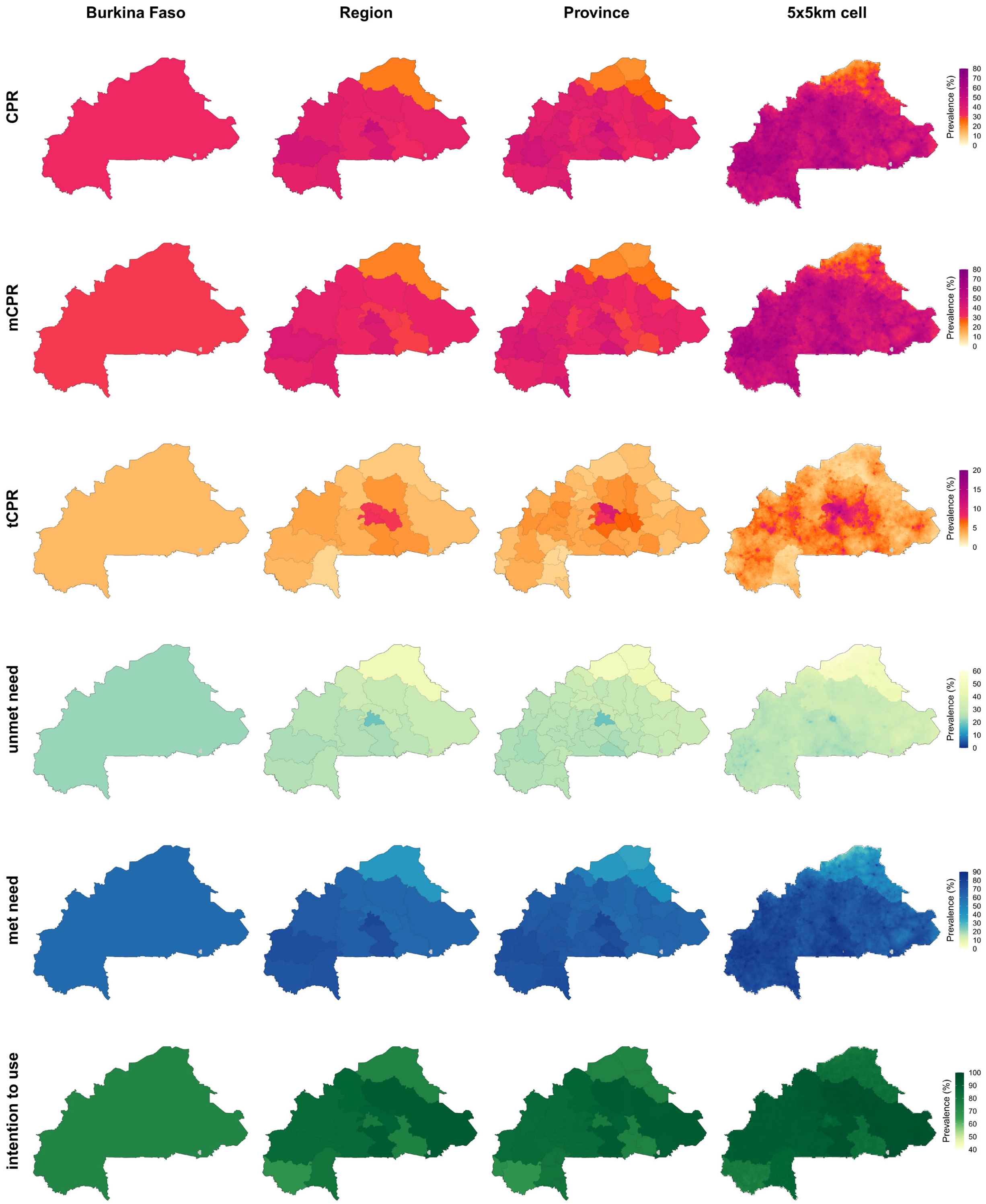

Note: bodies of water are coloured in light grey.

Figure S8: Estimated family planning indicators in Burkina Faso, 2000

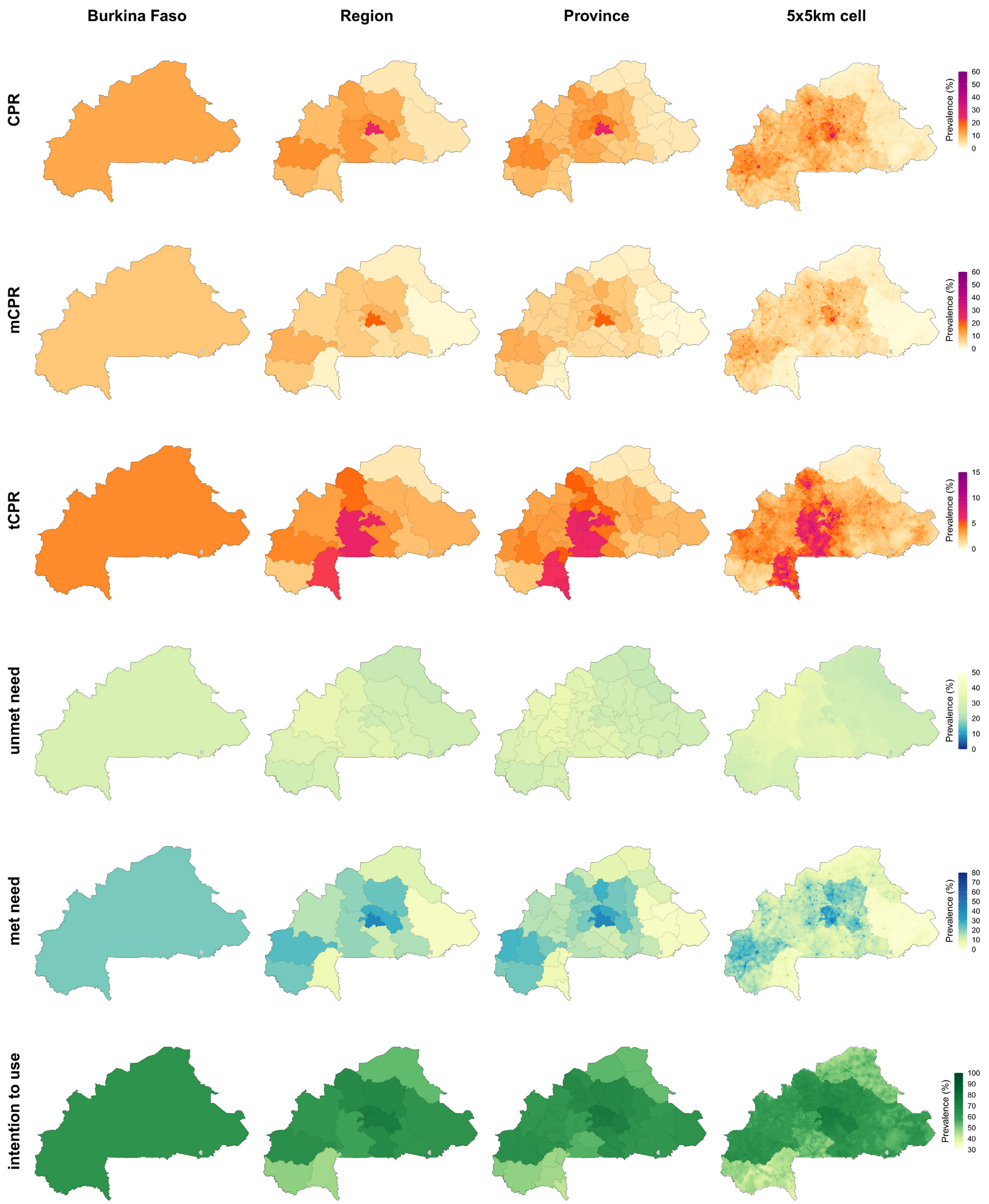

Note: bodies of water are coloured in light grey.

Figure S9: Lower bound of 95% uncertainty interval for estimated family planning indicators in Burkina Faso, 2000

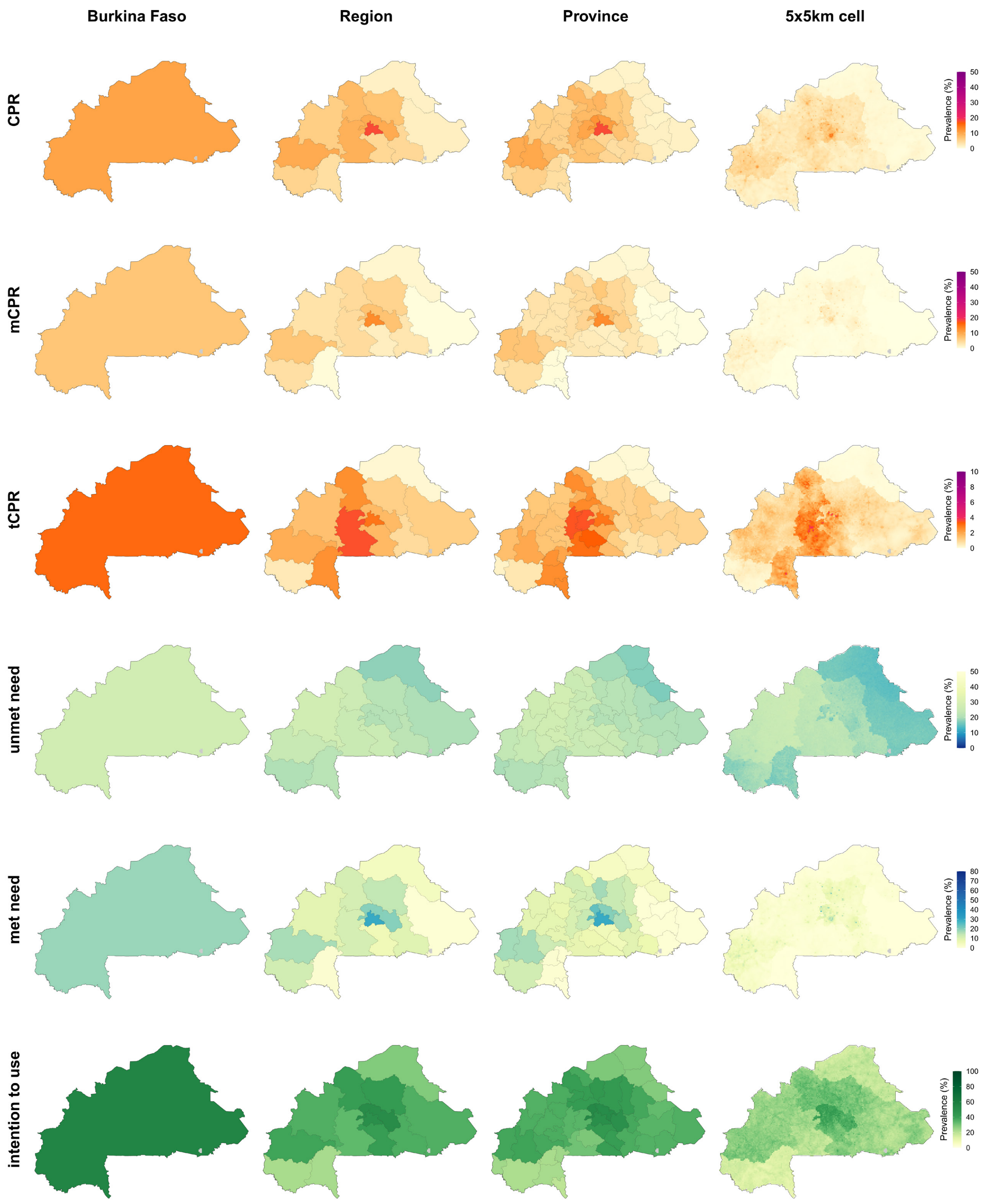

Note: bodies of water are coloured in light grey.

Figure S10: Upper bound of 95% uncertainty interval for estimated family planning indicators in Burkina Faso, 2000

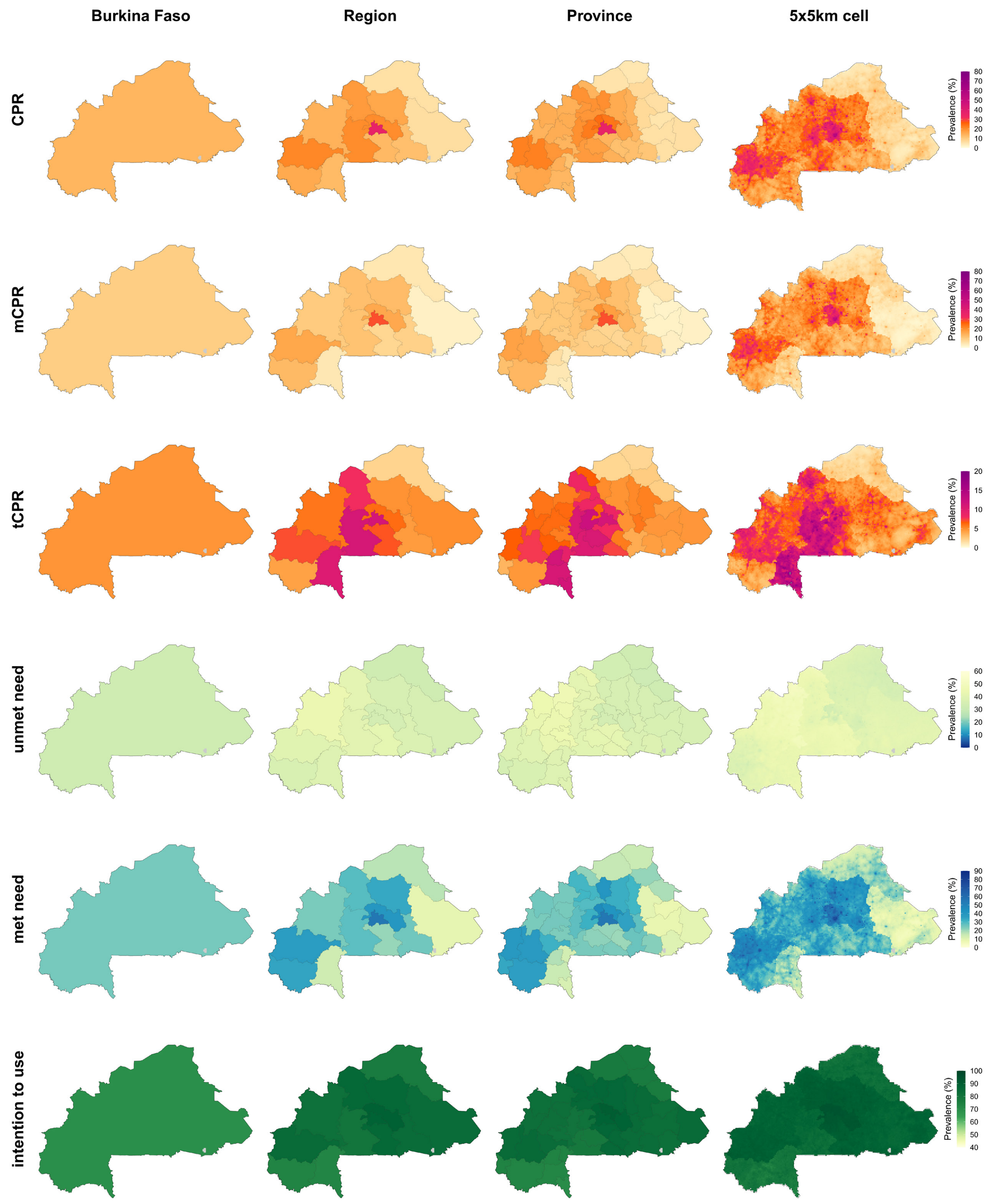

Note: bodies of water are coloured in light grey.

Figure S11: Lower bound of 95% uncertainty interval for estimated family planning indicators in Kenya, 2020

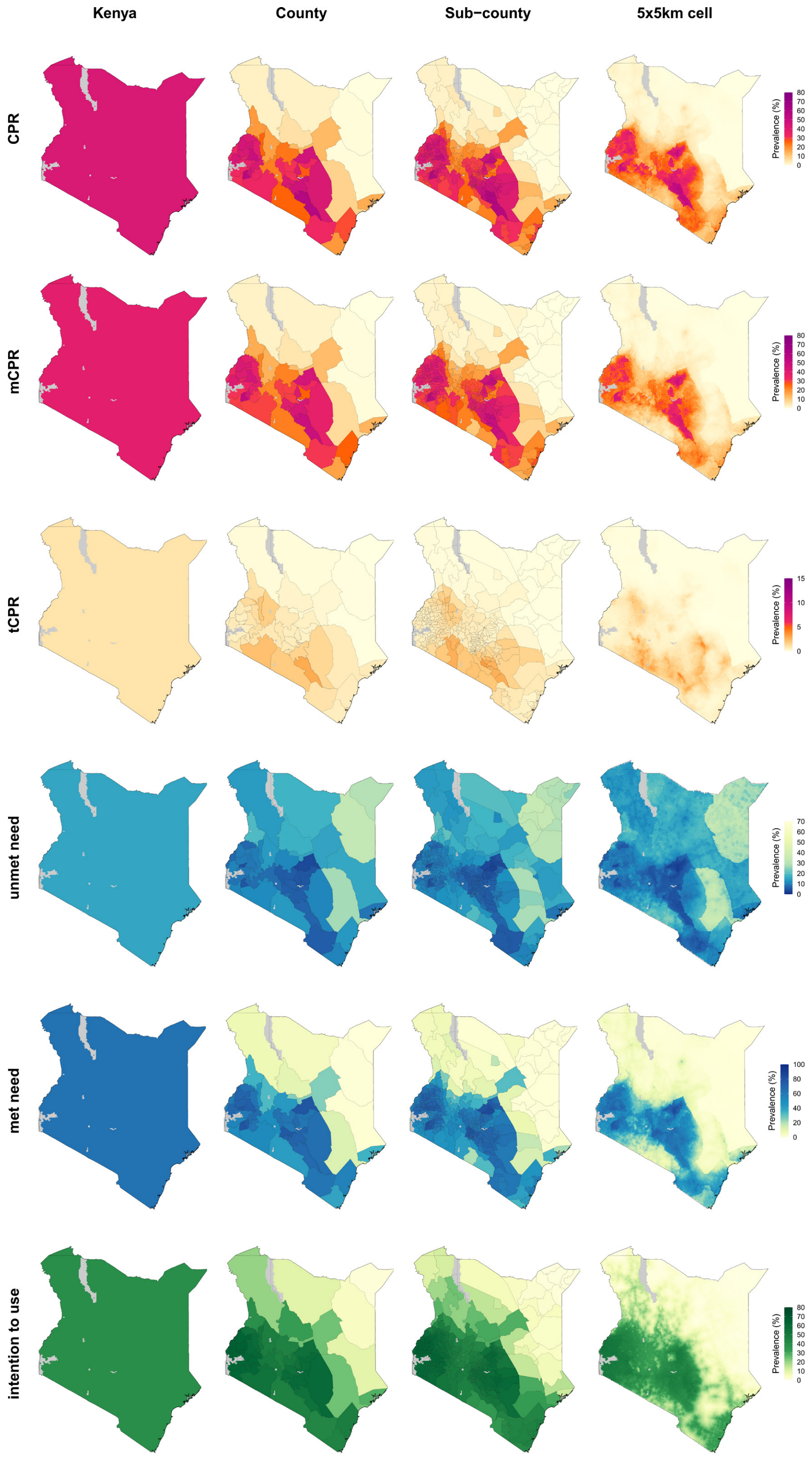

Note: bodies of water are coloured in light grey.

Figure S12: Upper bound of 95% uncertainty interval for estimated family planning indicators in Kenya, 2020

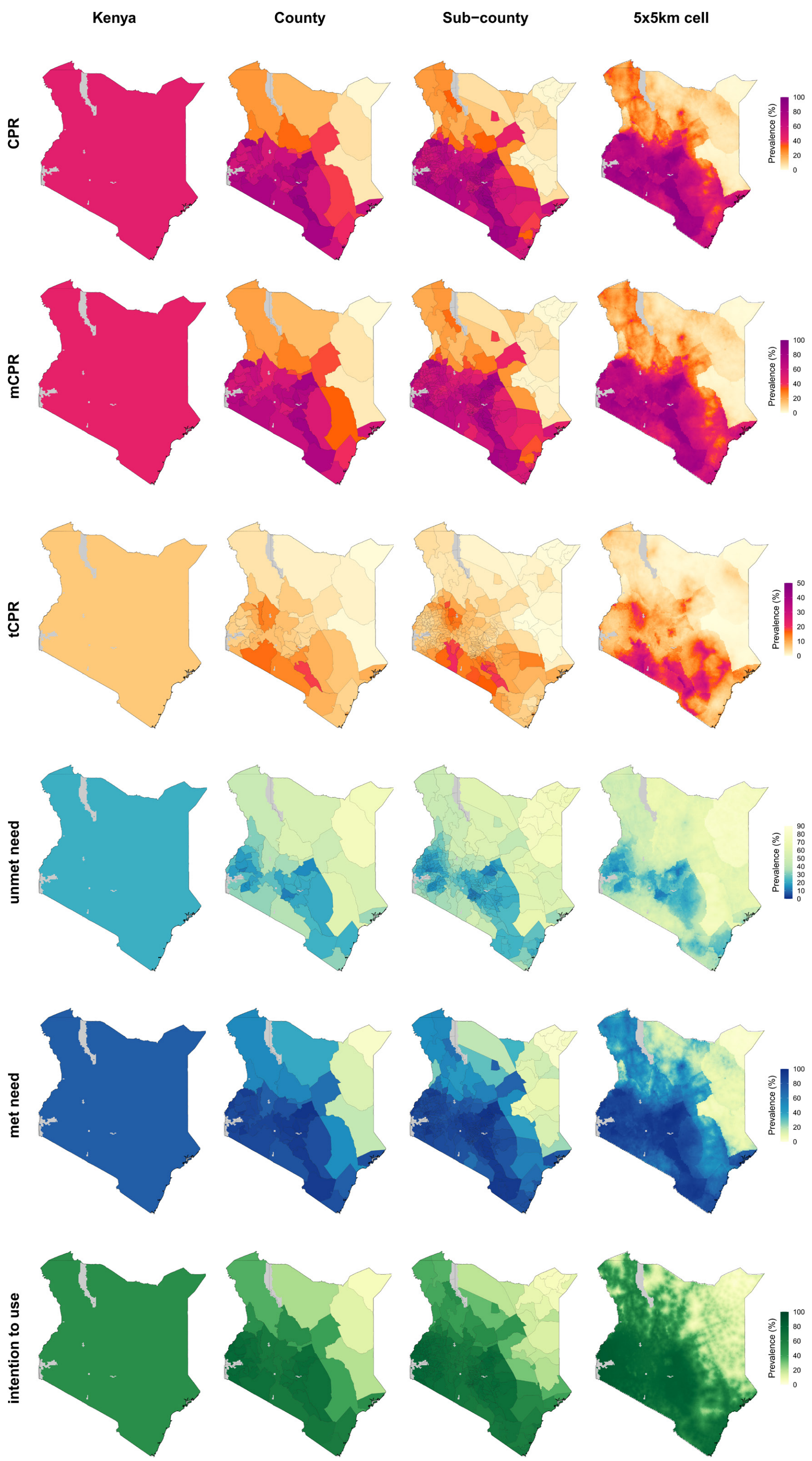

Note: bodies of water are coloured in light grey.

Figure S13: Estimated family planning indicators in Kenya, 2000

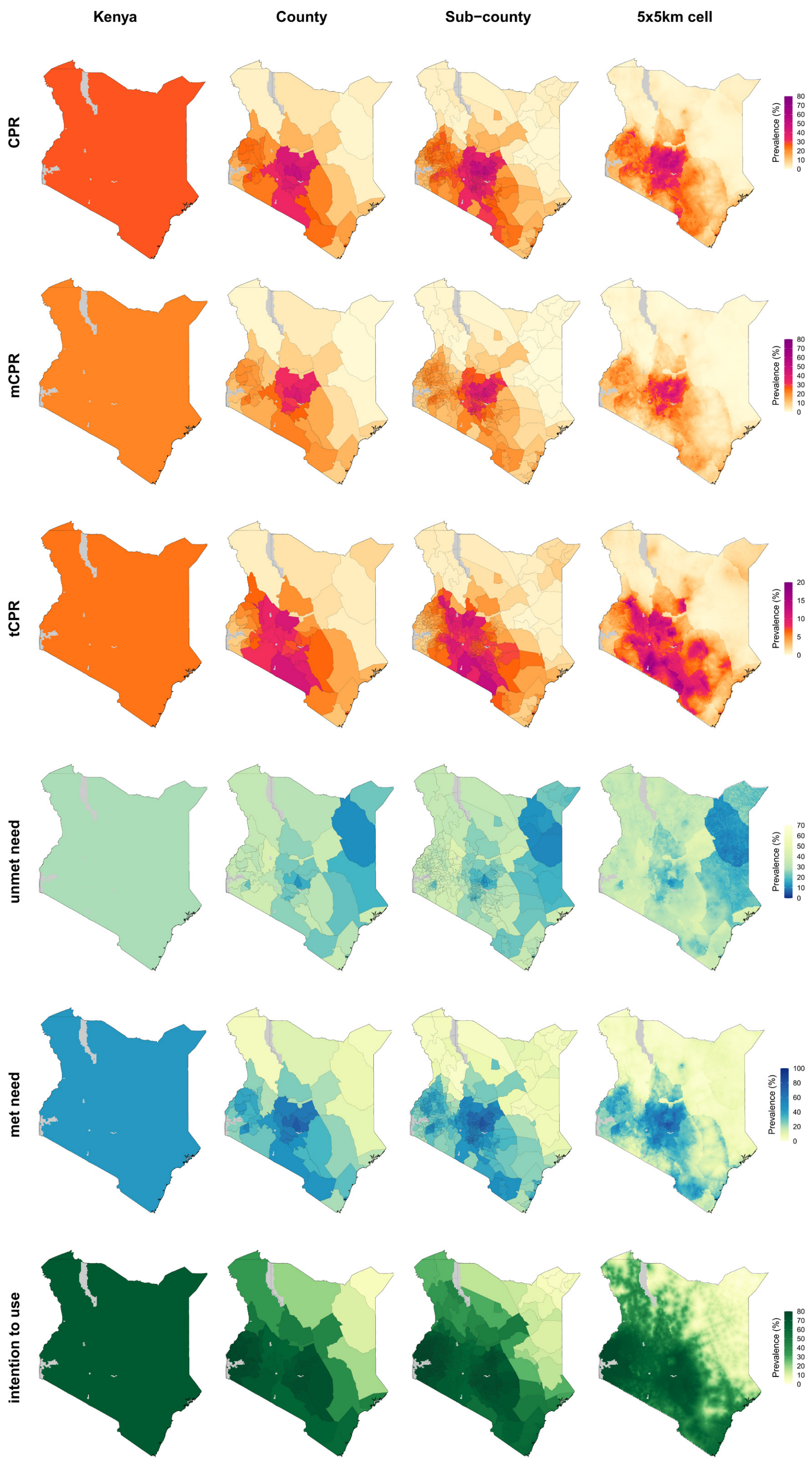

Note: bodies of water are coloured in light grey.

Figure S14: Lower bound of 95% uncertainty interval for estimated family planning indicators in Kenya, 2000

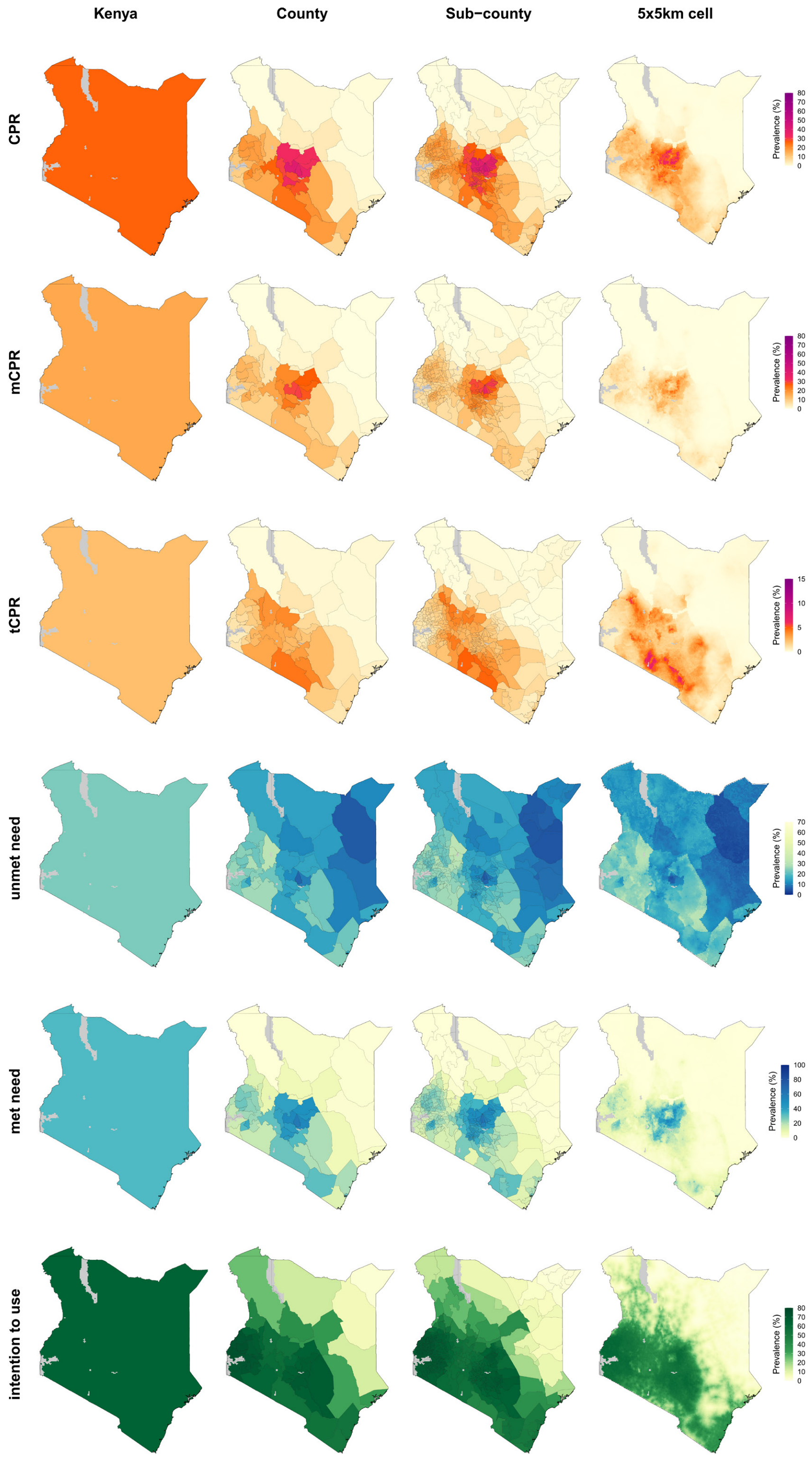

Note: bodies of water are coloured in light grey.

Figure S15: Upper bound of 95% uncertainty interval for estimated family planning indicators in Kenya, 2000

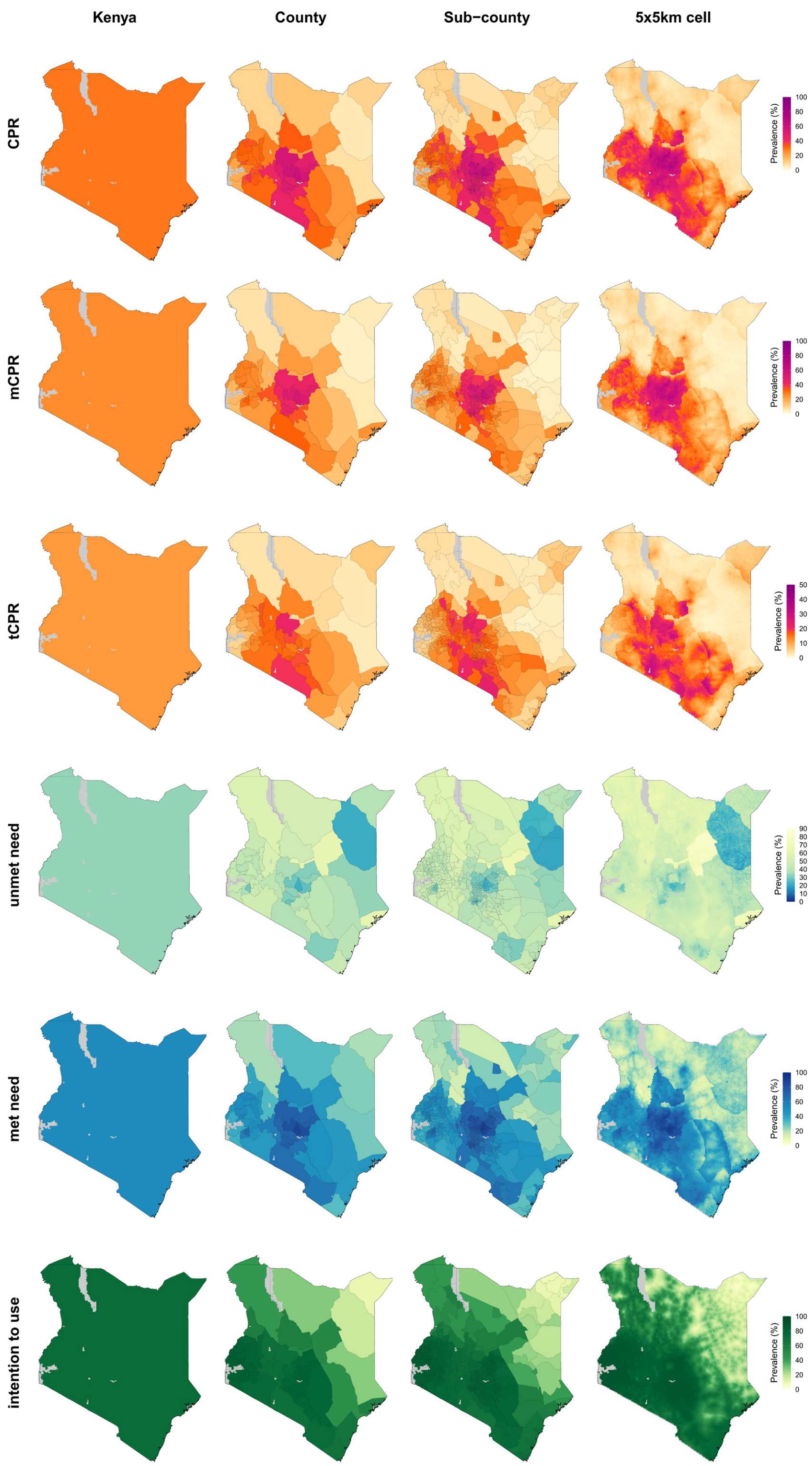

Note: bodies of water are coloured in light grey.

Figure S16: Lower bound of 95% uncertainty interval for estimated family planning indicators in Nigeria, 2020

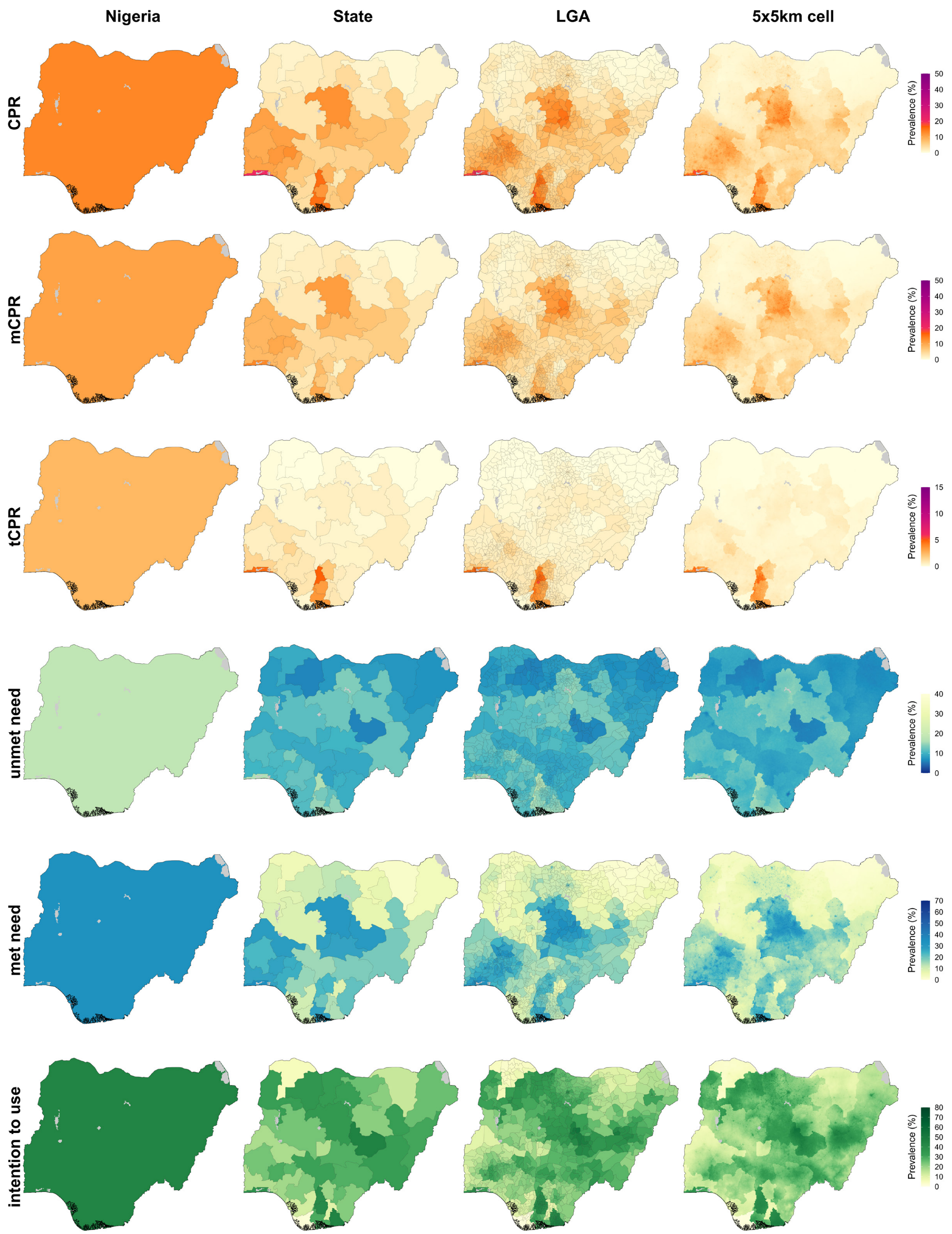

Note: bodies of water are coloured in light grey.

Figure S17: Upper bound of 95% uncertainty interval for estimated family planning indicators in Nigeria, 2020

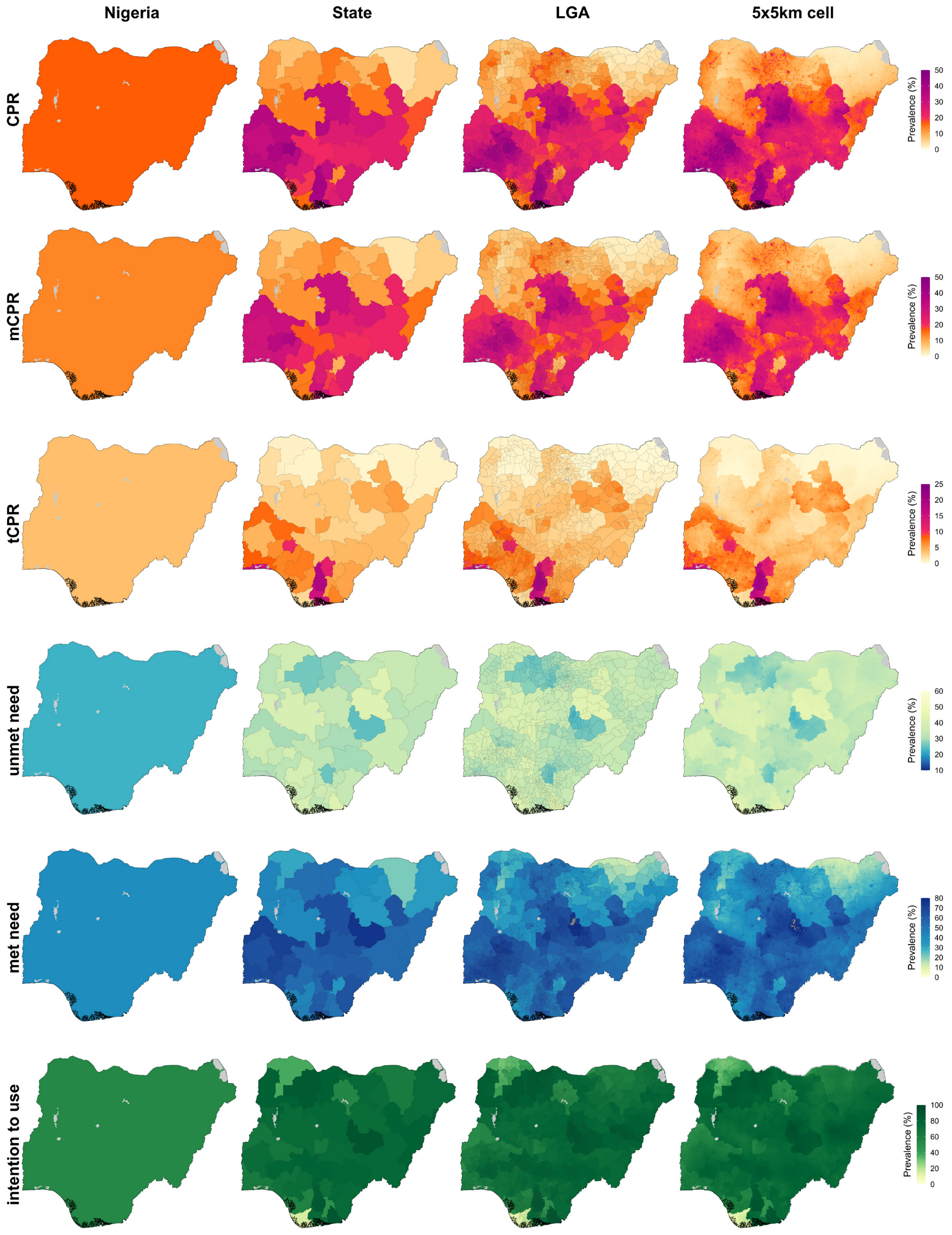

Note: bodies of water are coloured in light grey.

Figure S18: Estimated family planning indicators in Nigeria, 2000

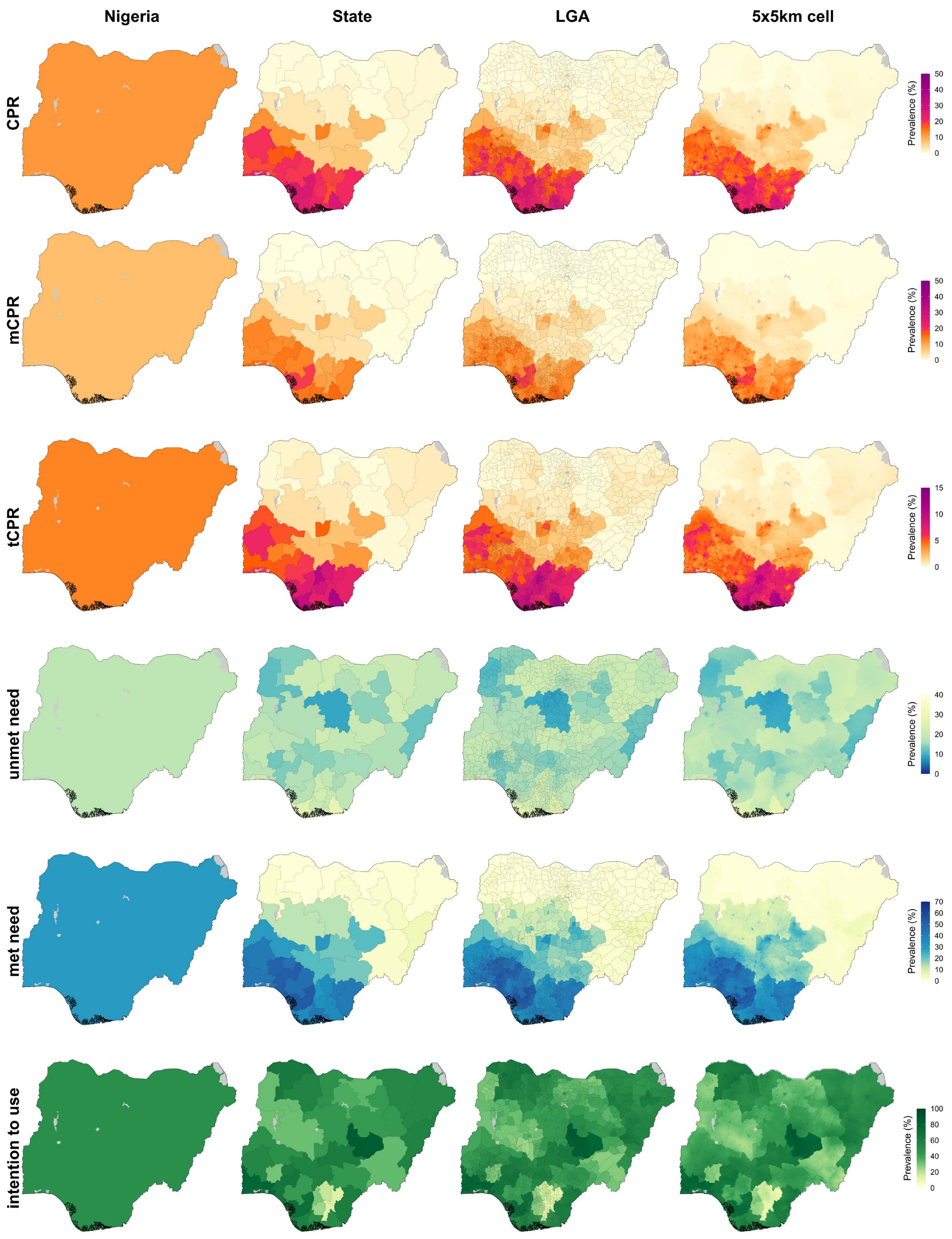

Note: bodies of water are coloured in light grey.

Figure S19: Lower bound of 95% uncertainty interval for estimated family planning indicators in Nigeria, 2000

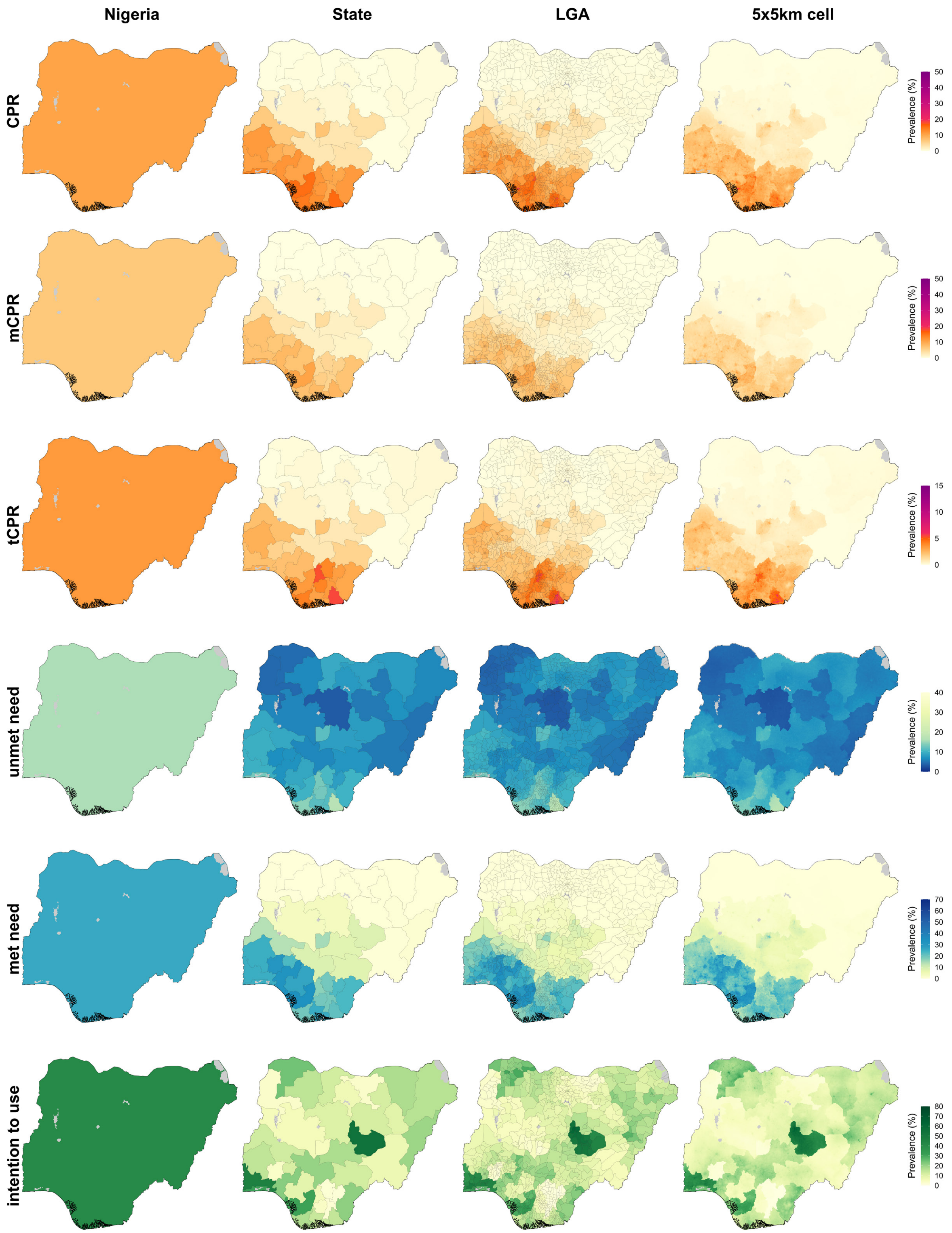

Note: bodies of water are coloured in light grey.

Figure S20: Upper bound of 95% uncertainty interval for estimated family planning indicators in Nigeria, 2000

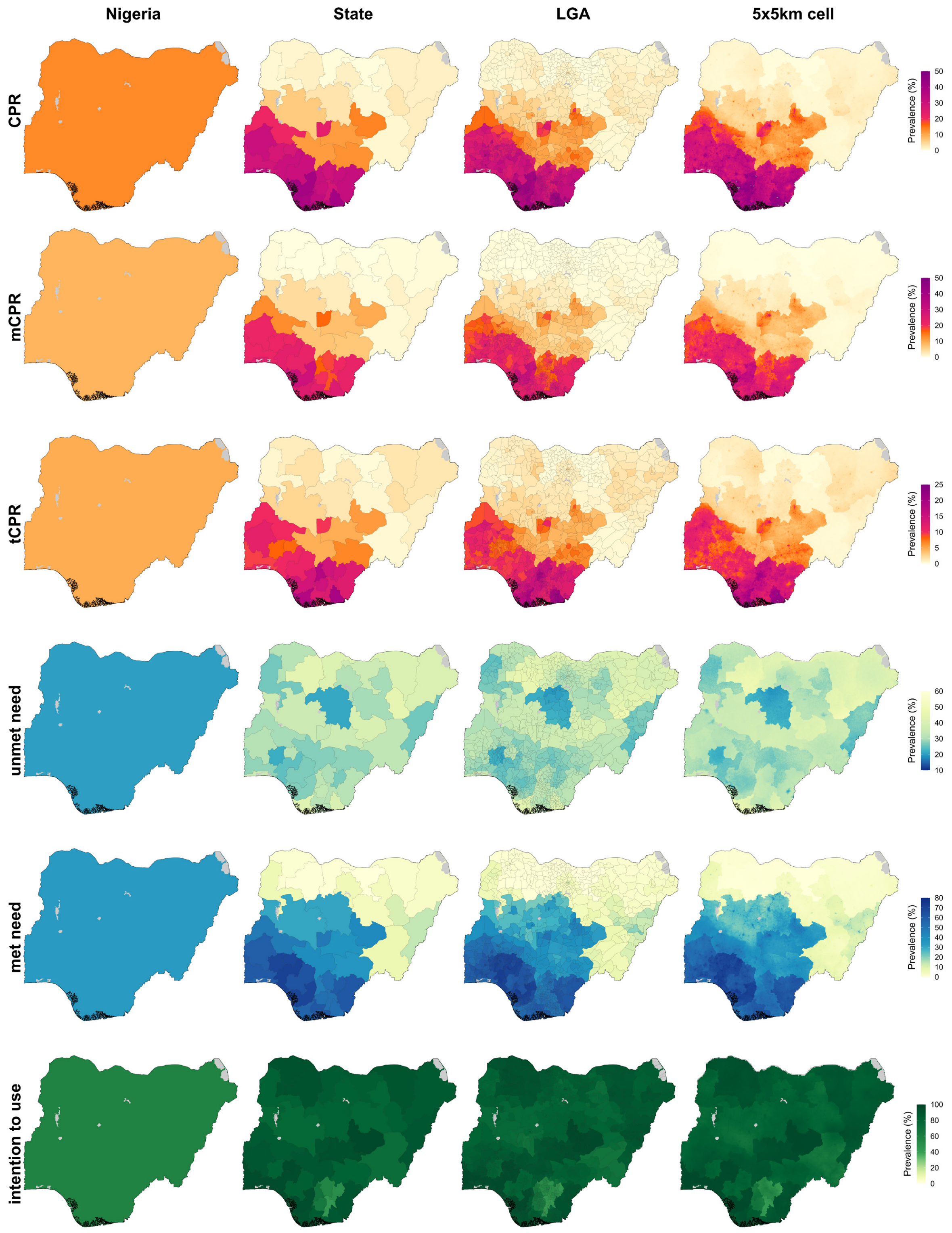

Note: bodies of water are coloured in light grey.

Supplementary Tables

Table S1: Family planning indicator values and differences in definitions

Survey*	Calculated values						Values from survey report						Source of survey values	Explanation of Differences
	CPR	mCPR	tCPR	Unmet need	Demand Satisfied	Intention to Use	CPR	mCPR	tCPR	Unmet need	Demand Satisfied	Intention to Use		
Burkina Faso 1998 Demographic and Health Survey	10.7	5.9	4.8	30.7	16.1	56.5	12.0 (+1.3)	5.8 (-0.1)	6.2 (+1.4)	25.5 (-5.2)	15.5 (-0.6)	44.5 (-12)	https://www.statcompiler.com/en/	*Contraception:* DHS considers abstinence a traditional method of contraception, we do not consider this a method of contraception; LAM is not a method of contraception, we consider this a traditional method of contraception.<br>*Need:* DHS calculates PPA as <24 months, we calculate as ≤24 months; Only menopause is accepted as a reason for infecundity from reason_no_contra, we accept menopause, hysterectomy, infertile, and other text entry options.<br>*Intent:* DHS only calculates intent for married/in-union women, we calculate for all women.
Burkina Faso 2003 Demographic and Health Survey	12.6	9.6	3.0	27.9	25.7	69.9	14.0 (+1.4)	9.8 (+0.2)	4.1 (+1.1)	24.1 (-3.8)	25.8 (+0.1)	58.4 (-11.5)	https://www.statcompiler.com/en/	*Contraception:* DHS considers abstinence a traditional method of contraception, we do not consider this a method of contraception; LAM and SDM are considered modern methods of contraception, we consider these traditional methods of contraception.<br>*Need:* DHS calculates PPA as <24 months, we calculate as ≤24 months; Only menopause is accepted as a reason for infecundity from reason_no_contra, we accept menopause, hysterectomy, infertile, and other text entry options.<br>*Intent:* DHS only calculates intent for married/in-union women, we calculate for all women.

Survey*	Calculated values						Values from survey report						Source of survey values	Explanation of Differences
	CPR	mCPR	tCPR	Unmet need	Demand Satisfied	Intention to Use	CPR	mCPR	tCPR	Unmet need	Demand Satisfied	Intention to Use		
Burkina Faso 2006 Multiple Indicator Cluster Survey	16.4	13.0	3.4	45.9	22.1		17.4 (+1)	13.3 (+0.3)	4.2 (+0.8)	31.1 (-14.8)	35.9 (+13.8)		https://mics.unicef.org/surveys	*Contraception:* MICS only calculates indicators for married/in-union women, we calculate for all women. *Need:* MICS does not include PPA in need calculations, we calculate PPA within the past 24 months and designate these women as not in need of contraception unless they did not desire this last pregnancy and current PPA status; fecundity determined based on response, not calculated, we calculate using information captured regarding length of cohabitation, ever using contraception, and lack of children; desire only determined for women with children, we determine desire for all women.
Burkina Faso 2010 Demographic and Health Survey	15.5	14.4	1.1	22.0	39.5	64.1	15.3 (-0.2)	14.3 (-0.1)	1.0 (-0.1)	20.4 (-1.6)	39.9 (+0.4)	59.2 (-4.9)	https://www.statcompiler.com/en/	*Contraception:* DHS considers LAM and SDM modern methods of contraception, we consider these traditional methods of contraception. *Need:* DHS calculates PPA as <24 months, we calculate as ≤24 months; Only menopause is accepted as a reason for infecundity from reason_no_contra, we accept menopause, hysterectomy, infertile, and other text entry options. *Intent:* DHS only calculates intent for married/in-union women, we calculate for all women.
Burkina Faso 2014 PMA2020 Round 1	15.9	15.7	0.2	32.0	32.9	62.2	15.8 (-0.1)	15.7 (-0)	0.1 (-0.1)	31.5 (-0.5)	33.3 (+0.4)		https://datalab.pmadata.org/ https://www.pmadata.org/countries/burkina-faso	*Contraception:* PMA2020 considers women not currently using contraception, but who used emergency contraceptives in the last 12 months as current contraception users, we only consider emergency contraceptives a method contraception if it is recorded as a current method; reported LAM is considered a modern method only if most recent birth was in the last 6 months and last reported menses was before that birth, we consider all LAM a traditional method of contraception. *Need:* PMA2020 calculates PPA for births <24 months, we calculate using ≤24 months; infecundity calculations for last birth in 5 years calculated as ≥60 months, we use >60 months; only menopause is accepted as a reason for infecundity from reason_no_contra, we accept menopause, hysterectomy, infertile, and other text entry options.

Survey*	Calculated values						Values from survey report						Source of survey values	Explanation of Differences
	CPR	mCPR	tCPR	Unmet need	Demand Satisfied	Intention to Use	CPR	mCPR	tCPR	Unmet need	Demand Satisfied	Intention to Use		
Burkina Faso 2015 PMA2020 Round 2	19.4	18.4	1.0	28.4	39.2	65.6	19.5 (+0.1)	18.6 (+0.2)	0.9 (-0.1)	27.1 (-1.3)	39.9 (+0.7)		https://datalab.pmadata.org/ https://www.pmadata.org/countries/burkina-faso	*Contraception:* PMA2020 considers women not currently using contraception, but who used emergency contraceptives in the last 12 months as current contraception users, we only consider emergency contraceptives a method contraception if it is recorded as a current method; reported LAM is considered a modern method only if most recent birth was in the last 6 months and last reported menses was before that birth, we consider all LAM a traditional method of contraception. *Need:* PMA2020 calculates PPA for births <24 months, we calculate using ≤24 months; infecundity calculations for last birth in 5 years calculated as ≥60 months, we use >60 months; only menopause is accepted as a reason for infecundity from reason_no_contra, we accept menopause, hysterectomy, infertile, and other text entry options.
Burkina Faso 2016 PMA2020 Round 3	22.8	21.4	1.3	25.1	46.0	67.2	22.8 (-0)	21.5 (+0.1)	1.3 (-0)	24.2 (-0.9)	45.8 (-0.2)		https://datalab.pmadata.org/ https://www.pmadata.org/countries/burkina-faso	*Contraception*: PMA2020 considers women not currently using contraception, but who used emergency contraceptives in the last 12 months as current contraception users, we only consider emergency contraceptives a method contraception if it is recorded as a current method; LAM is not considered a method of contraception, we consider LAM a traditional method of contraception. *Need*: PMA2020 calculates PPA for births <24 months, we calculate using ≤24 months; infecundity calculations for last birth in 5 years calculated as ≥60 months, we use >60 months; only menopause is accepted as a reason for infecundity from reason_no_contra, we accept menopause, hysterectomy, infertile, and other text entry options.

Survey*	Calculated values						Values from survey report						Source of survey values	Explanation of Differences
	CPR	mCPR	tCPR	Unmet need	Demand Satisfied	Intention to Use	CPR	mCPR	tCPR	Unmet need	Demand Satisfied	Intention to Use		
Burkina Faso 2016–2017 PMA2020 Round 4	22.8	21.8	1.0	25.3	46.3	73.4	22.9 (+0.1)	21.9 (+0.1)	0.9 (-0.1)	24.4 (-0.9)	46.3 (-0)		https://datalab.pmadata.org/ https://www.pmadata.org/countries/burkina-faso	*Contraception:* PMA2020 considers women not currently using contraception, but who used emergency contraceptives in the last 12 months as current contraception users, we only consider emergency contraceptives a method contraception if it is recorded as a current method; LAM is not considered a method of contraception, we consider LAM a traditional method of contraception. *Need:* PMA2020 calculates PPA for births <24 months, we calculate using ≤24 months; infecundity calculations for last birth in 5 years calculated as ≥60 months, we use >60 months; only menopause is accepted as a reason for infecundity from reason_no_contra, we accept menopause, hysterectomy, infertile, and other text entry options.
Burkina Faso 2017–2018 PMA2020 Round 5	28.0	26.2	1.8	20.2	56.4	75.6	28.0 (-0)	26.4 (+0.2)	1.6 (-0.2)	18.8 (-1.4)	56.3 (-0.1)		https://datalab.pmadata.org/ https://www.pmadata.org/countries/burkina-faso	*Contraception:* PMA2020 considers women not currently using contraception, but who used emergency contraceptives in the last 12 months as current contraception users, we only consider emergency contraceptives a method contraception if it is recorded as a current method; reported LAM is considered a modern method only if most recent birth was in the last 6 months and last reported menses was before that birth, we consider all LAM a traditional method of contraception. *Need:* PMA2020 calculates PPA for births <24 months, we calculate using ≤24 months; infecundity calculations for last birth in 5 years calculated as ≥60 months, we use >60 months; only menopause is accepted as a reason for infecundity from reason_no_contra, we accept menopause, hysterectomy, infertile, and other text entry options.

Survey*	Calculated values						Values from survey report						Source of survey values	Explanation of Differences
	CPR	mCPR	tCPR	Unmet need	Demand Satisfied	Intention to Use	CPR	mCPR	tCPR	Unmet need	Demand Satisfied	Intention to Use		
Burkina Faso 2018–2019 PMA2020 Round 6	28.8	27.0	1.8	20.1	57.4	73.4	29.0 (+0.2)	27.3 (+0.3)	1.8 (-0)	19.1 (-1)	56.6 (-0.8)		https://datalab.pmadata.org/ https://www.pmadata.org/countries/burkina-faso	*Contraception:* PMA2020 considers women not currently using contraception, but who used emergency contraceptives in the last 12 months as current contraception users, we only consider emergency contraceptives a method contraception if it is recorded as a current method; reported LAM is considered a modern method only if most recent birth was in the last 6 months and last reported menses was before that birth, we consider all LAM a traditional method of contraception. *Need:* PMA2020 calculates PPA for births <24 months, we calculate using ≤24 months; infecundity calculations for last birth in 5 years calculated as ≥60 months, we use >60 months; only menopause is accepted as a reason for infecundity from reason_no_contra, we accept menopause, hysterectomy, infertile, and other text entry options.
Burkina Faso 2020 PMA Phase 1	27.3	25.3	2.0	22.7	52.7	71.2	27.4 (+0.1)	25.5 (+0.2)	1.9 (-0.1)	21.1 (-1.6)	52.6 (-0.1)	26.3 (-44.9)	https://datalab.pmadata.org/ https://www.pmadata.org/countries/burkina-faso	*Contraception:* PMA considers women not currently using contraception, but who used emergency contraceptives in the last 12 months as current contraception users, we only consider emergency contraceptives a method contraception if it is recorded as a current method; reported LAM is considered a modern method only if most recent birth was in the last 6 months and last reported menses was before that birth, we consider all LAM a traditional method of contraception; SDM is considered a modern method of contraception, we consider this a traditional method of contraception. *Need:* PMA calculates PPA for births <24 months, we calculate using ≤24 months; infecundity calculations for last birth in 5 years calculated as ≥60 months, we use >60 months; only menopause is accepted as a reason for infecundity from reason_no_contra, we accept menopause, hysterectomy, infertile, and other text entry options. *Intent:* PMA calculates intent for women who are not pregnant and intend to use contraception within 12 months, we calculate intent for all women with no time limit for future intent use.

Survey*	Calculated values						Values from survey report						Source of survey values	Explanation of Differences
	CPR	mCPR	tCPR	Unmet need	Demand Satisfied	Intention to Use	CPR	mCPR	tCPR	Unmet need	Demand Satisfied	Intention to Use		
Burkina Faso 2020 PMA Phase 2	32.5	29.1	3.3	20.9	58.2	69.2	32.3 (-0.2)	29.2 (+0.1)	3.1 (-0.2)	17.2 (-3.7)	59.0 (+0.8)	27.0 (-42.2)	https://datalab.pmadata.org/ https://www.pmadata.org/countries/burkina-faso	*Contraception:* PMA considers women not currently using contraception, but who used emergency contraceptives in the last 12 months as current contraception users, we only consider emergency contraceptives a method contraception if it is recorded as a current method; reported LAM is considered a modern method only if most recent birth was in the last 6 months and last reported menses was before that birth, we consider all LAM a traditional method of contraception; SDM is considered a modern method of contraception, we consider this a traditional method of contraception.<br>*Need:* PMA calculates PPA for births <24 months, we calculate using ≤24 months; infecundity calculations for last birth in 5 years calculated as ≥60 months, we use >60 months; only menopause is accepted as a reason for infecundity from reason_no_contra, we accept menopause, hysterectomy, infertile, and other text entry options.<br>*Intent:* PMA calculates intent for women who are not pregnant and intend to use contraception within 12 months, we calculate intent for all women with no time limit for future intent use.
Kenya 1998 Demographic and Health Survey	30.4	23.6	6.8	27.2	46.4	75.5	29.9 (-0.5)	23.6 (-0)	6.3 (-0.5)	20.9 (-6.3)	46.4 (-0)	63.3 (-12.2)	https://www.statcompiler.com/en/	*Contraception:* DHS does not consider LAM a method of contraception, we consider it a traditional method of contraception.<br>*Need:* DHS calculates PPA as <24 months, we calculate as ≤24 months; Only menopause is accepted as a reason for infecundity from reason_no_contra, we accept menopause, hysterectomy, infertile, and other text entry options.<br>*Intent:* DHS only calculates intent for married/in-union women, we calculate for all women.

Survey*	Calculated values						Values from survey report						Source of survey values	Explanation of Differences
	CPR	mCPR	tCPR	Unmet need	Demand Satisfied	Intention to Use	CPR	mCPR	tCPR	Unmet need	Demand Satisfied	Intention to Use		
Kenya 2003 Demographic and Health Survey	28.3	22.7	5.6	24.8	47.8	72.2	28.4 (+0.1)	22.7 (-0)	5.6 (-0)	19.5 (-5.3)	47.5 (-0.3)	57.8 (-14.4)	https://www.statcompiler.com/en/	*Contraception:* DHS considers LAM and SDM modern methods of contraception, we consider them traditional methods of contraception. *Need:* DHS calculates PPA as <24 months, we calculate as ≤24 months; Only menopause is accepted as a reason for infecundity from reason_no_contra, we accept menopause, hysterectomy, infertile, and other text entry options. *Intent:* DHS only calculates intent for married/in-union women, we calculate intent for all women.
Kenya 2007 AIDS Indicator Survey	28.7	27.0	1.6	23.2	53.8					52.4 (+29.2)	45.0 (-8.8)		https://stacks.cdc.gov/view/cdc/12122	*Contraception:* AIS only calculates unmet need and demand satisfied for married/in-union women who are in need of contraception, we calculate for all women who are in need of contraception.
Kenya 2007 Multiple Indicator Cluster Survey (Eastern Province)	5.4	0.5	4.9	44.0	1.1									
Kenya 2008 Multiple Indicator Cluster Survey (Eastern Province)	33.9	30.3	3.6	33.2	47.7									

Survey*	Calculated values						Values from survey report						Source of survey values	Explanation of Differences
	CPR	mCPR	tCPR	Unmet need	Demand Satisfied	Intention to Use	CPR	mCPR	tCPR	Unmet need	Demand Satisfied	Intention to Use		
Kenya 2008–2009 Demographic and Health Survey	32.0	27.6	4.4	22.3	55.3	64.6	32.0 (-0)	28.0 (+0.4)	4.1 (-0.3)	17.9 (-4.4)	55.9 (+0.6)	55.0 (-9.6)	https://www.statcompiler.com/en/	*Contraception:* DHS considers LAM and SDM modern methods of contraception, we consider them traditional methods of contraception. *Need:* DHS calculates PPA as <24 months, we calculate as ≤24 months; Only menopause is accepted as a reason for infecundity from reason_no_contra, we accept menopause, hysterectomy, infertile, and other text entry options. *Intent:* DHS only calculates intent for married/in-union women, we calculate intent for all women.
Kenya 2010 Urban Reproductive Health Initiative	45.3	40.6	4.7	15.1	72.9	37.1								
Kenya 2011 Multiple Indicator Cluster Survey (Nyanza Province)	37.4	35.1	2.3	30.7	53.3		47.5 (+10.1)	44.8 (+9.7)	2.7 (+0.4)				https://mics.unicef.org/surveys	MICS only calculates indicators for married/in-union women, we calculate for all women.
Kenya 2012 AIDS Indicator Survey	53.2	46.3	7.0	22.8	67.0					36.0 (+13.2)	60.8 (-6.2)		https://nacc.or.ke/wp-content/uploads/2015/10/KAIS-2012.pdf	AIS only calculates unmet need and demand satisfied for married/in-union women who are in need of contraception, we calculate for all women who are in need of contraception.

Survey*	Calculated values						Values from survey report						Source of survey values	Explanation of Differences
	CPR	mCPR	tCPR	Unmet need	Demand Satisfied	Intention to Use	CPR	mCPR	tCPR	Unmet need	Demand Satisfied	Intention to Use		
Kenya 2013 Multiple Indicator Cluster Survey (Bungoma County)	36.6	35.5	1.0	16.2	68.7		54.4 (+17.8)	53.3 (+17.8)	0.6 (-0.4)	22.5 (+6.3)	70.7 (+2)		https://mics.unicef.org/surveys	MICS only calculates indicators for married/in-union women, we calculate for all women. *Contraception:* MICS considers LAM and SDM modern methods of contraception, we consider them traditional methods of contraception. *Need:* MICS calculates infecundity for women with no period >6 months, we use ≥6 months; 5 year timespan since last birth for married women calculated as ≥60 months, we use >60 months.
Kenya 2013 Multiple Indicator Cluster Survey (Kakamega County)	45.0	43.4	1.6	18.9	69.7		61.5 (+16.5)	59.3 (+15.9)	2.2 (+0.6)	22.2 (+3.3)	73.5 (+3.8)		https://mics.unicef.org/surveys	MICS only calculates indicators for married/in-union women, we calculate for all women. *Contraception*: MICS considers LAM and SDM modern methods of contraception, we consider them traditional methods of contraception. *Need*: MICS calculates infecundity for women with no period >6 months, we use ≥6 months; 5 year timespan since last birth for married women calculated as ≥60 months, we use >60 months.
Kenya 2013 Multiple Indicator Cluster Survey (Turkana County)	10.1	9.7	0.4	24.7	28.2		14.1 (+4)	13.3 (+3.6)	0.5 (+0.1)	34.0 (+9.3)	29.2 (+1)		https://mics.unicef.org/surveys	MICS only calculates indicators for married/in-union women, we calculate for all women. *Contraception*: MICS considers LAM and SDM modern methods of contraception, we consider them traditional methods of contraception. *Need*: MICS calculates infecundity for women with no period >6 months, we use ≥6 months; 5 year timespan since last birth for married women calculated as ≥60 months, we use >60 months.
Kenya 2014 Demographic and Health Survey (long form)	43.0	39.4	3.6	16.3	70.8	66.6	42.6 (-0.4)	39.1 (-0.3)	3.5 (-0.1)	12.8 (-3.5)	70.8 (-0)	57.3 (-9.3)	https://www.statcompiler.com/en/	*Contraception*: DHS considers LAM and SDM modern methods of contraception, we consider them traditional methods of contraception. *Need*: DHS calculates PPA as <24 months, we calculate as ≤24 months; Only menopause is accepted as a reason for infecundity from reason_no_contra, we accept menopause, hysterectomy, infertile, and other text entry options. *Intent*: DHS only calculates intent for married/in-union women, we calculate for all women.

Survey*	Calculated values						Values from survey report						Source of survey values	Explanation of Differences
	CPR	mCPR	tCPR	Unmet need	Demand Satisfied	Intention to Use	CPR	mCPR	tCPR	Unmet need	Demand Satisfied	Intention to Use		
Kenya 2014 Demographic and Health Survey (short form)	42.2	38.7	3.5	20.8	65.1									
Kenya 2014 PMA2020 Round 1	41.8	41.2	0.7	17.3	70.4	65.2	42.7 (+0.9)	42.5 (+1.3)	0.2 (-0.5)	15.7 (-1.6)	72.8 (+2.4)		https://datalab.pmadata.org/ https://www.pmadata.org/countries/kenya	*Contraception*: PMA2020 considers women not currently using contraception, but who used emergency contraceptives in the last 12 months as current contraception users, we only consider emergency contraceptives a method contraception if it is recorded as a current method; LAM and SDM are considered modern methods of contraception, we consider them traditional methods of contraception; "Don't know" responses to current use considered using a method of contraception, we consider them as not using contraception. *Need*: PMA2020 calculates PPA for births <24 months, we calculate using ≤24 months; PPA only calculated using entered response, we calculate using timing between most recent birth and last menses; infecundity calculations for last birth in 5 years calculated as ≥60 months, we use >60 months; only menopause is accepted as a reason for infecundity from reason_no_contra, we accept menopause, hysterectomy, infertile, and other text entry options.
Kenya 2014 PMA2020 Round 2	41.0	39.6	1.4	16.2	71.1	59.1	41.3 (+0.3)	40.8 (+1.2)	0.5 (-0.9)	13.5 (-2.7)	74.5 (+3.4)		https://datalab.pmadata.org/ https://www.pmadata.org/countries/kenya	*Contraception*: PMA2020 considers women not currently using contraception, but who used emergency contraceptives in the last 12 months as current contraception users, we only consider emergency contraceptives a method contraception if it is recorded as a current method; LAM and SDM are considered modern methods of contraception, we consider them traditional methods of contraception. *Need*: PMA2020 calculates PPA for births <24 months, we calculate using ≤24 months; infecundity calculations for last birth in 5 years calculated as ≥60 months, we use >60 months; only menopause is accepted as a reason for infecundity from reason_no_contra, we accept menopause, hysterectomy, infertile, and other text entry options.

Survey*	Calculated values						Values from survey report						Source of survey values	Explanation of Differences
	CPR	mCPR	tCPR	Unmet need	Demand Satisfied	Intention to Use	CPR	mCPR	tCPR	Unmet need	Demand Satisfied	Intention to Use		
Kenya 2015 PMA2020 Round 3	48.3	46.1	2.2	16.0	74.3	69.8	49.0 (+0.7)	47.2 (+1.1)	1.8 (-0.4)	13.4 (-2.6)	75.6 (+1.3)		https://datalab.pmadata.org/ https://www.pmadata.org/countries/kenya	*Contraception*: PMA2020 considers women not currently using contraception, but who used emergency contraceptives in the last 12 months as current contraception users, we only consider emergency contraceptives a method contraception if it is recorded as a current method; reported LAM is considered a modern method only if most recent birth was in the last 6 months and last reported menses was before that birth, we consider LAM a traditional method of contraception. *Need*: PMA2020 calculates PPA for births <24 months, we calculate using ≤24 months; infecundity calculations for last birth in 5 years calculated as ≥60 months, we use >60 months; only menopause is accepted as a reason for infecundity from reason_no_contra, we accept menopause, hysterectomy, infertile, and other text entry options.
Kenya 2015 PMA2020 Round 4	47.3	45.3	2.0	15.1	75.0	64.0	47.8 (+0.5)	46.0 (+0.7)	1.8 (-0.2)	13.5 (-1.6)	75.0 (-0)		https://datalab.pmadata.org/ https://www.pmadata.org/countries/kenya	*Contraception*: PMA2020 considers women not currently using contraception, but who used emergency contraceptives in the last 12 months as current contraception users, we only consider emergency contraceptives a method contraception if it is recorded as a current method; LAM is not considered a method of contraception, we consider it a traditional method of contraception. *Need*: PMA2020 calculates PPA for births <24 months, we calculate using ≤24 months; infecundity calculations for last birth in 5 years calculated as ≥60 months, we use >60 months; only menopause is accepted as a reason for infecundity from reason_no_contra, we accept menopause, hysterectomy, infertile, and other text entry options.

Survey*	Calculated values						Values from survey report						Source of survey values	Explanation of Differences
	CPR	mCPR	tCPR	Unmet need	Demand Satisfied	Intention to Use	CPR	mCPR	tCPR	Unmet need	Demand Satisfied	Intention to Use		
Kenya 2016 PMA2020 Round 5	45.1	43.5	1.6	14.2	75.4	67.4	45.6 (+0.5)	44.2 (+0.7)	1.4 (-0.2)	12.9 (-1.3)	75.6 (+0.2)		https://datalab.pmadata.org/ https://www.pmadata.org/countries/kenya	*Contraception*: PMA2020 considers women not currently using contraception, but who used emergency contraceptives in the last 12 months as current contraception users, we only consider emergency contraceptives a method contraception if it is recorded as a current method; LAM is not considered a method of contraception, we consider it a traditional method of contraception. *Need*: PMA2020 calculates PPA for births <24 months, we calculate using ≤24 months; infecundity calculations for last birth in 5 years calculated as ≥60 months, we use >60 months; only menopause is accepted as a reason for infecundity from reason_no_contra, we accept menopause, hysterectomy, infertile, and other text entry options.
Kenya 2017 PMA2020 Round 6	44.1	42.6	1.6	13.5	75.9	62.5	45.0 (+0.9)	43.7 (+1.1)	1.3 (-0.3)	12.5 (-1)	76.0 (+0.1)		https://datalab.pmadata.org/ https://www.pmadata.org/countries/kenya	*Contraception*: PMA2020 considers women not currently using contraception, but who used emergency contraceptives in the last 12 months as current contraception users, we only consider emergency contraceptives a method contraception if it is recorded as a current method; reported LAM is considered a modern method only if most recent birth was in the last 6 months and last reported menses was before that birth, we consider LAM a traditional method of contraception. *Need*: PMA2020 calculates PPA for births <24 months, we calculate using ≤24 months; infecundity calculations for last birth in 5 years calculated as ≥60 months, we use >60 months; only menopause is accepted as a reason for infecundity from reason_no_contra, we accept menopause, hysterectomy, infertile, and other text entry options.

Survey*	Calculated values						Values from survey report						Source of survey values	Explanation of Differences
	CPR	mCPR	tCPR	Unmet need	Demand Satisfied	Intention to Use	CPR	mCPR	tCPR	Unmet need	Demand Satisfied	Intention to Use		
Kenya 2018 PMA2020 Round 7	45.3	43.5	1.8	13.0	77.0	66.3	46.1 (+0.8)	44.6 (+1.1)	1.5 (-0.3)	11.5 (-1.5)	77.4 (+0.4)		https://datalab.pmadata.org/<br><br>https://www.pmadata.org/countries/kenya	*Contraception*: PMA2020 considers women not currently using contraception, but who used emergency contraceptives in the last 12 months as current contraception users, we only consider emergency contraceptives a method contraception if it is recorded as a current method; reported LAM is considered a modern method only if most recent birth was in the last 6 months and last reported menses was before that birth, we consider LAM a traditional method of contraception.<br>*Need*: PMA2020 calculates PPA for births <24 months, we calculate using ≤24 months; infecundity calculations for last birth in 5 years calculated as ≥60 months, we use >60 months; only menopause is accepted as a reason for infecundity from reason_no_contra, we accept menopause, hysterectomy, infertile, and other text entry options.
Kenya 2019 PMA Phase 1	45.1	42.0	3.1	14.9	73.8	65.6	45.7 (+0.6)	43.0 (+1)	2.7 (-0.4)	12.1 (-2.8)	74.9 (+1.1)	17.1 (-48.5)	https://datalab.pmadata.org/<br><br>https://www.pmadata.org/countries/kenya	*Contraception*: PMA considers women not currently using contraception, but who used emergency contraceptives in the last 12 months as current contraception users, we only consider emergency contraceptives a method contraception if it is recorded as a current method; reported LAM is considered a modern method only if most recent birth was in the last 6 months and last reported menses was before that birth, we consider LAM a traditional method of contraception; SDM is considered a modern method of contraception, we consider it a traditional method of contraception.<br>*Need*: PMA calculates PPA for births <24 months, we calculate using ≤24 months; infecundity calculations for last birth in 5 years calculated as ≥60 months, we use >60 months; only menopause is accepted as a reason for infecundity from reason_no_contra, we accept menopause, hysterectomy, infertile, and other text entry options.<br>*Intent*: PMA calculates intent for women who are not pregnant and intend to use contraception within 12 months, we calculate intent for all women with no time limit for future intent use.

Survey*	Calculated values						Values from survey report						Source of survey values	Explanation of Differences
	CPR	mCPR	tCPR	Unmet need	Demand Satisfied	Intention to Use	CPR	mCPR	tCPR	Unmet need	Demand Satisfied	Intention to Use		
Kenya 2020 PMA Phase 2	49.6	45.2	4.4	14.4	75.8	66.8	49.8 (+0.2)	46.2 (+1)	3.6 (-0.8)	10.4 (-4)	76.9 (+1.1)	16.4 (-50.4)	https://datalab.pmadata.org/ https://www.pmadata.org/countries/kenya	*Contraception*: PMA considers women not currently using contraception, but who used emergency contraceptives in the last 12 months as current contraception users, we only consider emergency contraceptives a method contraception if it is recorded as a current method; reported LAM is considered a modern method only if most recent birth was in the last 6 months and last reported menses was before that birth, we consider LAM a traditional method of contraception; SDM is considered a modern method of contraception, we consider it a traditional method of contraception. *Need*: PMA calculates PPA for births <24 months, we calculate using ≤24 months; infecundity calculations for last birth in 5 years calculated as ≥60 months, we use >60 months; only menopause is accepted as a reason for infecundity from reason_no_contra, we accept menopause, hysterectomy, infertile, and other text entry options. *Intent*: PMA calculates intent for women who are not pregnant and intend to use contraception within 12 months, we calculate intent for all women with no time limit for future intent use.
Nigeria 1999 Multiple Indicator Cluster Survey	9.9	7.1	2.9	59.2	10.7		8.6 (-1.3)	6.0 (-1.1)	2.6 (-0.3)				https://mics.unicef.org/surveys	MICS only calculates indicators for married/in-union women, we calculate for all women.
Nigeria 2003 Demographic and Health Survey	13.3	8.0	5.3	20.6	27.9	45.5	13.3 (-0)	8.9 (+0.9)	4.4 (-0.9)	14.6 (-6)	32.0 (+4.1)	27.4 (-18.1)	https://www.statcompiler.com/en/	*Contraception*: DHS considers LAM and SDM modern methods of contraception, we consider them traditional methods of contraception. *Need*: DHS calculates PPA as <24 months, we calculate as ≤24 months; Only menopause is accepted as a reason for infecundity from reason_no_contra, we accept menopause, hysterectomy, infertile, and other text entry options. *Intent*: DHS only calculates intent for married/in-union women, we calculate for all women.

Survey*	Calculated values						Values from survey report						Source of survey values	Explanation of Differences
	CPR	mCPR	tCPR	Unmet need	Demand Satisfied	Intention to Use	CPR	mCPR	tCPR	Unmet need	Demand Satisfied	Intention to Use		
Nigeria 2005 Reproductive Health, Child Health, and Education Household, School, and Health Facility Baseline Survey	11.8	8.8	3.0	27.7	24.1	15.7		8.5 (-0.3)					https://www.measureevaluation.org/resources/publications/	*Contraception*: MEASURE doesn't include currently pregnant women in modern contraception use calculation, we include all women in the calculation. MEASURE does not consider emergency contraception a modern method of contraception, we consider it a modern method.
Nigeria 2007 General Household Survey	8.2	6.0	2.2	68.4	8.1									
Nigeria 2007 Multiple Indicator Cluster Survey	14.6	10.0	4.5	36.5	21.6	0.0	14.7 (+0.1)	9.1 (-0.9)	5.5 (+1)	19.7 (-16.8)	42.7 (+21.1)		https://mics.unicef.org/surveys	MICS only calculates indicators for married/in-union women, we calculate for all women.<br>*Need*: MICS does not include PPA in need calculations, we calculate PPA within the past 24 months and designate these women as not in need of contraception unless they did not desire this last pregnancy and current PPA status; fecundity determined based on response, not calculated, we calculate using information captured regarding length of cohabitation, ever using contraception, and lack of children.

Survey*	Calculated values						Values from survey report						Source of survey values	Explanation of Differences
	CPR	mCPR	tCPR	Unmet need	Demand Satisfied	Intention to Use	CPR	mCPR	tCPR	Unmet need	Demand Satisfied	Intention to Use		
Nigeria 2007 National AIDS and Reproductive Health Survey	13.4	9.7	3.8	24.2	28.6	6.3	13.4 (-0)	9.7 (-0)	3.7 (-0.1)			13.4 (+7.1)	National HIV/AIDS and Reproductive Health Survey (NARHS Plus, 2007). Federal Republic of Nigeria. December, 2008.	*Intent*: NARHS calculates intent for women who are not using modern methods of contraception, we calculate for women not using any method of contraception.
Nigeria 2007 Reproductive Health, Child Health, and Education Household, School, and Health Facility Survey	26.5	24.3	2.2	45.6	34.7	10.8		24.8 (+0.5)					https://www.measureevaluation.org/resources/publications/	*Contraception*: MEASURE doesn't include currently pregnant women in modern contraception use calculation, we calculate for all women. MEASURE does not consider emergency contraception a modern method of contraception, we consider it a modern method of contraception.

Survey*	Calculated values						Values from survey report						Source of survey values	Explanation of Differences
	CPR	mCPR	tCPR	Unmet need	Demand Satisfied	Intention to Use	CPR	mCPR	tCPR	Unmet need	Demand Satisfied	Intention to Use		
Nigeria 2008 Demographic and Health Survey	15.4	9.4	6.1	23.4	28.6	29.2	15.4 (-0)	10.5 (+1.1)	4.9 (-1.2)	16.1 (-7.3)	33.2 (+4.6)	20.9 (-8.3)	https://www.measureevaluation.org/resources/publications/	*Contraception*: DHS considers LAM and SDM modern methods of contraception, we consider them traditional methods of contraception. *Need*: DHS calculates PPA as <24 months, we calculate as ≤24 months; Only menopause is accepted as a reason for infecundity from reason_no_contra, we accept menopause, hysterectomy, infertile, and other text entry options. *Intent*: DHS only calculates intent for married/in-union women, we calculate for all women.
Nigeria 2009 Reproductive Health, Child Health, and Education Household, School, and Health Facility Survey	11.8	9.8	1.9	29.1	25.2	16.4		11.8 (+2)					https://www.measureevaluation.org/resources/publications/	*Contraception*: MEASURE doesn't include currently pregnant women in modern contraception use calculation, we calculate for all women. MEASURE does not consider emergency contraception a modern method of contraception, we consider it as a modern method of contraception.
Nigeria 2010 General Household Survey	9.1	7.9	1.2	53.4	12.9									
Nigeria 2011 Multiple Indicator Cluster Survey	18.3	11.8	6.5	27.7	29.9		17.5 (-0.8)	10.5 (-1.3)	7.0 (+0.5)	19.4 (-8.3)	47.7 (+17.8)		https://mics.unicef.org/surveys	MICS only calculates indicators for married/in-union women, we calculate for all women. *Need*: MICS calculates infecundity for women with no period >6 months, we use ≥6 months; 5 year timespan since last birth for married women calculated as ≥60 months, we use >60 months; timing between last birth and last menstrual period not used in PPA calculation, we calculate timing between last birth and last menstrual period to determine PPA status.

Survey*	Calculated values						Values from survey report						Source of survey values	Explanation of Differences
	CPR	mCPR	tCPR	Unmet need	Demand Satisfied	Intention to Use	CPR	mCPR	tCPR	Unmet need	Demand Satisfied	Intention to Use		
Nigeria 2012 General Household Survey	12.6	9.6	3.0	48.5	16.5									
Nigeria 2013 Demographic and Health Survey	16.1	10.8	5.3	18.5	36.9	34.6	16.0 (-0.1)	11.1 (+0.3)	4.8 (-0.5)	12.7 (-5.8)	38.8 (+1.9)	23.3 (-11.3)	https://www.statcompiler.com/en/	*Contraception*: DHS considers LAM and SDM modern methods of contraception, we consider both traditional methods of contraception. *Need*: DHS calculates PPA as <24 months, we calculate as ≤24 months; Only menopause is accepted as a reason for infecundity from reason_no_contra, we accept menopause, hysterectomy, infertile, and other text entry options. *Intent*: DHS only calculates intent for married/in-union women, we calculate for all women.
Nigeria (Kaduna) 2014 PMA2020 Round 1	9.0	8.5	0.5	30.9	21.5	40.0	8.7 (-0.3)	8.5 (-0)	0.3 (-0.2)	28.2 (-2.7)	23.0 (+1.5)		https://datalab.pmadata.org/ https://www.pmadata.org/countries/nigeria	*Contraception*: PMA2020 considers women not currently using contraception, but who used emergency contraceptives in the last 12 months as current contraception users, we only consider emergency contraceptives a method contraception if it is recorded as a current method; LAM and SDM are considered modern methods of contraception, we consider them traditional methods of contraception; "Don't know" responses to current use considered using a method of contraception, we consider this not using a method of contraception. *Intent*: PMA2020 calculates PPA for births <24 months, we calculate using ≤24 months; PPA only calculated using entered response, we calculate using timing between most recent birth and last menses; infecundity calculations for last birth in 5 years calculated as ≥60 months, we use >60 months; only menopause is accepted as a reason for infecundity from reason_no_contra, we accept menopause, hysterectomy, infertile, and other text entry options.

Survey*	Calculated values						Values from survey report						Source of survey values	Explanation of Differences
	CPR	mCPR	tCPR	Unmet need	Demand Satisfied	Intention to Use	CPR	mCPR	tCPR	Unmet need	Demand Satisfied	Intention to Use		
Nigeria (Lagos) 2014 PMA2020 Round 1	17.4	15.7	1.7	22.4	41.2	50.3	18.1 (+0.7)	16.7 (+1)	1.4 (-0.3)	19.2 (-3.2)	44.8 (+3.6)		https://datalab.pmadata.org/ https://www.pmadata.org/countries/nigeria	*Contraception*: PMA2020 considers women not currently using contraception, but who used emergency contraceptives in the last 12 months as current contraception users, we only consider emergency contraceptives a method contraception if it is recorded as a current method; LAM and SDM are considered modern methods of contraception, we consider them traditional methods of contraception; "Don't know" responses to current use considered using a method of contraception, we consider this not using a method of contraception. *Need*: PMA2020 calculates PPA for births <24 months, we calculate using ≤24 months; PPA only calculated using entered response, we calculate using timing between most recent birth and last menses; infecundity calculations for last birth in 5 years calculated as ≥60 months, we use >60 months; only menopause is accepted as a reason for infecundity from reason_no_contra, we accept menopause, hysterectomy, infertile, and other text entry options.
Nigeria (Kaduna) 2015 PMA2020 Round 2	14.4	13.0	1.4	26.8	32.6	47.4	14.5 (+0.1)	13.2 (+0.2)	1.3 (-0.1)	24.9 (-1.9)	33.5 (+0.9)		https://datalab.pmadata.org/ https://www.pmadata.org/countries/nigeria	*Contraception*: PMA2020 considers women not currently using contraception, but who used emergency contraceptives in the last 12 months as current contraception users, we only consider emergency contraceptives a method contraception if it is recorded as a current method; reported LAM is considered a modern method only if most recent birth was in the last 6 months and last reported menses was before that birth, we consider it a traditional method of contraception. *Need*: PMA2020 calculates PPA for births <24 months, we calculate using ≤24 months; infecundity calculations for last birth in 5 years calculated as ≥60 months, we use >60 months; only menopause is accepted as a reason for infecundity from reason_no_contra, we accept menopause, hysterectomy, infertile, and other text entry options.

Survey*	Calculated values						Values from survey report						Source of survey values	Explanation of Differences
	CPR	mCPR	tCPR	Unmet need	Demand Satisfied	Intention to Use	CPR	mCPR	tCPR	Unmet need	Demand Satisfied	Intention to Use		
Nigeria (Lagos) 2015 PMA2020 Round 2	27.2	20.0	7.2	24.2	45.2	53.8	27.8 (+0.6)	21.0 (+1)	6.7 (-0.5)	17.8 (-6.4)	46.1 (+0.9)		https://datalab.pmadata.org/ https://www.pmadata.org/countries/nigeria	*Contraception*: PMA2020 considers women not currently using contraception, but who used emergency contraceptives in the last 12 months as current contraception users, we only consider emergency contraceptives a method contraception if it is recorded as a current method; reported LAM is considered a modern method only if most recent birth was in the last 6 months and last reported menses was before that birth, we consider it a traditional method of contraception. *Need*: PMA2020 calculates PPA for births <24 months, we calculate using ≤24 months; infecundity calculations for last birth in 5 years calculated as ≥60 months, we use >60 months; only menopause is accepted as a reason for infecundity from reason_no_contra, we accept menopause, hysterectomy, infertile, and other text entry options.
Nigeria (National) 2016 PMA2020 Round 3	17.9	14.1	3.9	26.6	34.6	46.2	19.8 (+1.9)	14.9 (+0.8)	4.9 (+1)	20.4 (-6.2)	37.1 (+2.5)		https://datalab.pmadata.org/ https://www.pmadata.org/countries/nigeria	*Contraception*: PMA2020 considers women not currently using contraception, but who used emergency contraceptives in the last 12 months as current contraception users, we only consider emergency contraceptives a method contraception if it is recorded as a current method; LAM is not considered a method of contraception, we consider it a traditional method of contraception. *Need*: PMA2020 calculates PPA for births <24 months, we calculate using ≤24 months; infecundity calculations for last birth in 5 years calculated as ≥60 months, we use >60 months; only menopause is accepted as a reason for infecundity from reason_no_contra, we accept menopause, hysterectomy, infertile, and other text entry options.
Nigeria 2016–2017 Multiple Indicator Cluster Survey	11.8	9.2	2.6	25.6	26.4		13.4 (+1.6)	10.8 (+1.6)	2.6 (-0)	27.6 (+2)	32.7 (+6.3)		https://mics.unicef.org/surveys	MICS only calculates indicators for married/in-union women, we calculate for all women. *Contraception*: MICS considers LAM and SDM modern methods of contraception, we consider them traditional methods of contraception. *Need*: MICS calculates infecundity for women with no period >6 months, we use ≥6 months; 5 year timespan since last birth for married women calculated as ≥60 months, we use >60 months.

Survey*	Calculated values						Values from survey report						Source of survey values	Explanation of Differences
	CPR	mCPR	tCPR	Unmet need	Demand Satisfied	Intention to Use	CPR	mCPR	tCPR	Unmet need	Demand Satisfied	Intention to Use		
Nigeria (National) 2017 PMA2020 Round 4	20.8	14.2	6.6	26.3	35.2	52.4	21.0 (+0.2)	14.7 (+0.5)	6.2 (-0.4)	19.0 (-7.3)	36.8 (+1.6)		https://datalab.pmadata.org/ https://www.pmadata.org/countries/nigeria	*Contraception*: PMA2020 considers women not currently using contraception, but who used emergency contraceptives in the last 12 months as current contraception users, we only consider emergency contraceptives a method contraception if it is recorded as a current method; reported LAM is considered a modern method only if most recent birth was in the last 6 months and last reported menses was before that birth, we consider it a traditional method of contraception. *Need*: PMA2020 calculates PPA for births <24 months, we calculate using ≤24 months; infecundity calculations for last birth in 5 years calculated as ≥60 months, we use >60 months; only menopause is accepted as a reason for infecundity from reason_no_contra, we accept menopause, hysterectomy, infertile, and other text entry options.
Nigeria (Oyo) 2017 PMA2020 Round 1	27.6	24.0	3.6	19.2	55.5	60.9	27.8 (+0.2)	24.5 (+0.5)	3.3 (-0.3)	15.2 (-4)	57.0 (+1.5)		https://datalab.pmadata.org/ https://www.pmadata.org/countries/nigeria	*Contraception*: PMA2020 considers women not currently using contraception, but who used emergency contraceptives in the last 12 months as current contraception users, we only consider emergency contraceptives a method contraception if it is recorded as a current method; LAM is not considered a method of contraception, we consider it a traditional method of contraception. *Need*: PMA2020 calculates PPA for births <24 months, we calculate using ≤24 months; infecundity calculations for last birth in 5 years calculated as ≥60 months, we use >60 months; only menopause is accepted as a reason for infecundity from reason_no_contra, we accept menopause, hysterectomy, infertile, and other text entry options.

Survey*	Calculated values						Values from survey report						Source of survey values	Explanation of Differences
	CPR	mCPR	tCPR	Unmet need	Demand Satisfied	Intention to Use	CPR	mCPR	tCPR	Unmet need	Demand Satisfied	Intention to Use		
Nigeria (National) 2018 PMA2020 Round 5	23.8	16.8	7.0	24.0	41.1	51.3	24.2 (+0.4)	17.6 (+0.8)	6.6 (-0.4)	16.6 (-7.4)	43.1 (+2)		https://datalab.pmadata.org/ https://www.pmadata.org/countries/nigeria	*Contraception*: PMA2020 considers women not currently using contraception, but who used emergency contraceptives in the last 12 months as current contraception users, we only consider emergency contraceptives a method contraception if it is recorded as a current method; reported LAM is considered a modern method only if most recent birth was in the last 6 months and last reported menses was before that birth, we consider it a traditional method of contraception. *Need*: PMA2020 calculates PPA for births <24 months, we calculate using ≤24 months; infecundity calculations for last birth in 5 years calculated as ≥60 months, we use >60 months; only menopause is accepted as a reason for infecundity from reason_no_contra, we accept menopause, hysterectomy, infertile, and other text entry options.
Nigeria 2018 HIV & AIDS Indicator and Impact Survey	9.8	8.7	1.1	50	14.8									
Nigeria 2018 Demographic and Health Survey	14.3	9.7	4.7	20.3	32.2	46.2	14.3 (-0)	10.5 (+0.8)	3.8 (-0.9)	15.2 (-5.1)	35.7 (+3.5)	35.2 (-11)	https://www.statcompiler.com/en/	*Contraception*: DHS considers LAM and SDM modern methods of contraception, we consider them traditional methods of contraception. *Need*: DHS calculates PPA as <24 months, we calculate as ≤24 months; Only menopause is accepted as a reason for infecundity from reason_no_contra, we accept menopause, hysterectomy, infertile, and other text entry options. *Intent*: DHS only calculates intent for married/in-union women, we calculate for all women.

Survey*	Calculated values						Values from survey report						Source of survey values	Explanation of Differences
	CPR	mCPR	tCPR	Unmet need	Demand Satisfied	Intention to Use	CPR	mCPR	tCPR	Unmet need	Demand Satisfied	Intention to Use		
Nigeria (Kano) 2020 PMA Phase 1	9.4	8.0	1.4	23.7	25.1	43.4	9.4 (-0)	8.1 (+0.1)	1.3 (-0.1)	21.6 (-2.1)	26.1 (+1)	12.4 (-31)	https://www.pmadata.org/countries/nigeria	*Contraception*: PMA considers women not currently using contraception, but who used emergency contraceptives in the last 12 months as current contraception users, we only consider emergency contraceptives a method contraception if it is recorded as a current method; reported LAM is considered a modern method only if most recent birth was in the last 6 months and last reported menses was before that birth, we consider LAM a traditional method of contraception; SDM is considered a modern method of contraception, we consider it a traditional method of contraception.<br>*Need*: PMA calculates PPA for births <24 months, we calculate using ≤24 months; infecundity calculations for last birth in 5 years calculated as ≥60 months, we use >60 months; only menopause is accepted as a reason for infecundity from reason_no_contra, we accept menopause, hysterectomy, infertile, and other text entry options.<br>*Intent*: PMA calculates intent for women who are not pregnant and intend to use contraception within 12 months, we calculate for all women with no time limit for future intent use.

Survey*	Calculated values						Values from survey report						Source of survey values	Explanation of Differences
	CPR	mCPR	tCPR	Unmet need	Demand Satisfied	Intention to Use	CPR	mCPR	tCPR	Unmet need	Demand Satisfied	Intention to Use		
Nigeria (Lagos) 2020 PMA Phase 1	37.2	24.1	13.1	23.7	50.4	53.9	38.2 (+1)	25.4 (+1.3)	12.8 (-0.3)	10.8 (-12.9)	52.4 (+2)	7.9 (-46)	https://www.pmadata.org/countries/nigeria	*Contraception*: PMA considers women not currently using contraception, but who used emergency contraceptives in the last 12 months as current contraception users, we only consider emergency contraceptives a method contraception if it is recorded as a current method; reported LAM is considered a modern method only if most recent birth was in the last 6 months and last reported menses was before that birth, we consider LAM a traditional method of contraception; SDM is considered a modern method of contraception, we consider it a traditional method of contraception.<br>*Need*: PMA calculates PPA for births <24 months, we calculate using ≤24 months; infecundity calculations for last birth in 5 years calculated as ≥60 months, we use >60 months; only menopause is accepted as a reason for infecundity from reason_no_contra, we accept menopause, hysterectomy, infertile, and other text entry options.<br>*Intent*: PMA calculates intent for women who are not pregnant and intend to use contraception within 12 months, we calculate for all women with no time limit for future intent use.

Survey*	Calculated values						Values from survey report						Source of survey values	Explanation of Differences
	CPR	mCPR	tCPR	Unmet need	Demand Satisfied	Intention to Use	CPR	mCPR	tCPR	Unmet need	Demand Satisfied	Intention to Use		
Nigeria (Kano) 2020 PMA Phase 2	9.6	7.4	2.1	24.5	23.2	39.3	9.6 (-0)	7.5 (+0.1)	2.1 (-0)	20.7 (-3.8)	24.7 (+1.5)	10.6 (-28.7)	https://www.pmadata.org/countries/nigeria	*Contraception*: PMA considers women not currently using contraception, but who used emergency contraceptives in the last 12 months as current contraception users, we only consider emergency contraceptives a method contraception if it is recorded as a current method; reported LAM is considered a modern method only if most recent birth was in the last 6 months and last reported menses was before that birth, we consider LAM a traditional method of contraception; SDM is considered a modern method of contraception, we consider it a traditional method of contraception. *Need*: PMA calculates PPA for births <24 months, we calculate using ≤24 months; infecundity calculations for last birth in 5 years calculated as ≥60 months, we use >60 months; only menopause is accepted as a reason for infecundity from reason_no_contra, we accept menopause, hysterectomy, infertile, and other text entry options. *Intent*: PMA calculates intent for women who are not pregnant and intend to use contraception within 12 months, we calculate for all women with no time limit for future intent use.

Survey*	Calculated values						Values from survey report						Source of survey values	Explanation of Differences
	CPR	mCPR	tCPR	Unmet need	Demand Satisfied	Intention to Use	CPR	mCPR	tCPR	Unmet need	Demand Satisfied	Intention to Use		
Nigeria (Lagos) 2020 PMA Phase 2	39.9	23.5	16.4	27.0	46.6	45.5	40.1 (+0.2)	24.2 (+0.7)	15.9 (-0.5)	10.6 (-16.4)	47.8 (+1.2)	6.7 (-38.8)	https://www.pmadata.org/countries/nigeria	*Contraception*: PMA considers women not currently using contraception, but who used emergency contraceptives in the last 12 months as current contraception users, we only consider emergency contraceptives a method contraception if it is recorded as a current method; reported LAM is considered a modern method only if most recent birth was in the last 6 months and last reported menses was before that birth, we consider LAM a traditional method of contraception; SDM is considered a modern method of contraception, we consider it a traditional method of contraception. *Need*: PMA calculates PPA for births <24 months, we calculate using ≤24 months; infecundity calculations for last birth in 5 years calculated as ≥60 months, we use >60 months; only menopause is accepted as a reason for infecundity from reason_no_contra, we accept menopause, hysterectomy, infertile, and other text entry options. *Intent*: PMA calculates intent for women who are not pregnant and intend to use contraception within 12 months, we calculate for all women with no time limit for future intent use.

*Surveys included in indicator calculation where microdata are available.

Table S2: Surveys included in this analysis

Survey	NID*	Citation	Geographic Detail	Sample size (women age 15–49)**	Gold Standard / Crosswalk		
					Contraceptive Use	Need	Intention to Use
Burkina Faso 1998 Demographic and Health Survey	19076	Macro International, Inc, National Institute of Statistics and Demography (Burkina Faso). Burkina Faso Demographic and Health Survey 1998–1999. Fairfax, United States of America: ICF International.	Point	6445	Gold Standard	Gold Standard	Gold Standard
Burkina Faso 2003 Demographic and Health Survey	19088	Macro International, Inc, National Institute of Statistics and Demography (Burkina Faso). Burkina Faso Demographic and Health Survey 2003. Fairfax, United States of America: ICF International.	Point	12477	Gold Standard	Gold Standard	Gold Standard
Burkina Faso 2006 Multiple Indicator Cluster Survey	1927	National Institute of Statistics and Demography (Burkina Faso), United Nations Children's Fund (UNICEF). Burkina Faso Multiple Indicator Cluster Survey 2006. New York, United States of America: United Nations Children's Fund (UNICEF).	Point	7316	Gold Standard	Crosswalk 8	-
Burkina Faso 2010 Demographic and Health Survey	19133	ICF Macro, Ministry of Health (Burkina Faso), National Institute of Statistics and Demography (Burkina Faso). Burkina Faso Demographic and Health Survey 2010–2011. Fairfax, United States of America: ICF International.	Point	17087	Gold Standard	Gold Standard	Gold Standard

Survey	NID*	Citation	Geographic Detail	Sample size (women age 15–49)**	Gold Standard / Crosswalk		
					Contraceptive Use	Need	Intention to Use
Burkina Faso 2014 PMA2020 Round 1	257044	Burkina Faso Institut national de la statistique et de la démographie (National Institute of Statistics and Demography) and The Bill & Melinda Gates Institute for Population and Reproductive Health at The Johns Hopkins Bloomberg School of Public Health. Performance Monitoring and Accountability 2020 (PMA2020) Survey round 1, PMA2014/Burkina Faso-R1. 2014. Ouagadougou, Burkina Faso and Baltimore, Maryland, USA.	Polygon-Admin 1	2066	Gold Standard	Gold Standard	Gold Standard
Burkina Faso 2015 PMA2020 Round 2	257045	Burkina Faso Institut national de la statistique et de la démographie (National Institute of Statistics and Demography), and The Bill & Melinda Gates Institute for Population and Reproductive Health at The Johns Hopkins Bloomberg School of Public Health. Performance Monitoring and Accountability 2020 (PMA2020) Survey round 2, PMA2015/Burkina Faso-R2. 2015. Ouagadougou, Burkina Faso and Baltimore, Maryland, USA.	Polygon-Admin 1	2112	Gold Standard	Gold Standard	Gold Standard
Burkina Faso 2016 PMA2020 Round 3	285993	Burkina Faso Institut national de la statistique et de la démographie (National Institute of Statistics and Demography), and The Bill & Melinda Gates Institute for Population and Reproductive Health at The Johns Hopkins Bloomberg School of Public Health. Performance Monitoring and Accountability 2020 (PMA2020) Survey round 3, PMA2016/Burkina Faso-R3. 2016.	Polygon-Admin 1	3271	Gold Standard	Gold Standard	Gold Standard

Survey	NID*	Citation	Geographic Detail	Sample size (women age 15–49)**	Gold Standard / Crosswalk		
					Contraceptive Use	Need	Intention to Use
		Ouagadougou, Burkina Faso and Baltimore, Maryland, USA.					
Burkina Faso 2016–2017 PMA2020 Round 4	307751	Burkina Faso Institut national de la statistique et de la démographie (National Institute of Statistics and Demography), and The Bill & Melinda Gates Institute for Population and Reproductive Health at The Johns Hopkins Bloomberg School of Public Health. Performance Monitoring and Accountability 2020 (PMA2020) Survey round 4, PMA2016/Burkina Faso-R4. 2016. Ouagadougou, Burkina Faso and Baltimore, Maryland, USA.	Polygon-Admin 1	3220	Gold Standard	Gold Standard	Gold Standard
Burkina Faso 2017–2018 PMA2020 Round 5	375719	Institut Supérieur des Sciences de la Population, Université Joseph Ki-Zerbo, Ouagadougou, Burkina Faso, and The Bill &Melinda Gates Institute for Population and Reproductive Health at The Johns Hopkins Bloomberg School of Public Health. Performance Monitoring and Accountability 2020 (PMA2020) Survey Round 5, PMA2017/Burkina Faso-R5. 2017. Ouagadougou, Burkina Faso and Baltimore, Maryland, USA.	Point	3512	Gold Standard	Gold Standard	Gold Standard

Survey	NID*	Citation	Geographic Detail	Sample size (women age 15–49)**	Gold Standard / Crosswalk		
					Contracepti ve Use	Need	Intention to Use
Burkina Faso 2018–2019 PMA2020 Round 6	407829	Burkina Faso Institut national de la statistique et de la démographie (National Institute of Statistics and Demography), and The Bill & Melinda Gates Institute for Population and Reproductive Health at The Johns Hopkins Bloomberg School of Public Health. Performance Monitoring and Accountability 2020 (PMA2020) Survey Round 6, PMA2018/Burkina Faso-R6. 2018. Ouagadougou, Burkina Faso and Baltimore, Maryland, USA.	Point	3329	Gold Standard	Gold Standard	Gold Standard
Burkina Faso 2020 PMA Phase 1	470507	Bill and Melinda Gates Institute for Population and Reproductive Health, Johns Hopkins Bloomberg School of Public Health, Higher Institute of Population Sciences (ISSP) (Burkina Faso), Jhpiego. Burkina Faso Performance Monitoring and Action Phase 1 Household and Female Survey 2019–2020. Baltimore, United States of America: Johns Hopkins Bloomberg School of Public Health, 2021.	Point	6590	Gold Standard	Gold Standard	Gold Standard
Burkina Faso 2020 PMA Phase 2	493322	Bill and Melinda Gates Institute for Population and Reproductive Health, Johns Hopkins Bloomberg School of Public Health, Higher Institute of Population Sciences (ISSP) (Burkina Faso), Jhpiego. Burkina Faso Performance Monitoring and Action Phase 2 Household and Female Survey 2020–2021. Baltimore, United States of America: Johns Hopkins Bloomberg School of Public Health, 2021.	Point	6362	Gold Standard	Gold Standard	Gold Standard

Survey	NID*	Citation	Geographic Detail	Sample size (women age 15–49)**	Gold Standard / Crosswalk		
					Contraceptive Use	Need	Intention to Use
Kenya 1998 Demographic and Health Survey	20132	Central Bureau of Statistics (Kenya), Macro International, Inc, National Council for Population Development (NCPD). Kenya Demographic and Health Survey 1998. Fairfax, United States of America: ICF International.	Polygon-Admin 1	7881	Gold Standard	Gold Standard	Gold Standard
Kenya 2003 Demographic and Health Survey	20145	Centers for Disease Control and Prevention (CDC), Central Bureau of Statistics (Kenya), Macro International, Inc, Ministry of Health (Kenya), National Council for Population and Development (Kenya). Kenya Demographic and Health Survey 2003. Fairfax, United States of America: ICF International.	Point	8195	Gold Standard	Gold Standard	Gold Standard
Kenya 2007 AIDS Indicator Survey	133219	Centers for Disease Control and Prevention (CDC), Kenya Medical Research Institute (KEMRI), Kenya National Bureau of Statistics, Ministry of Public Health and Sanitation (Kenya), National AIDS Control Council (Kenya), National AIDS and STI Control Programme (NASCOP) (Kenya), National Coordinating Agency for Population and Development (Kenya), National Public Health Laboratory Services, Ministry of Public Health and Sanitation (Kenya), United States Agency for International Development (USAID). Kenya AIDS Indicator Survey 2007. Nairobi, Kenya: Kenya National Bureau of Statistics.	Point	8901	Gold Standard	Crosswalk 7	-

Survey	NID*	Citation	Geographic Detail	Sample size (women age 15–49)**	Gold Standard / Crosswalk		
					Contraceptive Use	Need	Intention to Use
Kenya 2007 Multiple Indicator Cluster Survey (Eastern Province)	155335	Kenya National Bureau of Statistics, United Nations Children's Fund (UNICEF). Kenya - North Eastern Province Multiple Indicator Cluster Survey 2007. Nairobi, Kenya: Kenya National Bureau of Statistics.	Point	888	Gold Standard	Crosswalk 10	-
Kenya 2008 Multiple Indicator Cluster Survey (Eastern Province)	7401	Kenya National Bureau of Statistics, United Nations Children's Fund (UNICEF). Kenya - Eastern Province Multiple Indicator Cluster Survey 2008. Nairobi, Kenya: Kenya National Bureau of Statistics.	Point	14910	Gold Standard	Crosswalk 11	-
Kenya 2008–2009 Demographic and Health Survey	21365	ICF Macro, Kenya Medical Research Institute (KEMRI), Kenya National Bureau of Statistics, Ministry of Public Health and Sanitation (Kenya), National AIDS and STI Control Programme (NASCOP) (Kenya), National Aids Control Council (NACC), National Coordinating Agency for Population and Development (Kenya). Kenya Demographic and Health Survey 2008–2009. Fairfax, United States of America: ICF International.	Point	8444	Gold Standard	Gold Standard	Gold Standard
Kenya 2010 Urban Reproductive Health Initiative	165740	Kenya National Bureau of Statistics. Kenya Urban Reproductive Health Initiative Baseline Household Survey 2010.	Polygon-Admin 1	8932	Gold Standard	Crosswalk 4	Crosswalk 4
Kenya 2011 Multiple Indicator Cluster Survey (Nyanza Province)	135416	Kenya National Bureau of Statistics, United Nations Children's Fund (UNICEF). Kenya - Nyanza Province Multiple Indicator Cluster Survey 2011. Nairobi, Kenya: Kenya National Bureau of Statistics.	Point	5908	Gold Standard	Crosswalk 6	-

Survey	NID*	Citation	Geographic Detail	Sample size (women age 15–49)**	Gold Standard / Crosswalk		
					Contraceptive Use	Need	Intention to Use
Kenya 2012 AIDS Indicator Survey	133304	Kenya National Bureau of Statistics, Ministry of Devolution and Planning (Kenya), Ministry of Health (Kenya), National AIDS and STI Control Programme (NASCOP) (Kenya). Kenya AIDS Indicator Survey 2012–2013. Nairobi, Kenya: Kenya National Bureau of Statistics.	Point	6910	Gold Standard	Crosswalk 5	-
Kenya 2013 Multiple Indicator Cluster Survey (Bungoma County)	203654	Kenya National Bureau of Statistics, Population Studies and Research Institute, University of Nairobi (Kenya), United Nations Children's Fund (UNICEF). Kenya - Bungoma County Multiple Indicator Survey 2013–2014. New York, United States of America: United Nations Children's Fund (UNICEF), 2015.	Point	1213	Gold Standard	Gold Standard	-
Kenya 2013 Multiple Indicator Cluster Survey (Kakamega County)	203663	Kenya National Bureau of Statistics, Population Studies and Research Institute, University of Nairobi (Kenya), United Nations Children's Fund (UNICEF). Kenya - Kakamega County Multiple Indicator Survey 2013–2014. New York, United States of America: United Nations Children's Fund (UNICEF), 2015.	Point	998	Gold Standard	Gold Standard	-
Kenya 2013 Multiple Indicator Cluster Survey (Turkana County)	203664	Kenya National Bureau of Statistics, Population Studies and Research Institute, University of Nairobi (Kenya), United Nations Children's Fund (UNICEF). Kenya - Turkana County Multiple Indicator Survey 2013–2014. New York, United States of America: United Nations Children's Fund (UNICEF), 2015.	Point	1104	Gold Standard	Gold Standard	-

Survey	NID*	Citation	Geographic Detail	Sample size (women age 15–49)**	Gold Standard / Crosswalk		
					Contracepti ve Use	Need	Intention to Use
Kenya 2014 Demographic and Health Survey (long form)	157057	ICF International, Kenya Medical Research Institute (KEMRI), Kenya National Bureau of Statistics, Ministry of Health (Kenya), National AIDS Control Council (Kenya), National Council for Population and Development (Kenya). Kenya Demographic and Health Survey 2014. Fairfax, United States of America: ICF International.	Point	14741	Gold Standard	Gold Standard	Gold Standard
Kenya 2014 Demographic and Health Survey (short form)	157057	ICF International, Kenya Medical Research Institute (KEMRI), Kenya National Bureau of Statistics, Ministry of Health (Kenya), National AIDS Control Council (Kenya), National Council for Population and Development (Kenya). Kenya Demographic and Health Survey 2014. Fairfax, United States of America: ICF International.	Point	16338	Gold Standard	Crosswalk 9	-
Kenya 2014 PMA2020 Round 1	197910	International Centre for Reproductive Health Kenya (ICRHK) and The Bill & Melinda Gates Institute for Population and Reproductive Health at The Johns Hopkins Bloomberg School of Public Health. Performance Monitoring and Accountability 2020 (PMA2020) Survey round 1, PMA2014/Kenya-R1. 2014. Kenya and Baltimore, Maryland, USA.	Polygon-Admin 2	3781	Gold Standard	Gold Standard	Gold Standard

Survey	NID*	Citation	Geographic Detail	Sample size (women age 15–49)**	Gold Standard / Crosswalk		
					Contraceptive Use	Need	Intention to Use
Kenya 2014 PMA2020 Round 2	256338	Bill and Melinda Gates Institute for Population and Reproductive Health, Johns Hopkins Bloomberg School of Public Health, International Center for Reproductive Health (Kenya), Kenya National Bureau of Statistics, Ministry of Health (Kenya), National Council for Population and Development (Kenya). Kenya Performance Monitoring and Accountability 2020 Survey, Round 2 2014. Baltimore, United States of America: Johns Hopkins Bloomberg School of Public Health.	Polygon-Admin 2	4345	Gold Standard	Gold Standard	Gold Standard
Kenya 2015 PMA2020 Round 3	256365	International Centre for Reproductive Health Kenya (ICRHK) and The Bill & Melinda Gates Institute for Population and Reproductive Health at The Johns Hopkins Bloomberg School of Public Health. Performance Monitoring and Accountability 2020 (PMA2020) Survey round 3, PMA2015/Kenya-R3. 2015. Kenya and Baltimore, Maryland, USA.	Polygon-Admin 2	4403	Gold Standard	Gold Standard	Gold Standard
Kenya 2015 PMA2020 Round 4	256366	International Centre for Reproductive Health Kenya (ICRHK) and The Bill & Melinda Gates Institute for Population and Reproductive Health at The Johns Hopkins Bloomberg School of Public Health. Performance Monitoring and Accountability 2020 (PMA2020) Survey round 4, PMA2015/Kenya-R4. 2015. Kenya and Baltimore, Maryland, USA.	Polygon-Admin 2	4929	Gold Standard	Gold Standard	Gold Standard

Survey	NID*	Citation	Geographic Detail	Sample size (women age 15–49)**	Gold Standard / Crosswalk		
					Contraceptive Use	Need	Intention to Use
Kenya 2016 PMA2020 Round 5	347047	International Centre for Reproductive Health Kenya (ICRHK) and The Bill & Melinda Gates Institute for Population and Reproductive Health at The Johns Hopkins Bloomberg School of Public Health. Performance Monitoring and Accountability 2020 (PMA2020) Survey round 5, PMA2016/Kenya-R5. 2016. Kenya and Baltimore, Maryland, USA.	Point	5896	Gold Standard	Gold Standard	Gold Standard
Kenya 2017 PMA2020 Round 6	374949	International Centre for Reproductive Health Kenya (ICRHK) and The Bill & Melinda Gates Institute for Population and Reproductive Health at The Johns Hopkins Bloomberg School of Public Health. Performance Monitoring and Accountability 2020 (PMA2020) Survey Round 6, PMA2017/Kenya-R6. 2017. Kenya and Baltimore, Maryland, USA.	Point	5877	Gold Standard	Gold Standard	Gold Standard
Kenya 2018 PMA2020 Round 7	407837	International Centre for Reproductive Health Kenya (ICRHK) and The Bill & Melinda Gates Institute for Population and Reproductive Health at The Johns Hopkins Bloomberg School of Public Health. Performance Monitoring and Accountability 2020 (PMA2020) Survey Round 7, PMA2018/Kenya-R7. 2018. Kenya and Baltimore, Maryland, USA.	Point	5677	Gold Standard	Gold Standard	Gold Standard

Survey	NID*	Citation	Geographic Detail	Sample size (women age 15–49)**	Gold Standard / Crosswalk		
					Contraceptive Use	Need	Intention to Use
Kenya 2019 PMA Phase 1	470504	Bill and Melinda Gates Institute for Population and Reproductive Health, Johns Hopkins Bloomberg School of Public Health, International Center for Reproductive Health (Kenya), Jhpiego. Kenya Performance Monitoring and Action Phase 1 Household and Female Survey 2019. Baltimore, United States of America: Johns Hopkins Bloomberg School of Public Health, 2021.	Point	9478	Gold Standard	Gold Standard	Gold Standard
Kenya 2020 PMA Phase 2	493363	Bill and Melinda Gates Institute for Population and Reproductive Health, Johns Hopkins Bloomberg School of Public Health, International Center for Reproductive Health (Kenya), Jhpiego, Kenya National Bureau of Statistics. Kenya Performance Monitoring and Action Phase 2 Household and Female Survey 2020. Baltimore, United States of America: Johns Hopkins Bloomberg School of Public Health, 2021.	Point	9309	Gold Standard	Gold Standard	Gold Standard
Nigeria 1999 Multiple Indicator Cluster Survey	9506	National Bureau of Statistics (Nigeria), United Nations Children's Fund (UNICEF). Nigeria Multiple Indicator Cluster Survey 1999. Abuja, Nigeria: National Bureau of Statistics (Nigeria).	Polygon-Admin 1	19979	Gold Standard	Crosswalk 12	-
Nigeria 1999 Demographic and Health Survey	20555	Macro International, Inc, National Population Commission of Nigeria. Nigeria Demographic and Health Survey 1999. Calverton, United States of America: Macro International, Inc.	Polygon-Admin 1	4197	Crosswalk 1	-	-

Survey	NID*	Citation	Geographic Detail	Sample size (women age 15–49)**	Gold Standard / Crosswalk		
					Contraceptive Use	Need	Intention to Use
Nigeria 2003 Demographic and Health Survey	20567	Department for International Development (DFiD) (United Kingdom), National Population Commission of Nigeria, ORC Macro, United Nations Children's Fund (UNICEF), United Nations Population Fund (UNFPA). Nigeria Demographic and Health Survey 2003. Fairfax, United States of America: ICF International.	Point	7620	Gold Standard	Gold Standard	Gold Standard
Nigeria 2003 National HIV and Reproductive Health Survey	133680	Federal Ministry of Health [Nigeria]. 2003. National HIV/AIDS and Reproductive Health Survey, 2003. Federal Ministry of Health Abuja, Nigeria.	Polygon-Admin 1	5128	Gold Standard	-	-
Nigeria 2005 Reproductive Health, Child Health, and Education Household, School, and Health Facility Baseline Survey	50393	MEASURE Evaluation Project, Carolina Population Center, University of North Carolina, Center for Research, Evaluation, and Resource Development (CRERD), Center for Communication Programs, Bloomberg School of Public Health, Johns Hopkins, Creative Associates International, Constella Futures, Adolescent Health and Information Project (Nigeria), Federation of Muslim Women's Associations of Nigeria (FOMWAN), Nigerian Medical Association, Management Sciences for Health (MSH), Civil Society Action Coalition on Education For All. Nigeria Reproductive Health, Child Health, and Education Household, School, and Health Facility Baseline Surveys 2005. Chapel Hill, United States: MEASURE Evaluation Project, Carolina Population Center, University of North Carolina.	Polygon-Admin 2	2136	Gold Standard	Crosswalk 3	-

Survey	NID*	Citation	Geographic Detail	Sample size (women age 15–49)**	Gold Standard / Crosswalk		
					Contraceptive Use	Need	Intention to Use
Nigeria 2005 National HIV and Reproductive Health Survey	217512	Federal Ministry of Health (Nigeria). Nigeria National HIV/AIDS and Reproductive Health Survey 2005.	Polygon-Admin 1	4689	Gold Standard	-	-
Nigeria 2007 Multiple Indicator Cluster Survey	9516	United Nations Children's Fund (UNICEF), National Bureau of Statistics (Nigeria). Nigeria Multiple Indicator Cluster Survey 2007. New York, United States: United Nations Children's Fund (UNICEF).	Polygon-Admin 1	24565	Gold Standard	Crosswalk 8	-
Nigeria 2007 Reproductive Health, Child Health, and Education Household, School, and Health Facility Survey	50426	MEASURE Evaluation Project, Carolina Population Center, University of North Carolina, Center for Research, Evaluation, and Resource Development (CRERD), Center for Communication Programs, Bloomberg School of Public Health, Johns Hopkins, Creative Associates International, Constella Futures, Adolescent Health and Information Project (Nigeria), Federation of Muslim Women's Associations of Nigeria (FOMWAN), Nigerian Medical Association, Management Sciences for Health (MSH), Civil Society Action Coalition on Education For All. Nigeria Reproductive Health, Child Health, and Education Household, School, and Health Facility Midline Surveys 2007. Chapel Hill, United States: MEASURE Evaluation Project, Carolina Population Center, University of North Carolina.	Polygon-Admin 2	2327	Gold Standard	Crosswalk 2	-

Survey	NID*	Citation	Geographic Detail	Sample size (women age 15–49)**	Gold Standard / Crosswalk		
					Contraceptive Use	Need	Intention to Use
Nigeria 2007 National AIDS and Reproductive Health Survey	325046	Federal Ministry of Health (Nigeria), National Agency for the Control of AIDS (NACA) (Nigeria), National Bureau of Statistics (Nigeria), National Population Commission (NPC), Nigerian Institute of Medical Research, Society for Family Health (Nigeria), University College Hospital, Ibadan. Nigeria National HIV/AIDS and Reproductive Health Survey 2007.	Polygon-Admin 1	5360	Gold Standard	-	-
Nigeria 2007 General Household Survey	24915	Central Bank of Nigeria, National Bureau of Statistics (Nigeria), Nigerian Communications Commission (NCC). Nigeria General Household Survey 2008.	Polygon-Admin 1	17902	Gold Standard	Crosswalk 12	-
Nigeria 2008 Demographic and Health Survey	21433	Macro International, Inc, National Population Commission of Nigeria. Nigeria Demographic and Health Survey 2008. Fairfax, United States of America: ICF International, 2009.	Point	33385	Gold Standard	Gold Standard	Gold Standard

Survey	NID*	Citation	Geographic Detail	Sample size (women age 15–49)**	Gold Standard / Crosswalk		
					Contraceptive Use	Need	Intention to Use
Nigeria 2009 Reproductive Health, Child Health, and Education Household, School, and Health Facility Survey	50441	MEASURE Evaluation Project, Carolina Population Center, University of North Carolina, Center for Research, Evaluation, and Resource Development (CRERD), Center for Communication Programs, Bloomberg School of Public Health, Johns Hopkins, Creative Associates International, Futures Group International, Adolescent Health and Information Project (Nigeria), Federation of Muslim Women's Associations of Nigeria (FOMWAN), Nigerian Medical Association, Management Sciences for Health (MSH), Civil Society Action Coalition on Education For All. Nigeria Reproductive Health, Child Health, and Education Household, School, and Health Facility End-of-Project Surveys. Chapel Hill, United States: MEASURE Evaluation Project, Carolina Population Center, University of North Carolina.	Polygon-Admin 2	2412	Gold Standard	Crosswalk 3	-
Nigeria 2010 General Household Survey	151802	National Bureau of Statistics (Nigeria). Nigeria General Household Survey 2010–2011. Abuja, Nigeria: National Bureau of Statistics (Nigeria).	Point	6480	Gold Standard	Crosswalk 12	-
Nigeria 2011 Multiple Indicator Cluster Survey	76703	National Bureau of Statistics (Nigeria), United Nations Children's Fund (UNICEF). Nigeria Multiple Indicator Cluster Survey 2011. New York, United States of America: United Nations Children's Fund (UNICEF), 2013.	Polygon-Admin 1	30772	Gold Standard	Crosswalk 2	-

Survey	NID*	Citation	Geographic Detail	Sample size (women age 15–49)**	Gold Standard / Crosswalk		
					Contraceptive Use	Need	Intention to Use
Nigeria 2012 General Household Survey	151797	National Bureau of Statistics (Nigeria). Nigeria General Household Survey, Panel 2012–2013, Wave 2. Washington DC, United States of America: World Bank.	Point	6407	Gold Standard	Crosswalk 12	-
Nigeria 2013 Demographic and Health Survey	77390	ICF International, National Population Commission of Nigeria. Nigeria Demographic and Health Survey 2013. Fairfax, United States of America: ICF International.	Point	38948	Gold Standard	Gold Standard	Gold Standard
Nigeria (Kaduna, Lagos) 2014 PMA2020 Round 1	256263	Centre for Population and Reproductive Health (CPRH), University of Ibadan; Centre for Research, Evaluation Resources and Development (CRERD); Population and Reproductive Health Program (PRHP), Obafemi Awolowo University (OAU); Bayero University Kano (BUK); and The Bill & Melinda Gates Institute for Population and Reproductive Health at The Johns Hopkins Bloomberg School of Public Health. Performance Monitoring and Accountability 2020 (PMA2020) Survey round 1, PMA2014/Nigeria-R1 (Kaduna & Lagos). 2014. Nigeria and Baltimore, Maryland, USA.	Polygon-Admin 1	3337	Gold Standard	Gold Standard	Gold Standard

Survey	NID*	Citation	Geographic Detail	Sample size (women age 15–49)**	Gold Standard / Crosswalk		
					Contraceptive Use	Need	Intention to Use
Nigeria (Kaduna, Lagos) 2015 PMA2020 Round 2	256268	Centre for Research, Evaluation Resources and Development (CRERD), Bayero University Kano (BUK), and The Bill & Melinda Gates Institute for Population and Reproductive Health at The Johns Hopkins Bloomberg School of Public Health. Performance Monitoring and Accountability 2020 (PMA2020) Survey round 2, PMA2015/Nigeria-R2 (Kaduna & Lagos). 2015. Nigeria and Baltimore, Maryland, USA	Polygon-Admin 1	4390	Gold Standard	Gold Standard	Gold Standard
Nigeria (National) 2016 PMA2020 Round 3	286022	Centre for Research, Evaluation Resources and Development (CRERD), Bayero University Kano (BUK), and The Bill & Melinda Gates Institute for Population and Reproductive Health at The Johns Hopkins Bloomberg School of Public Health. Performance Monitoring and Accountability 2020 (PMA2020) Survey round 3, PMA2016/Nigeria-R3 (National). 2016. Nigeria and Baltimore, Maryland, USA.	Polygon-Admin 1	11090	Gold Standard	Gold Standard	Gold Standard
Nigeria 2016–2017 Multiple Indicator Cluster Survey	437724	National Agency for the Control of AIDS (Nigeria), National Bureau of Statistics (Nigeria), National Primary Health Care Development Agency (NPHCDA) (Nigeria), United Nations Children's Fund (UNICEF). Nigeria Multiple Indicator Cluster Survey with National Immunization Coverage Survey Supplement 2016–2017 - National Bureau of Statistics. New York, United States of America: United Nations Children's Fund (UNICEF), 2018.	Point	34376	Gold Standard	Gold Standard	-

Survey	NID*	Citation	Geographic Detail	Sample size (women age 15–49)**	Gold Standard / Crosswalk		
					Contraceptive Use	Need	Intention to Use
Nigeria (National) 2017 PMA2020 Round 4	349780	Centre for Research, Evaluation Resources and Development (CRERD), Bayero University Kano (BUK), and The Bill & Melinda Gates Institute for Population and Reproductive Health at The Johns Hopkins Bloomberg School of Public Health. Performance Monitoring and Accountability 2020 (PMA2020) Survey round 4, PMA2017/Nigeria-R4 (National). 2017. Nigeria and Baltimore, Maryland, USA.	Polygon-Admin 1	11380	Gold Standard	Gold Standard	Gold Standard
Nigeria (Oyo) 2017 PMA2020 Round 1	394680	Bayero University Kano, Bill and Melinda Gates Institute for Population and Reproductive Health, Johns Hopkins Bloomberg School of Public Health, Center for Research, Evaluation, and Resource Development (CRERD), Federal Ministry of Health (Nigeria), Kaduna State Ministry of Health (Nigeria), Lagos State Ministry of Health (Nigeria), National Bureau of Statistics (Nigeria), National Population Commission of Nigeria. Nigeria - Oyo Performance Monitoring and Accountability 2020 Survey, Round 1 2017. Baltimore, United States of America: Bill and Melinda Gates Institute for Population and Reproductive Health, Johns Hopkins Bloomberg School of Public Health.	Polygon-Admin 1	1842	Gold Standard	Gold Standard	Gold Standard

Survey	NID*	Citation	Geographic Detail	Sample size (women age 15–49)**	Gold Standard / Crosswalk		
					Contraceptive Use	Need	Intention to Use
Nigeria (National) 2018 PMA2020 Round 5	394679	Centre for Research, Evaluation Resources and Development (CRERD), Bayero University Kano (BUK), and The Bill & Melinda Gates Institute for Population and Reproductive Health at The Johns Hopkins Bloomberg School of Public Health. Performance Monitoring and Accountability 2020 (PMA2020) Survey round 5, PMA2018/Nigeria-R5 (National). 2018. Nigeria and Baltimore, Maryland, USA.	Polygon-Admin 1	11106	Gold Standard	Gold Standard	Gold Standard
Nigeria 2018 Demographic and Health Survey	408484	Federal Ministry of Health (Nigeria), ICF International, National Population Commission (NPC). Nigeria Demographic and Health Survey 2018. Fairfax, United States of America: ICF International, 2020.	Point	41821	Gold Standard	Gold Standard	Gold Standard
Nigeria 2018 HIV/AIDS Indicator and Impact Survey	399235	Federal Ministry of Health, Nigeria AIDS Indicator and Impact Survey 2018 (NAIIS), Version 1.1. Data provided through the National Archive at NBS.	Polygon-Admin 1	88593	Gold Standard	Crosswalk 12	-
Nigeria (Kano, Lagos) 2020 PMA Phase 1	470500	Bayero University Kano, Bill and Melinda Gates Institute for Population and Reproductive Health, Johns Hopkins Bloomberg School of Public Health, Centre for Population and Reproductive Health, University of Ibadan (CPRH-UI), Centre for Research, Evaluation Resources and Development (CRERD) (Nigeria), Jhpiego, Population and Reproductive Health Program, Obafemi Awolowo University (PRHP-OAU). Nigeria - Kano and Lagos Performance Monitoring and Action Phase	Polygon-Admin 1	2591	Gold Standard	Gold Standard	Gold Standard

Survey	NID*	Citation	Geographic Detail	Sample size (women age 15–49)**	Gold Standard / Crosswalk		
					Contraceptive Use	Need	Intention to Use
		1 Household and Female Survey 2019–2020. Baltimore, United States of America: Johns Hopkins Bloomberg School of Public Health, 2021.					
Nigeria (Kano, Lagos) 2020 PMA Phase 2	493511	Bayero University Kano, Bill and Melinda Gates Institute for Population and Reproductive Health, Johns Hopkins Bloomberg School of Public Health, Centre for Research, Evaluation Resources and Development (CRERD) (Nigeria), Jhpiego. Nigeria - Kano and Lagos Performance Monitoring and Action Phase 2 Household and Female Survey 2020–2021. Baltimore, United States of America: Johns Hopkins Bloomberg School of Public Health, 2021.	Polygon-Admin 1	2617	Gold Standard	Gold Standard	Gold Standard

*NID = data source unique identifier in the Global Health Data Exchange (https://ghdx.healthdata.org/). Additional information about each data source is available via the GHDx, including information about the data provided and links to where the data can be accessed or requested (where available). NIDs can be entered in the search bar to retrieve the record for a particular source.
**Sample size = women age 15–49 with a non-missing sample weight.

Table S3: Surveys excluded from this analysis

Survey	NID*	Citation	Geographic Detail	Sample size (women age 15–49)**	Rationale for exclusion
Burkina Faso 2008 Global Fund Household Survey	26642	Global Fund to Fight Aids Tuberculosis and Malaria (GFATM). Burkina Faso Global Fund Household Health Coverage Survey 2008.	Polygon - Admin 3	9121	Data quality concerns due to inexplicable high rates of traditional contraception use and low rates of modern contraception use across the dataset.
Burkina Faso 2020 PMA Phase 1 Follow-up, 2020	470508	International Centre for Reproductive Health Kenya (ICRHK) and The Bill & Melinda Gates Institute for Population and Reproductive Health at The Johns Hopkins Bloomberg School of Public Health. Performance Monitoring and Accountability 2020 (PMA2020) Survey Round 7, PMA2018/Kenya-R7. 2018. Kenya and Baltimore, Maryland, USA.	Point	3524	Survey was conducted via phone interview from a sub-sample of women who completed phase 1, consented to a follow-up phone interview, and reported access to a phone. This shifts the demographics of women compared to phase 1, with proportionally more older women in urban areas participating.
Kenya 2009 Multiple Indicator Cluster Survey (Mombasa Informal Settlements)	56420	Kenya National Bureau of Statistics, United Nations Children's Fund (UNICEF). Kenya - Coast Multiple Indicator Cluster Survey 2009. New York, United States of America: United Nations Children's Fund (UNICEF), 2014.	Polygon	768	Sampled informal settlements only. Not likely to be representative of the general population living in Mombasa.
Kenya 2020 PMA Phase 1 Follow-up, 2020	470502	Bill and Melinda Gates Institute for Population and Reproductive Health, Johns Hopkins Bloomberg School of Public Health, International Center for Reproductive Health (Kenya), Jhpiego. Kenya Performance Monitoring and Action Phase 1 Female Follow-up Covid-19 Telephone Survey 2020. Baltimore,	Point	5976	Survey was conducted via phone interview from a sub-sample of women who completed phase 1, consented to a follow-up phone interview, and reported access to a phone. This shifts the demographics of women compared to phase 1, with proportionally more older women in urban areas participating.

Survey	NID*	Citation	Geographic Detail	Sample size (women age 15–49)**	Rationale for exclusion
		United States of America: Johns Hopkins Bloomberg School of Public Health, 2021.			
Nigeria 1999 Demographic and Health Survey	20555	Macro International, Inc, National Population Commission of Nigeria. Nigeria Demographic and Health Survey 1999. Calverton, United States of America: Macro International, Inc.	Polygon	5757	Data quality concerns for questions related to fertility and births within the last 5 years. Timing of recent births and fertility status could not be determined. Data collection was not completed by DHS, so they cannot support the data quality. This survey was excluded only from need calculations.
Nigeria 2003 Living Standards Survey	25006	Federal Office of Statistics (Nigeria). Nigeria Living Standards Survey 2003–2004.	Polygon- Admin 1	22622	Data quality concerns due to inconsistent contraception use at the admin 1 level. Nationally, contraception use was slightly low, but many admin 1s had implausibly high or low values.
Nigeria 2005 Reproductive Health, Child Health, and Education Household, School, and Health Facility Baseline Survey	50393	MEASURE Evaluation Project, Carolina Population Center, University of North Carolina, Center for Research, Evaluation, and Resource Development (CRERD), Center for Communication Programs, Bloomberg School of Public Health, Johns Hopkins, Creative Associates International, Constella Futures, Adolescent Health and Information Project (Nigeria), Federation of Muslim Women's Associations of Nigeria (FOMWAN), Nigerian Medical Association, Management Sciences for Health (MSH), Civil Society Action Coalition on Education For All. Nigeria Reproductive Health, Child Health, and	Polygon - Admin 2	2136	Data quality concern regarding intention to use contraception in the future. Low rates of intent were inexplicable after review of raw data and survey questions. This survey was excluded only from intent calculations.

Survey	NID*	Citation	Geographic Detail	Sample size (women age 15–49)**	Rationale for exclusion
		Education Household, School, and Health Facility Baseline Surveys 2005. Chapel Hill, United States: MEASURE Evaluation Project, Carolina Population Center, University of North Carolina.			
Nigeria 2006 General Household Survey	24890	Central Bank of Nigeria, National Bureau of Statistics (Nigeria), Nigerian Communications Commission (NCC). Nigeria General Household Survey 2007. Abuja, Nigeria: National Bureau of Statistics (Nigeria).	Polygon	19972	Current contraception usage could not be determined due to inconsistent data entry for two variables. Women who were not using contraception and who did not respond to contraception questions were marked as using "Other" for specific methods of contraception.
Nigeria 2007 Reproductive Health, Child Health, and Education Household, School, and Health Facility Survey	50426	MEASURE Evaluation Project, Carolina Population Center, University of North Carolina, Center for Research, Evaluation, and Resource Development (CRERD), Center for Communication Programs, Bloomberg School of Public Health, Johns Hopkins, Creative Associates International, Constella Futures, Adolescent Health and Information Project (Nigeria), Federation of Muslim Women's Associations of Nigeria (FOMWAN), Nigerian Medical Association, Management Sciences for Health (MSH), Civil Society Action Coalition on Education For All. Nigeria Reproductive Health, Child Health, and Education Household, School, and Health Facility Midline Surveys 2007. Chapel Hill,	Polygon - Admin 2	2327	Data quality concern regarding intention to use contraception in the future. Low rates of intent were inexplicable after review of raw data and survey questions. This survey was excluded only from intent calculations.

Survey	NID*	Citation	Geographic Detail	Sample size (women age 15–49)**	Rationale for exclusion
		United States: MEASURE Evaluation Project, Carolina Population Center, University of North Carolina.			
Nigeria 2007 National AIDS and Reproductive Health Survey	325046	Federal Ministry of Health (Nigeria), National Agency for the Control of AIDS (NACA) (Nigeria), National Bureau of Statistics (Nigeria), National Population Commission (NPC), Nigerian Institute of Medical Research, Society for Family Health (Nigeria), University College Hospital, Ibadan. Nigeria National HIV/AIDS and Reproductive Health Survey 2007.	Polygon - Admin 1	5360	Data quality concerns regarding need for contraception. At subnational levels, some areas reported inexplicably high levels of need due to a combination of high rates of cohabitation and lack of desire for children. Desire for children was not asked explicitly, but was captured in a question about why contraception was not being used. Also, the questionnaire skipped large sections if a women reported never having sex. This survey was excluded only from need and intent calculations.
Nigeria 2008 Living Standards Survey	151719	National Bureau of Statistics (Nigeria). Nigeria Living Standards Survey 2008–2010. Abuja, Nigeria: National Bureau of Statistics (Nigeria).	Polygon	74087	Data quality concerns regarding contraception usage. Certain regions have very high usage of injection as a contraception method and very low responses for traditional methods (as compared to 2007 DHS survey results from Nigeria).
Nigeria 2009 Reproductive Health, Child Health, and Education Household, School, and	50441	MEASURE Evaluation Project, Carolina Population Center, University of North Carolina, Center for Research, Evaluation, and Resource Development (CRERD), Center for Communication Programs, Bloomberg School of Public Health, Johns Hopkins, Creative Associates International, Futures Group	Polygon - Admin 2	2412	Data quality concern regarding intention to use contraception in the future. Low rates of intent were inexplicable after review of raw data and survey questions. This survey was excluded only from intent calculations.

Survey	NID*	Citation	Geographic Detail	Sample size (women age 15–49)**	Rationale for exclusion
Health Facility Survey		International, Adolescent Health and Information Project (Nigeria), Federation of Muslim Women's Associations of Nigeria (FOMWAN), Nigerian Medical Association, Management Sciences for Health (MSH), Civil Society Action Coalition on Education For All. Nigeria Reproductive Health, Child Health, and Education Household, School, and Health Facility End-of-Project Surveys. Chapel Hill, United States: MEASURE Evaluation Project, Carolina Population Center, University of North Carolina.			
Nigeria 2011 Multiple Indicator Cluster Survey	76703	National Bureau of Statistics (Nigeria), United Nations Children's Fund (UNICEF). Nigeria Multiple Indicator Cluster Survey 2011. New York, United States of America: United Nations Children's Fund (UNICEF), 2013.	Polygon- Admin 1	30772	Data quality control concerns for contraception use in three admin 1 areas. Contraception use rates were reasonable nationally and in most admin 1 areas, but Abia, Anambra, and Ebonyi had very high rates of contraception use. Data from these three regions were excluded from all indicator analyses.
Nigeria 2012 National HIV & AIDS and Reproductive Health Survey	324443	Expanded Social Marketing Project in Nigeria (ESMPIN), Federal Ministry of Health (Nigeria), Joint United Nations Program on HIV/AIDS (UNAIDS), National Population Commission (NPC), Society for Family Health (Nigeria), University College Hospital, Ibadan, World Health Organization (WHO). Nigeria National	Polygon - Admin 2	15639	Data quality concerns regarding contraception use. Nationally, the indicator was reasonable, but many admin 1 areas had implausibly high or low estimates.

Survey	NID*	Citation	Geographic Detail	Sample size (women age 15–49)**	Rationale for exclusion
		HIV/AIDS and Reproductive Health Survey 2012.			
Nigeria 2014 National Nutrition and Health Survey	274708	National Bureau of Statistics (Nigeria). National Nutrition and Health Survey 2014-v1.0.	Polygon - Admin 1	26557	Data quality concerns due to inexplicable high rates of traditional contraception use and low rates of modern contraception use across the dataset.
Nigeria 2015 National Nutrition and Health Survey	274707	National Bureau of Statistics (Nigeria). Nigeria National Nutrition and Health Survey 2015-v1.0.	Polygon - Admin 1	23688	Data quality concerns due to inexplicable high rates of traditional contraception use and low rates of modern contraception use across the dataset.
Nigeria (Kano, Lagos) 2020 PMA Phase 1 Follow-up, 2020	470503	Bayero University Kano, Bill and Melinda Gates Institute for Population and Reproductive Health, Johns Hopkins Bloomberg School of Public Health, Centre for Population and Reproductive Health, University of Ibadan (CPRH-UI), Centre for Research, Evaluation Resources and Development (CRERD) (Nigeria), Jhpiego, Population and Reproductive Health Program, Obafemi Awolowo University (PRHP-OAU). Nigeria - Kano and Lagos Performance Monitoring and Action Phase 1 Female Follow-up Covid-19 Telephone Survey 2020. Baltimore, United States of America: Johns Hopkins Bloomberg School of Public Health, 2021.	Polygon	1344	Survey was conducted via phone interview from a sub-sample of women who completed phase 1, consented to a follow-up phone interview, and reported access to a phone. This shifts the demographics of women compared to phase 1, with proportionally more older women in urban areas participating.

*NID = data source unique identifier in the Global Health Data Exchange (https://ghdx.healthdata.org/). Additional information about each data source is available via the GHDx, including information about the data provided and links to where the data can be accessed or requested (where available). NIDs can be entered in the search bar to retrieve the record for a particular source.

**Sample size = number of women age 15–49 with a non-missing sample weight.

Table S4: Extracted survey variables

**Variable***	**Type***	**Description / Coding***
ihme_loc_id	String	Location ID for country/region where survey was completed, (typically country code, but can be admin_1 region if survey was only completed in one region)
survey_module	String	Module completed with household (HH), household member (HHM), child (CH), woman (WM), or man (MN)
survey_name	String	Name of survey series
year_start	Numeric	Year data collection started
year_end	Numeric	Year data collection ended
strata	String	Stratification level
psu	String	Primary sampling unit
pweight	Numeric	Weight value for the individual
hhweight	Numeric	Weight value for the household
geospatial_id	String	Most detailed location information captured for each PSU, used for mapping
hh_id	Numeric	Identification for respondent household
sex	Numeric	Respondent is male (1) or female (2)
age_year	Numeric	Respondent age (years)
birth_date	String	Respondent birth date
birth_month	Numeric	Respondent birth month
birth_year	Numeric	Respondent birth year
int_date	String	Interview date
int_day	Numeric	Interview day
int_month	Numeric	Interview month
int_year	Numeric	Interview year
urban	Binary	Respondent lives in urban (1) or rural (0) location
admin_1	String	First-level administrative area where the respondent lives
admin_2	String	Second-level administrative area where the respondent lives

Variable*	Type*	Description / Coding*
curr_cohabit	Binary	Yes(1) or No(0) woman is currently married or living with a man ("in union")
former_cohabit	Binary	Yes(1) or No(0) woman was formerly married or living with a man ("in union"), but no longer is
pregnant	Binary	Yes(1) or No(0) woman is currently pregnant
never_used_contra	Binary	Yes(1) or No(0) woman said she has never used any contraceptive method (often a gateway to asking about current use of a method)
current_use	Binary	Yes(1) or No(0) whether a woman answered that she was currently using a contraceptive method (often a gateway to whether they ask about the specific method)
current_contra	String	Type of current contraceptive method
reason_no_contra	String	Reason why women are not using contraception if they aren't (some answers are usually related to infertility, although vaguer answers like "too old" or "infrequent sex", or ones outside the scope of this analysis like "fear of side-effects" will not be sufficient for designating infecundity)
marital_status	String	Marital status response, includes divorced and separated statuses
no_menses	Binary	Yes(1) or No(0) woman is not fertile (due to lack of recent menstruation, menopause, hysterectomy, or other infertility issues. A catch-all for any infecundity questions. Should be filled out in accordance with standard infecundity definitions for need for family planning)
desire_children_infertile	Binary	Yes(1) or No(0) woman said she was infertile when asked about her desire for future children (or more generally can be used for any variable where women respond that they are unable to have children)
last_menses	Numeric	The number of time units (specified in last_menses_unit) since the woman's last menstruation
last_menses_unit	Numeric	Code for the unit of timing since last menstruation (number expressed in last_menses)
sex_in_last_month	Binary	Yes(1) or No(0) woman has had sex in the last 30 days
last_sex	Numeric	The number of time units (specified in last_sex_unit) since the woman last had sexual intercourse
last_sex_unit	Numeric	Code for the unit of timing since last sexual intercourse (number expressed in last_sex)
desire_later	Binary	Yes(1) or No(0) woman said she does not want a child within the next 2 years (includes women who want no more children at all)
desire_soon	Binary	Yes(1) or No(0) woman said she wants a/another child soon/now

Variable*	Type*	Description / Coding*
desire_gate	Numeric	Code for an answer to the gateway question before asking about timing of desire for next child (often "do you intend to have a(nother) child in the future")
desire_gate_preg	Numeric	Code for an answer to the gateway question for pregnant women before asking about timing of desire for next child (often "do you intend to have a(nother) child in the future")
desire_timing	Numeric	The number of time units (specified in desire_unit) that they want to wait before having their next child
desire_unit	Numeric	Code for the unit of timing for desired next child (number expressed in desire_timing)
birth_in_last_two_years	Binary	Yes(1) or No(0) woman gave birth in the last 2 years
months_since_last_birth	Numeric	Months since woman last gave birth
last_birth_date	String	Date that woman last gave birth
last_birth_month	Numeric	Month that woman last gave birth
last_birth_year	Numeric	Year that woman last gave birth
interview_date	String	Date of survey interview
interview_month	Numeric	Month of survey interview
interview_year	Numeric	Year of survey interview
ppa_not_wanted	Binary	Yes(1) or No(0) woman said she wished she had delayed or prevented her most recent pregnancy/birth
preg_not_wanted	Binary	Yes(1) or No(0) pregnant woman said she wishes she had delayed or prevented her current pregnancy
recent_cohabit_start_date	String	Date that woman began her most recent marriage/union
in_first_cohabit	Binary	Yes(1) or No(0) woman is in her first marriage/union
years_since_first_cohabit	Numeric	Years that have elapsed since the woman's first marriage/union
first_cohabit_year	Numeric	Year that woman began her first marriage/union
first_cohabit_month	Numeric	Month that woman began her first marriage/union
intent_gate	String	Code for an answer to the gateway question before asking about timing of future intent to use contraception (often "do you intend to use contraception in the future")
intent_gate_preg	String	Code for an answer to the gateway question for pregnant women before asking about timing of future intent to use contraception (often "do you intend to use contraception in the future")

Variable*	Type*	Description / Coding*
intent_use	Binary	Yes(1) or No(0) whether a woman answered that she has intent to use a contraceptive method in the future (often a gateway to whether they ask about the specific method)
intent_unit	String	Code for the unit of timing for future contraceptive use (number expressed in intent_use)
intent_contra	String	Type of contraceptive method woman intends to use in the future
intent_reason_no_contra	String	Reason why women have no intention to use contraception if they don't

*Variable names, type, and coding vary from survey to survey and were mapped to what is described in this table for consistency across surveys.

Table S5: Crosswalk parameters

Crosswalk	Missing components	Indicator	$\beta_{Burkina\ Faso}$	β_{Kenya}	$\beta_{Nigeria}$	γ
1	Contraception use questions only asked to married/in union women.	Using Any Contraception	-0.2650920	-0.3544974	-0.3714786	0.0168742
1	Contraception use questions only asked to married/in union women.	Using Modern Methods	-0.1275306	0.0357251	-0.2373645	0
2	Lacks questions to determine ability to have a child during cohabitation and sexual activity.	In Need of Contraception	0.1429800	0.1340928	0.2317901	0
3	Lacks questions to determine ability to have a child during cohabitation and sexual activity. Cannot identify PPA women, or questions about desire of last birth were not asked to these women.	In Need of Contraception	0.6717420	0.2768307	0.7011292	0.0472051
4	Lacks questions to determine ability to have a child during cohabitation and sexual activity. Intent only captured for 12 month time period.	In Need of Contraception	0.1429800	0.1340928	0.2317901	0
4	Lacks questions to determine ability to have a child during cohabitation and sexual activity. Intent only captured for 12 month time period.	Intent to Use Contraception	-1.4118497	-1.1595927	-1.6328244	0.2491370
5	Lacks questions to determine ability to have a child during cohabitation and sexual activity. Cannot identify pregnant women, or questions about desire of current birth were not asked to these women. Cannot identify PPA women, or questions about desire of last birth were not asked to these women.	In Need of Contraception	0.5383769	0.0694587	0.6047103	0.0697460
6	Lacks questions to determine lack of menstruation due to infecundity. Lacks questions to determine ability to have a child during cohabitation and sexual activity. Cannot identify PPA women, or questions about desire of last birth were not asked to these women.	In Need of Contraception	0.8785203	0.4744768	0.8596354	0.0444670
7	Lacks questions to determine ability to have a child during cohabitation and sexual activity. Cannot identify pregnant women, or questions about desire of current birth were not asked to these	In Need of Contraception	-0.04742448	-0.2572004	0.0119612	0.00506274

Crosswalk	Missing components	Indicator	$\beta_{Burkina\ Faso}$	β_{Kenya}	$\beta_{Nigeria}$	γ
	women. Questions regarding desire for a child now/in the future only asked to married/in union women.					
8	Time since last sexual activity not captured. Lacks questions to determine lack of menstruation due to infecundity. Lacks questions to determine ability to have a child during cohabitation and sexual activity. Cannot identify PPA women, or questions about desire of last birth were not asked to these women.	In Need of Contraception	0.8161569	0.3356303	0.7638113	0.0675486
9	Time since last sexual activity not captured. No questions related to desire for a child now/in the future. Cannot identify pregnant women, or questions about desire of current birth were not asked to these women. Cannot identify PPA women, or questions about desire of last birth were not asked to these women.	In Need of Contraception	0.9022516	0.1760378	1.0297777	0.2164482
10	Time since last sexual activity not captured. Lacks questions to determine lack of menstruation due to infecundity. Lacks questions to determine ability to have a child during cohabitation and sexual activity. Cannot identify PPA women, or questions about desire of last birth were not asked to these women. Questions regarding desire of current pregnancy only asked to married/in union women.	In Need of Contraception	0.8049274	0.2905200	0.7532512	0.0712652
11	Time since last sexual activity not captured. No questions related to desire for a child now/in the future. Lacks questions to determine lack of menstruation due to infecundity. Lacks questions to determine ability to have a child during cohabitation and sexual activity. Cannot identify PPA women, or questions about desire of last birth were not asked to these women.	In Need of Contraception	1.52796784	0.7442419	1.6061468	0.1809528
12	Time since last sexual activity not captured. No questions related to desire for a child now/in the future. Lacks questions to determine infertility. Lacks questions to determine lack of menstruation due to infecundity. Lacks questions to determine ability to have a child during cohabitation and sexual activity. Cannot identify pregnant women, or questions about desire of	In Need of Contraception	1.5133932	0.6217869	1.5997712	0.2380716

Crosswalk	Missing components	Indicator	$\beta_{Burkina\ Faso}$	β_{Kenya}	$\beta_{Nigeria}$	γ
	current birth were not asked to these women. Cannot identify PPA women, or questions about desire of last birth were not asked to these women.					

Table S6: MBG model parameters

Indicator	Parameter	Burkina Faso			Kenya			Nigeria		
		**0.025th quantile**	**0.500th quantile**	**0.975th quantile**	**0.025th quantile**	**0.500th quantile**	**0.975th quantile**	**0.025th quantile**	**0.500th quantile**	**0.975th quantile**
$p^{anycontra}$	β_0	-1.963	0.000	1.962	-1.963	0.000	1.962	-1.963	0.000	1.962
	$\beta_{1,\mathrm{GAM}}$	-0.291	-0.073	0.145	0.128	0.341	0.553	0.260	0.414	0.567
	$\beta_{1,\mathrm{BRT}}$	0.455	0.636	0.816	0.321	0.489	0.656	0.223	0.358	0.493
	$\beta_{1,\mathrm{ridge}}$	0.267	0.437	0.607	0.010	0.170	0.331	0.104	0.228	0.352
	σ_{time}	0.122	0.195	0.301	0.084	0.133	0.192	0.033	0.073	0.130
	ρ_{space}	0.272	0.543	1.062	0.578	1.057	1.907	1.634	2.522	4.430
	σ_{space}	0.219	0.282	0.367	0.327	0.435	0.563	0.285	0.384	0.542
	σ_{survey}	0.247	0.309	0.388	0.174	0.207	0.244	0.627	0.679	0.746
	σ_{nugget}	0.442	0.482	0.513	0.302	0.323	0.343	0.208	0.228	0.251
$p^{modcontra}$	β_0	-1.963	0.000	1.962	-0.475	0.040	0.555	-1.963	0.000	1.962
	$\beta_{1,\mathrm{GAM}}$	-0.346	-0.069	0.209	-0.073	0.192	0.459	-0.485	-0.247	-0.011
	$\beta_{1,\mathrm{BRT}}$	0.287	0.516	0.744	0.180	0.382	0.584	0.235	0.450	0.665
	$\beta_{1,\mathrm{ridge}}$	0.281	0.553	0.823	0.198	0.426	0.653	0.591	0.798	1.005
	σ_{time}	0.355	0.528	0.794	0.075	0.108	0.145	0.029	0.097	0.225
	ρ_{space}	0.254	0.569	1.385	1.231	2.084	3.634	0.558	1.162	2.792
	σ_{space}	0.442	0.691	1.064	0.581	0.853	1.292	0.169	0.234	0.314
	σ_{survey}	0.276	0.432	0.642	0.481	0.783	1.154	0.686	0.752	0.827
	σ_{nugget}	0.779	0.888	1.003	0.55	0.621	0.677	0.005	0.025	0.068
p^{need}	β_0	-1.963	0.000	1.962	-1.963	0.000	1.962	-1.963	0.000	1.962
	$\beta_{1,\mathrm{GAM}}$	0.020	0.641	1.261	-0.413	-0.147	0.118	0.093	0.309	0.525
	$\beta_{1,\mathrm{BRT}}$	-0.374	0.177	0.729	0.429	0.628	0.828	-0.123	0.076	0.275

Indicator	Parameter	Burkina Faso			Kenya			Nigeria		
		**0.025th quantile**	**0.500th quantile**	**0.975th quantile**	**0.025th quantile**	**0.500th quantile**	**0.975th quantile**	**0.025th quantile**	**0.500th quantile**	**0.975th quantile**
	$\beta_{1,\mathrm{ridge}}$	-0.207	0.181	0.569	0.314	0.519	0.723	0.429	0.615	0.801
	σ_{time}	0.009	0.045	0.127	0.022	0.064	0.125	0.083	0.144	0.227
	ρ_{space}	1.182	8.926	99.583	0.410	0.919	2.209	0.836	1.827	3.465
	σ_{space}	0.043	0.185	0.959	0.148	0.197	0.269	0.081	0.116	0.163
	σ_{survey}	0.244	0.291	0.349	0.294	0.327	0.368	0.484	0.538	0.591
	σ_{nugget}	0.197	0.228	0.261	0.219	0.252	0.288	0.058	0.092	0.140
	β_0	-1.963	0.000	1.962	-1.236	-0.908	-0.585	-1.963	0.000	1.962
	$\beta_{1,\mathrm{GAM}}$	-0.277	0.202	0.682	-0.185	0.148	0.481	0.295	0.613	0.931
	$\beta_{1,\mathrm{BRT}}$	0.114	0.555	0.995	-0.170	0.089	0.348	-0.389	-0.127	0.136
	$\beta_{1,\mathrm{ridge}}$	-0.235	0.243	0.719	0.511	0.763	1.014	0.291	0.514	0.736
p^{intent}	σ_{time}	0.036	0.126	0.280	0.000	0.003	0.005	0.439	0.592	0.800
	ρ_{space}	0.606	3.680	32.708	3.779	6.847	15.305	0.670	1.172	2.229
	σ_{space}	0.000	0.003	0.013	0.687	1.081	1.917	0.267	0.342	0.438
	σ_{survey}	0.675	0.803	0.943	0.059	0.125	0.248	0.174	0.265	0.398
	σ_{nugget}	0.476	0.524	0.588	0.340	0.411	0.497	0.016	0.068	0.154

Table S7: Model validation results

Indicator	Country	Mean Error (PPT)		95% Coverage (%)		RMSE – Country (PPT)		RMSE – Admin. 1 (PPT)		RMSE – Admin. 2 (PPT)	
		In-sample	Out-of-sample	In-sample	Out-of-sample	In-sample	Out-of-sample	In-sample	Out-of-sample	In-sample	Out-of-sample
$p^{anycontra}$	Burkina Faso	-0.5	-0.5	97.1	95.4	1.2	1.2	3.2	3.5	4.6	5.1
	Kenya	-0.3	-0.3	96.6	95.7	2.7	2.9	4.2	4.6	7	7.5
	Nigeria	0.3	0.2	91.6	90.9	2.2	2.3	4.0	4.1	5.9	6.1
$p^{modcontra}$	Burkina Faso	1.8	1.7	98.9	98.1	2.9	3.0	4.3	5.1	6.7	7.8
	Kenya	-0.7	-0.8	98.1	97.5	2.8	2.8	5.4	5.6	7.5	7.8
	Nigeria	-1.6	-1.5	94.6	94.1	4.2	4.2	9.2	9.3	14.1	14.3
p^{need}	Burkina Faso	-1.6	-1.5	94.8	94.4	3.5	3.5	5.4	5.4	6.4	6.5
	Kenya	-0.5	-0.6	95.4	94.4	3.1	3.3	5.2	5.5	7.9	8.3
	Nigeria	1.6	1.4	92.2	92.1	4.3	4.3	6.2	6.1	8.6	8.6
p^{intent}	Burkina Faso	1.5	1.4	98.1	97.2	4.2	4.4	10.1	10.4	12.7	13
	Kenya	1.1	1.1	98.3	97.7	1.7	1.7	8.2	8.6	14.9	15.4
	Nigeria	-0.1	-0.1	98.3	97.6	1.4	1.5	3.2	3.6	13.5	14.7

Authors' Contributions

Managing the overall research enterprise
Laura Dwyer-Lindgren and Erin C Mullany.

Writing the first draft of the manuscript
Laura Dwyer-Lindgren, Susan McLaughlin, and Doori Oh.

Primary responsibility for applying analytical methods to produce estimates
Rebecca M Cogen and Doori Oh.

Primary responsibility for seeking, cataloguing, extracting, or cleaning data; designing or coding figures and tables
Rebecca M Cogen and Doori Oh.

Providing data or critical feedback on data sources
Abiola Victor Adepoju, Aanuoluwapo Adeyimika Afolabi, Olufemi Ajumobi, Dickson A Amugsi, Olivia Angelino, Tesleem Kayode Babalola, Rebecca M Cogen, Michael Ekholuenetale, Adeniyi Francis Fagbamigbe, Muktar A Gadanya, Augustine Mwangi Gatotoh, Simon I Hay, Segun Emmanuel Ibitoye, Charity Ehimwenma Joshua, Gbenga A Kayode, Shafiu Mohammed, Aggrey Gisiora Mokaya, Christopher J L Murray, Josephine W Ngunjiri, Julius Nyerere Odhiambo, Onome Bright Oghenetega, Abiola Ogunkoya, Doori Oh, Patrick Godwin Okwute, Andrew T Olagunju, Bolajoko Olubukunola Olusanya, Jacob Olusegun Olusanya, Obinna E Onwujekwe, Mayowa O Owolabi, and Shehu Salihu Umar.

Developing methods or computational machinery
Olivia Angelino, Rebecca M Cogen, Laura Dwyer-Lindgren, Muktar A Gadanya, Simon I Hay, Christopher J L Murray, Josephine W Ngunjiri, and Doori Oh.

Providing critical feedback on methods or results
Olumide Abiodun, Lawan Hassan Adamu, Abiola Victor Adepoju, Miracle Ayomikun Adesina, Daniel Adedayo Adeyinka, Aanuoluwapo Adeyimika Afolabi, Olufemi Ajumobi, Dickson A Amugsi, Tesleem Kayode Babalola, Manaseh A Bocha, Isaac Sunday Chukwu, Michael Ekholuenetale, Adeniyi Francis Fagbamigbe, Morenike Oluwatoyin Folayan, Muktar A Gadanya, Augustine Mwangi Gatotoh, Annie Haakenstad, Simon I Hay, Segun Emmanuel Ibitoye, Olayinka Stephen Ilesanmi, Kenneth Chukwuemeka Iregbu, Charity Ehimwenma Joshua, Gbenga A Kayode, Peter M Macharia, Shafiu Mohammed, Aggrey Gisiora Mokaya, Erin C Mullany, Christopher J L Murray, Josephine W Ngunjiri, Julius Nyerere Odhiambo, Oluwakemi Ololade Odukoya, Onome Bright Oghenetega, Abiola Ogunkoya, Doori Oh, Akinkunmi Paul Okekunle, Patrick Godwin Okwute, Andrew T Olagunju, Isaac Iyinoluwa Olufadewa, Bolajoko Olubukunola Olusanya, Jacob Olusegun Olusanya, Obinna E Onwujekwe, Mayowa O Owolabi, Mu'awiyyah Babale Sufiyan, Shehu Salihu Umar, Chukwuma David Umeokonkwo, Yohannes Dibaba Wado, and Hadiza Yusuf.

Drafting the work or revising is critically for important intellectual content
Olumide Abiodun, Lawan Hassan Adamu, Abiola Victor Adepoju, Aanuoluwapo Adeyimika Afolabi, Olufemi Ajumobi, Dickson A Amugsi, Laura Dwyer-Lindgren, Adeniyi Francis Fagbamigbe, Morenike Oluwatoyin Folayan, Muktar A Gadanya, Simon I Hay, Segun Emmanuel Ibitoye, Olayinka Stephen Ilesanmi, Kenneth Chukwuemeka Iregbu, Gbenga A Kayode, Peter M Macharia, Susan McLaughlin, Shafiu Mohammed, Aggrey Gisiora Mokaya, Josephine W Ngunjiri, Julius Nyerere Odhiambo, Oluwakemi Ololade

Odukoya, Onome Bright Oghenetega, Abiola Ogunkoya, Doori Oh, Akinkunmi Paul Okekunle, Patrick Godwin Okwute, Andrew T Olagunju, Babayemi Oluwaseun Olakunde, Bolajoko Olubukunola Olusanya, Jacob Olusegun Olusanya, Obinna E Onwujekwe, Mayowa O Owolabi, Mu'awiyyah Babale Sufiyan, Chukwuma David Umeokonkwo, and Hadiza Yusuf.

Managing the estimation or publications process

Laura Dwyer-Lindgren, Simon I Hay, Erin C Mullany, Christopher J L Murray, Bolajoko Olubukunola Olusanya, and Jacob Olusegun Olusanya.
